# Highly Potent Host-Specific Small-Molecule Inhibitor of Paramyxovirus and Pneumovirus Replication with High Resistance Barrier

**DOI:** 10.1128/mBio.02621-21

**Published:** 2021-11-02

**Authors:** Neeta Shrestha, Flavio Max Gall, Cyrille Mathieu, Melanie Michaela Hierweger, Melanie Brügger, Marco P. Alves, Jonathan Vesin, Damiano Banfi, David Kalbermatter, Branka Horvat, Marc Chambon, Gerardo Turcatti, Dimitrios Fotiadis, Rainer Riedl, Philippe Plattet

**Affiliations:** a Division of Neurological Sciences, Vetsuisse Faculty, University of Bern, Bern, Switzerland; b Institute of Chemistry and Biotechnology, Center for Organic and Medicinal Chemistry, Zurich University of Applied Sciences (ZHAW), Wädenswil, Switzerland; c CIRI, Centre International de Recherche en Infectiologie, Team Immunobiology of the Viral Infections, Univ Lyon, INSERM, U1111, CNRS, UMR5308, Université Claude Bernard Lyon 1, Ecole Normale Supérieure de Lyon, Lyon, France; d Institute of Virology and Immunology, Bern, Switzerland; e Department of Infectious Diseases and Pathobiology, Vetsuisse Faculty, University of Bern, Bern, Switzerland; f Graduate School for Cellular and Biomedical Sciences, University of Bern, Bern, Switzerland; g Biomolecular Screening Facility, Ecole Polytechnique Fédérale de Lausanne (EPFL), Lausanne, Switzerland; h Institute of Biochemistry and Molecular Medicine, and Swiss National Centre of Competence in Research (NCCR) TransCure, University of Bern, Bern, Switzerland; Mayo Clinic; Johns Hopkins Bloomberg School of Public Health

**Keywords:** paramyxovirus, pneumovirus, host-directed, replication, inhibitors, high resistance barrier

## Abstract

Multiple enveloped RNA viruses of the family *Paramyxoviridae* and *Pneumoviridae,* like measles virus (MeV), Nipah virus (NiV), canine distemper virus (CDV), or respiratory syncytial virus (RSV), are of high clinical relevance. Each year a huge number of lives are lost as a result of these viral infections. Worldwide, MeV infection alone is responsible for over a hundred thousand deaths each year despite available vaccine. Therefore, there is an urgent need for treatment options to counteract these viral infections. The development of antiviral drugs in general stands as a huge challenge due to the rapid emergence of viral escape mutants. Here, we disclose the discovery of a small-molecule antiviral, compound 1 (ZHAWOC9045), active against several pneumo-/paramyxoviruses, including MeV, NiV, CDV, RSV, and parainfluenza virus type 5 (PIV-5). A series of mechanistic characterizations revealed that compound 1 targets a host factor which is indispensable for viral genome replication. Drug resistance profiling against a paramyxovirus model (CDV) demonstrated no detectable adaptation despite prolonged time of investigation, thereby mitigating the rapid emergence of escape variants. Furthermore, a thorough structure-activity relationship analysis of compound 1 led to the invention of 100-times-more potent-derivatives, e.g., compound 2 (ZHAWOC21026). Collectively, we present in this study an attractive host-directed pneumoviral/paramyxoviral replication inhibitor with potential therapeutic application.

## INTRODUCTION

While the urgent need for antivirals against ongoing infections is undeniable, one of the lessons learned from the current COVID-19 pandemic is the need for broad-spectrum antivirals that are readily deployable for the prevention of future outbreaks (1). *Paramyxoviridae* and *Pneumoviridae* are among the families of single-stranded, negative-sense RNA viruses that continue to pose a severe disease burden globally. Moreover, a risk of a future pandemic mediated by a potential cross-species transmission of widely distributed paramyxoviruses is very much a possibility (2). Among the paramyxoviruses, measles virus (MeV) undoubtedly stands as one of the important viruses as it accounts for the loss of over 100,000 human lives each year. Despite the availability of an effective vaccine, the numbers of measles cases and deaths are on the rise every year with a decade-high of measles case numbers in 2019 (3) owing to the drastic drop in vaccination (4). Canine distemper virus (CDV), another important paramyxovirus, is also a highly contagious virus with an extensively wide host range within the order Carnivora, namely, *Canidae*, *Hyaenidae*, *Phocidae*, *Felidae*, *Procyonidae*, *Mustelidae*, *Ursidae*, and *Viverridae.* While a vaccine against CDV exists, its efficacy depends on the targeted species, which in turn poses a challenge (5). Respiratory syncytial virus (RSV), the most common cause of acute lower respiratory infection (ALRI) in children younger than 5 years, was estimated to have killed 66,000 to 199,000 children in 2005, with 99% of these deaths occurring in developing countries (6). Despite the disease burden, various reasons (7) have hindered the development of more effective, better-tolerated, and affordable antivirals against RSV. Nipah virus (NiV), another deadly paramyxovirus, stands out as a potential pandemic threat (8). Despite its high case-fatality rate, there is still no treatment available for either humans or animals.

Therapeutic candidates with qualities such as being cost-effective, shelf-stable at room temperature, compatible with oral administration, and safe for prophylactic use in pediatric patients are deemed promising (9). Small therapeutic molecules, among others, potentially meet these requirements. Against paramyxoviruses, no first-in-class antiviral has been approved so far, despite several approaches reported earlier (10–14). Among the best lead compounds, viral RNA-dependent RNA-polymerase (RdRp)-directed inhibitors (e.g., ERDRP-0519) exhibited potent efficacy against MeV and CDV both *in vitro* and *in vivo* (15–17). Recently, another potent antiparamyxovirus RdRp compound (GHP-88309) was identified and exhibited attractive profiles *in vivo* (18). Although these virus-directed antiviral compounds look promising, monotherapy against such highly mutable RNA viruses still faces the key challenge of generation of escape mutants. Interestingly, in 2011, a host-directed myxoviral inhibitor, namely, JMN3-003, had been discovered that has a broad-spectrum activity against many different viruses (19). However, the further development of the compound remained unreported. In the case of RSV, of many discovered antivirals, fusion (F)-protein inhibitors such as presatovir in hematopoietic cell transplant (HCT) patients with upper respiratory tract (URT) illness and ziresovir and JNJ-53718678 in hospitalized infants have yielded encouraging results (7, 20–22). However, the possibility of frequent emergence of F-protein amino acid mutations might reduce the drug susceptibility (7).

Among the novel approaches for antiviral drug development, host-directed therapies have gained popularity over the last 2 decades. In contrast to the conventional approach of targeting virus-encoded factors, development of antiviral drugs targeting host factors (23) acts as an alternative therapeutic strategy toward mitigating the challenge of emergence of drug-resistant viruses. Moreover, given the fact that related viruses may share common host cell pathways needed for their life cycle, host-directed antiviral approaches hold the benefit of demonstrating potential broad-spectrum efficacy.

In this study, we present the identification, characterization, and optimization of a small antiviral molecule, compound 1 (ZHAWOC9045), discovered using a cell-based bioassay we recently described combined with high-throughput screening (HTS) (24). The compound exhibits broad-spectrum inhibitory activity against several members of the *Paramyxoviridae* family (e.g., MeV, CDV, NiV, and PIV-5) and the *Pneumoviridae* family (e.g., RSV). Through detailed structure-activity relationship (SAR) studies, the structure of compound 1 was successfully optimized to result in highly potent derivatives characterized to have strong efficacy at very low concentrations of nanomolar range against a panel of enveloped pathogenic RNA viruses.

## RESULTS

### Hit identification with potent antiviral activity.

**FIG 1:**
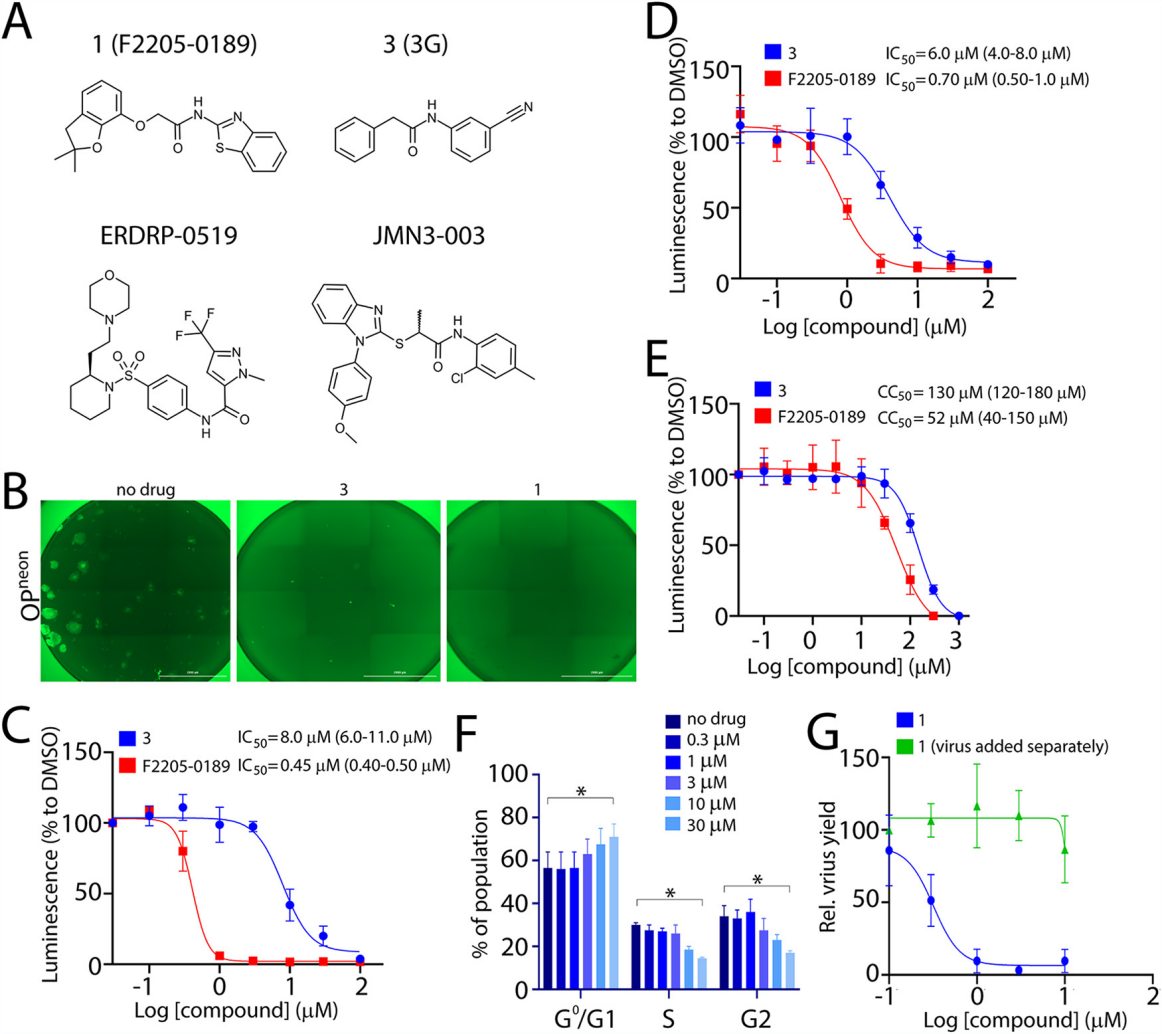
Discovery of a novel small-molecule antiviral. (A) Structure of compound 1 (F2205-0189), compound 3 (3G), ERDRP-0519 (16), and JMN3-003 (19). (B) Assessment of the compounds’ inhibitory impact on virus-induced syncytium formation. Microscopic images of cells infected with OP^neon^ in the presence of the compounds. Scale bars, 2,000 μm. Pictures were captured with a Cytation 5 imaging multimode reader (BioTek). Note that due to automatic settings, discrepancy in background intensity measurement is visible in the stitched images. (C) IC_50_ measurement of compounds against the attenuated OP-CDV strain. (D) IC_50_ measurement of compounds against the wild-type A75/17-CDV. (E) Measurement of the cytotoxic effect of the inhibitors. Ninety-five percent confidence intervals are shown in parentheses. Relative luminescence values were normalized for values obtained in the presence of DMSO control and represent means from three independent experiments. (F) Impact of compound 1 on cell cycle progression of treated cells. Vero cells were incubated in the absence or presence of increasing concentration of compound 1 for 42 h and analyzed using flow cytometry. The values show the means ± SD from three independent experiments. Dunnett’s multiple-comparison test was applied after two-way analysis of variance (ANOVA) (*, *P* < 0.05). (G) Assessment of viral proliferation after incubation with the compound. Vero cells expressing canine SLAM (cSLAM) were infected with wild-type A75/17-CDV (A75^neon/nLucP^) at an MOI of 0.01 in the presence of increasing concentrations of the compound. After 30 h, infected cells were frozen and thawed (twice), and viruses in the lysates were harvested and titrated in Vero-cSLAM cells.

The high-throughput screening (HTS) performed previously to identify novel entry inhibitors (24) also yielded several compounds that were observed to enhance fusogenic activity (see Fig. S1A in the supplemental material). To further understand the mechanisms by which those compounds could potentially increase membrane fusion, around 20 compounds were cherry-picked and counterscreened at the concentration of 10 μM using a recombinant attenuated CDV strain, Onderstepoort (OP) expressing the mNeonGreen fluorescent protein (neon) as a reporter (OP^neon^). While no compound could be confirmed as a significant fusion-inducer in the context of viral infection, to our surprise, compound 1 (F2205-0189 from Life Chemicals) presented a rather notable antiviral activity (Fig. 1A and B). In order to determine the potency of the compound, the 50% inhibitory concentration (IC_50_) of compound 1 was measured by employing either the attenuated OP-CDV strain (OP^neon/nLucP^) or the wild-type A75/17 (A75)-CDV strain (A75^neon/nLucP^) (Fig. 1C and D). The activity of the compound was compared with the previously described entry inhibitor compound 3 (3G) (Fig. 1A) in Vero cells expressing the canine signaling lymphocyte activation molecule (cSLAM) receptor (Vero-cSLAM). Note that both recombinant viruses expressed the mNeonGreen (neon) and the nanoluciferase (nLucP) reporter proteins (24). Remarkably, compound 1 exhibited a potent inhibitory activity, with IC_50_ values of about 0.45 μM and 0.70 μM for OP-CDV and wild-type A75/17, respectively, compared to compound 3 with 8 μM (Fig. 1C and D).

10.1128/mBio.02621-21.1 FIG S1 (A) Laboratory Information Management System (LIMS) plot showing the result of small-molecule screening from the CDC library. The positive control is normalized to have a score of 1 (equivalent to 100% inhibition), and the negative control is normalized to have a score of 0 (equivalent to 0% inhibition + 100% activity). Green dots indicate the positive control (3G); cyan dots represent the negative control (DMSO); dark blue dots show the compounds screened. Red dots indicate the discovered compound (in duplicates). Yellow dots show the compounds selected for counterscreen. While the light blue window represents the 3 standard deviations for the negative controls, the green window is the equivalent for the positive control. (B) Confirmation of inhibitory activity of F2205-0189 upon resynthesis of compound 1 (ZHAWOC9045). The IC_50_ value of ZHAWOC9045 was measured against the attenuated OP-CDV strain in Vero-cSLAM cells. Ninety-five percent confidence intervals are shown in parentheses. Download FIG S1, TIF file, 2.5 MB. Copyright © 2021 Shrestha et al. 2021 Shrestha et al. https://creativecommons.org/licenses/by/4.0/ This content is distributed under the terms of the Creative Commons Attribution 4.0 International license.

In order to measure the cytotoxic activity of the compound, cell viability in the presence of compound 1 or 3 was investigated using the RealTime-Glo MT cell viability assay. Compound 1 presented a 50% cytotoxic concentration (CC_50_) value of about 50 μM (Fig. 1E). Compound 1 exhibited a specificity index (SI; CC_50_/IC_50_) of ∼100, whereas compound 3 displayed an SI value of about ∼16 (Table 1). Additionally, we investigated the impact of compound 1 on the cell cycle progression. To this aim, Vero-cSLAM cells were treated with increasing concentrations of the compound and the cell cycle was analyzed by flow cytometry (Fig. 1F). The data revealed that the compound causes the cell cycle arrest at G_1_ stage. However, the impact was statistically significant only at concentrations as high as 30 μM, about 60 times higher than the IC_50_ value.

**TABLE 1:** Comparison of SI values of initial hit 1 and optimized derivative 2 against several members of families *Paramyxoviridae* and *Pneumoviridae*^*a*^

Family	Virus	Cell line	Species	Compound 1			Compound 2		
				IC_50_ (μM)	CC_50_ (μM)	SI	IC_50_ (μM)	CC_50_ (μM)	SI
*Paramyxoviridae*	CDV (A75)	Vero-cSLAM	Monkey	0.70	52.0	74	0.0032	54.0	17,000
	CDV (OP)	Vero-cSLAM	Monkey	0.45	52.0	120	0.0026	54.0	20,000
	CDV (OP)	Vero-cNectin-4	Monkey	0.43	43.0	100	ND	ND	ND
	CDV (OP)	Vero	Monkey	0.40	46.0	120	0.0030	52.0	17,000
	CDV (OP)	MDCK	Canine	30.00	99.0	3.3	0.0650	87.0	1,300
	CDV (OP)	P114	Canine	2.60	57.0	22	0.0190	65.0	3,400
	CDV (OP)	Bsr-T7	Hamster	7.30	50.0	6.8	0.0120	ND	ND
	MeV (Edm)	Vero	Monkey	0.25	46.0	180	ND	ND	ND
	MeV (Edm)	NCI-H358	Human	0.52	50.0	96	ND	ND	ND
	MeV (ICB)	NCI-H358	Human	0.37	50.0	140	ND	ND	ND
	NiV	PGSA745-EFNB2	Hamster	ND	ND	ND	0.0800	80.0	1,000
	PIV-5	Vero	Monkey	7.20	46.0	6.4	0.0150	52.0	3,500

*Pneumoviridae*	RSV	HEp-2	Human	5.90	110.0	19	0.0310	51.0	1,700

*Rhabdoviridae*	VSV	Vero	Monkey	NA	46.0	NA	NA	46.0	NA

a Abbreviations: NA, not applicable; ND, not determined.

In order to rule out the possibility that reduction of the signal was due to an impact on the reporter protein, the effect of the compound was assessed on progeny virus production. As expected, reduction of the viral yield corresponded with the reduction of the luminescence signal (Fig. 1G), which validated the antiviral activity of compound 1. To exclude that preexisting compound (employed for the experiments) might have affected the subsequent viral titration, we also collected the lysates (cells plus supernatants) from noninfected but compound 1-treated cells and incubated them with virus. Indeed, the preexisting compounds did not exhibit any impact on the outcome of the viral titration analysis (Fig. 1G).

### Resynthesis of screening hit: compound 1 (ZHAWOC9045/F2205-0189).

**FIG 2:**
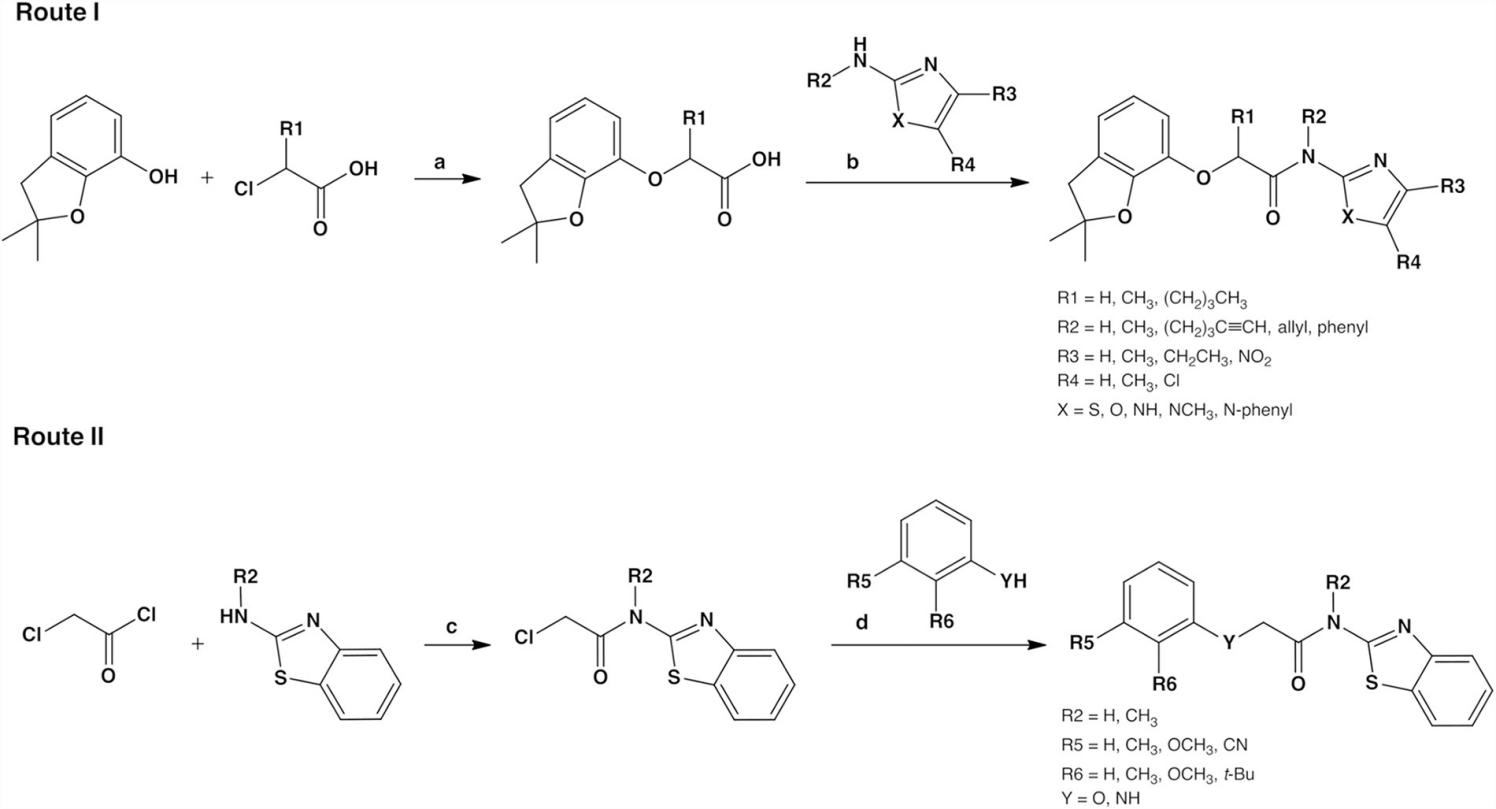
General synthesis routes for the optimization of compound 1. We used two main strategies to synthesize most of the new inhibitors. In route I, we first built the ether moiety and coupled the aminothiazole moiety in the last step. This allows a convenient variation of the thiazole moiety. In route II, we first coupled this thiazole moiety followed by the introduction of the phenol building block to vary the other side of the scaffold. (a) NaOH in water at 100°C for 45 min (49). (b) *N*,*N*-Diisopropylethylamine (DIPEA) and *N,N,N′,N′*-tetramethyl-*O*-(7-azabenzotriazol-1-yl)uronium hexafluorophosphate (HATU) in dimethylformamide (DMF) at 0°C to room temperature (RT) for 1 h. (c) Triethylamine (TEA) in dichloromethane (DCM) at 0°C to RT for 1 h. (d) Cs_2_CO_3_ and NaI in DMF at 60°C overnight.

To rule out false-positive results, the identity of the hit compound was confirmed by chemical resynthesis (Fig. 2, route I). The reaction of the phenol building block with chloroacetic acid resulted in the ether 4 with the carboxylic acid, which was coupled to 2-amino-benzothiazole to form the amide in compound 1. This compound was found to be as effective against CDV (Fig. S1B) as the purchased screening compound and was therefore employed for all the following experiments.

### Compound 1 targets a postentry step of the viral life cycle.

We next explored the mode of action of compound 1. To investigate whether compound 1 acts on the F-protein as compound 3 does, we determined the inhibitory activity against a compound 3-escape mutant virus, namely, OP^neon^/F-V575C (25). Vero-cSLAM cells were infected with the mutant virus in either the presence or absence of compound 1 or 3. While OP^neon^/F-V575C successfully escaped compound 3, the virus remained, however, highly sensitive to compound 1 (Fig. S2A). The absence of cross-resistance strongly supported the idea that compound 1 inhibited viral infection through a different molecular mechanism. To further validate these findings, the impact of compound 1 on the conformational stability of prefusion F-trimers was investigated. Indeed, it was demonstrated that compound 3 increased prefusion F-protein’s stability, thereby blocking the structural refolding necessary to fuse lipid membranes (26). The membrane-anchored OP-CDV F-protein was submitted to a brief heat shock (10 min at 65°C; a surrogate of F-activation) or kept at 37°C in the presence or absence of compounds 1 and 3. The conformation of F-protein was probed as reported previously (26), using a prefusion-specific monoclonal antibody (anti-Pre) followed by flow cytometry analyses to obtain semiquantitative data. While compound 3 indeed exhibited a strong stabilizing effect on prefusion F-trimers, compound 1 did not (Fig. S2B). Collectively, these experiments further suggested that compound **1** did not interfere with the refolding of the F-protein from the prefusion to the postfusion conformation.

10.1128/mBio.02621-21.2 FIG S2 Compound 1 inhibits the viral infection at postentry level. (A) The IC_50_ value of compound 1 was measured compared to 3G against OP^neon^/F-V575C (3G escape variant). (B) Heat shock resistance profiles of pre-fusion F conformation in presence or absence of the compounds. Selected F-proteins were expressed in Vero cells for 24 h. Cells were then subjected to brief heat shock (5 min) at 37°C or 65°C in the absence or presence of either compound 3 (75 μM) or compound 1 (10 μM). Conformational probing of surface-exposed F-proteins was performed using two different monoclonal antibodies (MAbs): anti-FLAG (conformation independent) and anti-Pre (prefusion specific). Upon addition of Alexa Fluor-conjugated secondary antibody, F-expressing cells were detached and submitted to flow cytometry analyses to record quantitative data. Values obtained for anti-Pre-stained F-protein were normalized to values obtained for anti-FLAG-stained F-protein. Means ± standard deviations of data from three independent experiments are shown. NA, not applicable. Download FIG S2, TIF file, 2.7 MB. Copyright © 2021 Shrestha et al. 2021 Shrestha et al. https://creativecommons.org/licenses/by/4.0/ This content is distributed under the terms of the Creative Commons Attribution 4.0 International license.

**FIG 3:**
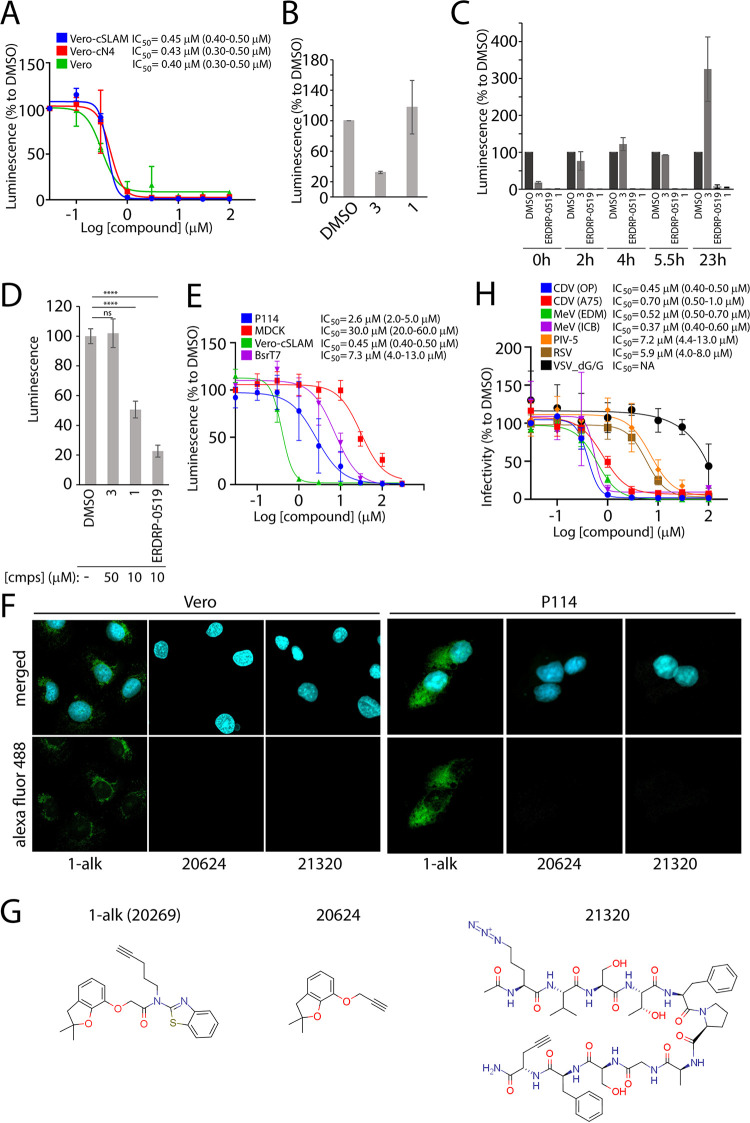
Investigation of mode of action of compound 1. (A) IC_50_ measurement of compound 1 against the attenuated OP-CDV strain in either Vero cells or Vero cells expressing SLAM (cSLAM) or Nectin4 (cN4) receptors. (B) Investigation of virucidal effect of compound 1. As a positive control, compound 3 as a virucidal compound was added. (C) OP-CDV time-of-addition studies. Compound 3 (entry inhibitor) and ERDRP-0519 (replication inhibitor) were taken as references. Relative luminescence was measured after 48 h of initial infection. Relative luminescence values were normalized for values obtained in the presence of DMSO control and represent means ± SD from three independent experiments. (D) Plasmid-based minigenome luciferase assay to determine the bioactivity of CDV polymerase complex. Relative luminescence values were normalized for values obtained in the presence of DMSO control and represent means ± SD from three independent experiments. Statistical significance of differences was determined using one-way ANOVA followed by Dunnett’s multiple-comparison test (****, *P* < 0.0001; ns, not significant). (E) Antiviral activity of compound 1 is host cell species specific. IC_50_ values of compound 1 were measured against the attenuated OP-CDV strain in cell lines of different species origin. Ninety-five percent confidence intervals are shown in parentheses. (F) Intracellular localization of alkyne-tagged compound 1 (1-alk) assessed through a click reaction using azide-linked Alexa Fluor 488 in Vero and P114 cell lines. 20624 and 21320 were used as negative controls. Green shows Alexa Fluor 488, and blue shows DAPI (nuclear staining). (G) Structure of three alkyne-tagged compounds. (H) Compound 1 displays broad-spectrum activity. IC_50_ values of compound 1 were measured against various viruses in corresponding cell lines. NA, not applicable. IC_50_ values of compound 1 were measured against indicated viruses. Ninety-five percent confidence intervals are shown in parentheses.

Since the cell-to-cell fusion assay employed for HTS involved the cSLAM receptor, we next investigated whether the activity of compound 1 relied on the presence of cSLAM. To this aim, Vero cells stably expressing cSLAM or cNectin-4 (the CDV receptor expressed in epithelial cells [27]) were infected with OP^neon/nLucP^ in the presence of increasing concentrations of compound 1 and relative luciferase activity was measured in each of the cell lines. Note that OP-CDV employs a third unknown receptor (xR) expressed in Vero cells. In all three infected cell lines, the activity of compound 1 remained equally efficient in terms of viral inhibition (Fig. 3A), which indicated that the activity of compound 1 was cSLAM, cNectin-4, and xR independent.

To investigate whether the inhibitory activity of compound 1 was attributable to any virucidal activities, virions (OP^neon/nLucP^) were incubated with either compound 3 or compound 1 at inhibitory concentrations for 1 h at 4°C. The virus-compound mixtures were then diluted 10-fold, thereby reaching noninhibitory concentrations. Hence, if the given compound would efficiently interact with any components of the viral particle, the subsequent dilution step might not interfere with the inhibitory activity. Upon inoculation of the diluted mixtures in Vero-cSLAM cells, the viral infectivity was assessed. While compound 3 still efficiently prevented viral infectivity even after the dilution step, compound 1 entirely lost its inhibitory activity (Fig. 3B). These data strongly suggested that the mode of action of compound 1 was not associated with virucidal activity.

Next, a time-of-addition experiment was performed using OP^neon/nLucP^ to investigate which step of the viral life cycle may potentially be inhibited by compound 1. To this end, Vero-cSLAM cells were treated with compound 1 at the time of infection or at distinct time points postinfection. In this set of experiments, two previously characterized compounds (compound 3 and ERDRP-0519 [Fig. 1A]) targeting two distinct stages of the viral life cycle (entry and replication, respectively) were added as controls. Strikingly, while the profile of compound 1 inhibition differed from the one exerted by compound 3, it acted like ERDRP-0519 (Fig. 3C), when compounds were added after 20 h of infection. Collectively, these findings provided good evidence that the antiviral activity exerted by compound 1 was not mediated at the cell entry level but rather interrupted the viral replication process.

### Compound 1 inhibits the function of the RdRp complex.

Since the viral RdRp complex plays a pivotal role in replicating viral genomes, we tested whether compound 1 could perturb the activity of the RdRp complex. In order to investigate this notion, the bioactivity of compound 1 was determined employing a plasmid-based minigenome system (mREP-OP^neon/nLucP^) in transfected Bsr-T7 cells. In this system, the recorded luciferase activity corresponded to the RdRp complex activity and did not rely on any other viral components. While, in the case of the entry inhibitor 3, the luciferase activity remained similar to that for vehicle (dimethyl sulfoxide [DMSO])-treated controls, 10 μM compound 1 was sufficient to significantly inhibit the luciferase activity (Fig. 3D). Again, the profile of compound 1-mediated viral inhibition appeared to be very similar to the viral polymerase inhibitor ERDRP-0519. Moreover, consistent with data obtained with CDV, inhibition of MeV polymerase activity was recorded, as assessed by employing an MeV-based minigenome assay (Fig. S3). Taken together, this set of experiments further confirmed that compound 1 targeted the replication stage of CDV.

10.1128/mBio.02621-21.3 FIG S3 Compound 1 (ZHAWOC9045) inhibits the activity of MeV polymerase complex. Plasmid-based minigenome luciferase assay to determine the bioactivity of MeV polymerase complex. Relative luminescence values were normalized for values obtained in the presence of DMSO control and represent means ± SD from three independent experiments. Statistical significance of differences was determined using one-way ANOVA followed by Dunnett’s multiple-comparison test (****, *P* < 0.0001; NS, not significant). Download FIG S3, TIF file, 2.2 MB. Copyright © 2021 Shrestha et al. 2021 Shrestha et al. https://creativecommons.org/licenses/by/4.0/ This content is distributed under the terms of the Creative Commons Attribution 4.0 International license.

### Compound 1 inhibits CDV in a host cell-dependent manner.

It remained unclear whether the compound directly targeted the RdRp complex or whether it exerted its inhibitory function via targeting a host factor. In order to interrogate this idea, the bioactivity of compound 1 was determined in cell lines of various species origin against OP^neon/nLucP^. The IC_50_ values of the compounds were measured based on luciferase activity. Interestingly, and despite being tested against the same virus, IC_50_ values of compound 1 varied among different cell lines (Fig. 3E). We noted that the IC_50_ values against OP^neon/nLucP^ in Bsr-T7 cells were about 7 μM, which nicely corresponded with the ∼50% inhibition of polymerase activity recorded in the minigenome assay (generated in Bsr-T7 cells at 10 μM). Overall, the discrepancies in the inhibitory efficacy of compound 1 in a host cell-dependent fashion indicated the possibility that the compound might target a cellular factor promoting viral replication rather than the RdRp directly.

In order to substantiate the possibility that compound 1 targeted the host cell factor, we opted for the direct visualization of the intracellular binding of compound 1 by fluorescently labeling the compound using click chemistry. This technology involves a highly specific and efficient chemical reaction of two binding moieties (alkyne and azide [28]). To this end, compound 1 was tagged with alkyne (termed compound 1-alk [20269], structure shown in Fig. 3G). The cellular localization of 1-alk was next determined by a click reaction with an azide-linked Alexa Fluor 488 molecule. Two irrelevant alkyne-tagged compounds (20264 and 21320) were used as negative controls. Indeed, a clear green fluorescent signal confirmed intracellular localization of 1-alk, whereas no signal was detected for both control molecules (Fig. 3F). This observation was verified in both Vero (monkey) and P114 (canine) cell lines. Of note, we also confirmed that the addition of alkyne moiety on compound 1 did not compromise the inhibitory activity of the compound (Table S5).

10.1128/mBio.02621-21.10 TABLE S5 Full list of all tested compounds with the ZHAWOC code, IC_50_ value, and structure in the quantitative viral inhibition assay based on Vero-cSLAM cells infected with the recombinant CDV OP^neon/nLuc^. Download Table S5, DOCX file, 1.1 MB. Copyright © 2021 Shrestha et al. 2021 Shrestha et al. https://creativecommons.org/licenses/by/4.0/ This content is distributed under the terms of the Creative Commons Attribution 4.0 International license.

### Compound 1 displays broad-spectrum antiviral activity.

We next investigated whether compound 1 displayed a broad-spectrum efficacy. To this end, the inhibitory activity of compound 1 was determined against various related viruses: measles virus (MeV) and parainfluenza virus type 5 (PIV-5) (both from the *Paramyxoviridae* family), respiratory syncytial virus (RSV) (*Pneumoviridae* family), and vesicular stomatitis virus (VSV) (*Rhabdoviridae* family). Susceptible cells were infected with MeV, PIV-5, RSV, and VSV in the presence of increasing concentrations of compound 1 (Table 1). IC_50_ values were determined either by recording luciferase activity (CDV, MeV, PIV-5, VSV) or by plaque reduction assay (RSV). For MeV and CDV, the compound returned IC_50_ values of in the range of 0.2 to 0.7 μM. The IC_50_ values for PIV-5 and RSV were about 7 and 6 μM, respectively (Fig. 3H). Interestingly, compound 1 did not display any activity in the case of VSV. Collectively, the recorded potent inhibitory activity of compound 1 against various paramyxoviruses and pneumoviruses (but not rhabdoviruses) not only illustrated the highly attractive broad-spectrum antiviral activity of the newly discovered compound but also strengthened the notion that the compound 1 may target a host cellular factor necessary for promoting viral replication.

### Compound 1 mitigates rapid emergence of viral resistance.

**FIG 4:**
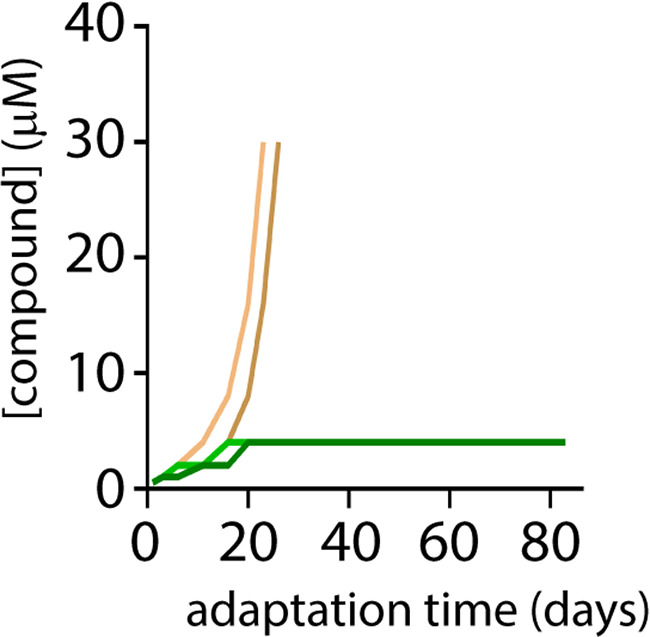
Compound 1 impedes the rapid emergence of viral mutants. Wild-type A75/17-CDV was continuously adapted for 83 days in the presence of either ERDRP-0519 (virus directed) or compound 1 (host directed). Four independent adaptations were followed for compound 1 (green), whereas two independent adaptations were followed for ERDRP-0519 (brown).

Host-directed antiviral strategy may not only provide molecules with broad-spectrum activity but may additionally strongly reduce the chances of generation of drug-resistant viral variants (23). Having demonstrated the former quality, we investigated the latter by performing a stepwise viral growth adaptation in the presence of compound 1. In this set of experiments, the previously described MeV polymerase-directed inhibitor, ERDRP-0519 (15), was included as control. The wild-type A75/17-CDV strain (A75^neon^) was employed as the input virus for the adaptation. In order to let the virus adapt to the compound gradually, compound concentrations were doubled only once the virus-induced cytopathic effect (CPE) became pronounced in the cell monolayer. Strikingly, while rapid resistance to ERDRP-0519 emerged in approximately 20 to 25 days (tolerated dose at the end of adaptation was ≥30 μM), compound 1 did not induce any detectable viral resistance (in four separate replicates) until the end of the 83-day study (Fig. 4). Collectively, these data not only supported the idea that compound 1 targeted a host cellular factor but also illustrated that, in contrast to the virus-directed ERDRP-0519 inhibitor, our newly discovered molecule exhibits a high genetic resistance barrier.

### Chemical optimization of compound 1.

The variation of the molecule was divided in three parts: the phenol component, the glycolic acid linker, and the thiazole component. We diversified them to get better insights into the role of the fragments and then combined the most promising building blocks. The glycolic acid linker allows a convenient two-step synthesis with last-step derivatization to change the flanking phenols and thiazoles.

Figure 2 illustrates the two generic synthetic routes toward compound 1 and derivatives thereof. Either we formed the phenolic ether first, followed by an amide formation, or vice versa. The complete list of the final compounds with modified thiazole moieties is shown in Fig. S5, scheme 1, and with modified phenol moieties in Fig. S5, scheme 2. Most of the used phenol and 2-aminothiazole building blocks were commercially available. Some phenol building blocks were synthesized starting with pyrogallol (Fig. S5, scheme 3). We also synthesized specific 2-aminothiazole building blocks to establish our SAR (Fig. S5, scheme 4).

10.1128/mBio.02621-21.5 FIG S5 (Scheme 1) Synthesis of derivatives of compound 1 with different thiazole moieties. (A) Phenol derivative (1 eq) in DCM with trimethylamine (1.2 eq) and chloroacetyl chloride (1.2 eq) at 0°C to RT for 1 h. (B) Aromatic amine (1.1 eq) in DMF with Cs_2_CO_3_ (1.5 eq), chloroalkyl derivative (1.0 eq), and NaI (0.1 eq), at 60°C overnight. (C) 9045 (1 eq), KOH aq., benzylbromide, methyliodide, or allyl bromide (1 eq) in DMF at RT for 20 h. (Scheme 2) Synthesis of derivatives of compound 1 with different phenol moieties. (A) Aromatic amine (1 eq) in DCM with trimethylamine (1.2 eq) and chloroacetyl chloride (1.2 eq) at 0°C to RT for 1 h. (B) Aromatic amine or alcohol (1.1 eq) in DMF with Cs_2_CO_3_ (1.5 eq), chloroalkyl derivative (1.0 eq), and NaI (0.1 eq) at 60°C overnight. (Scheme 3) Synthesis of acetals from pyrogallol. (A) Pyrogallol (1 eq) and 2,2-dimethoxypropane (1.3 eq) in toluene reflux for 6 h. (B) Ketone (1.5 eq), pyrogallol (1 eq), and *p*-toluenesulfonic acid in toluene at reflux for 6 h. (Scheme 4) Synthesis of thiazole and imidazole building blocks. (A) 2-Aminothiazole derivative (1 eq), Cs_2_CO_3_ (1.6 eq), 1-chloro-4-pentyne (1 eq), and NaI (0.1 eq) in DMF at 70°C overnight. (B) 2-Amino-4-methyl-thiazol (1 eq) and *N*-chloro-succinimide (1 eq) in DMF at 0°C to RT for 6 h. (C) 2-Aminobenzimidazole (1 eq), Cu_2_O (0.1 eq), KOH (2 eq), and iodobenzene (1.5 eq) in DMSO at 120°C for 14 h. (Scheme 5) Synthesis of derivatives of compound 1 with various linkers. (A) 2,3-Dihydro-2,2-dimethyl-7-hydroxybenzofuran (1 eq), halocarboxylic acid (1.2 eq), and NaOH (2.3 eq) in water at 100°C for 45 min. (B) Carboxylic acid (1.0 eq), amine (1.3 eq.), DIPEA (3.0 eq), and HATU (1.1 eq) in DMF at 0°C to RT for 1 h. (C) 2,3-Dihydro-2,2-dimethyl-7-hydroxybenzofuran (1 eq), tetrabutylammonium iodide (TBAI) (0.02 eq), NaOH aq., and dichloroethane (100 eq) at 85°C for 15 h. (D) Aromatic amine or alcohol (1.1 eq) in DMF with Cs_2_CO_3_ (1.5 eq), chloroalkyl derivative (1.0 eq), and NaI (0.1 eq) at 60°C overnight. (E) *N,N′*-Diisopropylcarbodiimide (DIC) (1.3 eq), carboxylic acid (1 eq), and 2-hydroxybenzothiazole (1 eq) in DCM at RT for 3 h. (F) Amide (1 eq) and Lawesson’s reagent (0.6 eq) in toluene at 110°C for 3 h. (G) 2,3-Dihydro-2,2-dimethyl-7-hydroxybenzofuran (1 eq), 3-bromo-prop-1-yne (1.1 eq), and K_2_CO_3_ (2.5 eq) in acetone at reflux for 4 h. (H) Alkyne (1 eq), NaN_3_ (1.2 eq), 2-chlorobenzothiazole (1 eq), CuI (0.1 eq.), and *N,N*-dimethylethylenediamine (0.15 eq) in DMF at 40°C for 2 h. (I) Aromatic amine or alcohol (1 eq) in DCM with trimethylamine (1.2 eq) and chloroacetyl chloride (1.2 eq) at 0°C to RT for 1 h. (J) Aromatic amine or alcohol (1.1 eq) in DMF with Cs_2_CO_3_ (1.5 eq), chloroalkyl derivative (1.0 eq), and NaI (0.1 eq) at 60°C overnight. (Scheme 6) List of purchased substances for hit expansion of 9045 from Life Chemicals, Enamine, and ChemDiv. These compounds were all not active. Download FIG S5, DOCX file, 0.5 MB. Copyright © 2021 Shrestha et al. 2021 Shrestha et al. https://creativecommons.org/licenses/by/4.0/ This content is distributed under the terms of the Creative Commons Attribution 4.0 International license.

The glycolic acid linker was replaced with different functionalized linkers to look for tolerated alternatives and to develop the SAR (Fig. S5, scheme 5). We synthesized derivatives with alkyl groups at the α-position on the glycolic acid moiety and extended linkers to explore possible attachment points. To find alternatives to the amide bond in compound 1, we replaced it with ether, amine, ester, thioamide, and triazole. The ether bond in compound 1 was replaced by an ester and an amide next to the phenol moiety. We also synthesized derivatives with an additional ring system between the α-position of the glycolic acid and the phenol fragment to investigate whether the rigidified ligand has a higher activity. All IC_50_ values in this section were measured using a quantitative viral inhibition assay based on Vero-cSLAM cells infected with the recombinant CDV OP^neon/nLuc^. Luciferase activity was recorded 24 h postinfection.

### Structure-activity relationship (SAR) of the phenol moiety.

The electron-rich phenol moiety was exchanged with different phenols and anilines. The full list of the compounds is shown in Table S1. Only fragments with larger substitutions in *ortho* or fused five-ring systems in *ortho* to *meta* position with respect to the linking ether or amine group were tolerated. The active molecules pose an electron-rich aromatic system like indoles or phenyls with at least another ether substituent. The indole fragments favor the amine linkage (compound 5, IC_50_ = 0.73 μM, versus compound 6, IC_50_ = 8.3 μM) while the 2,3-dihydrobenzofuran building block shows better inhibition in combination with the ether linkage (compound 1, IC_50_ = 0.52 μM, versus compound 7, IC_50_ = 1.7 μM). Inhibitory activity was achieved with pyrogallol-based acetals. The cyclopentyl acetal 8 and the isopropyl acetal 9 show the same activity as compound 1, and an improvement was achieved with the heptyl-2-acetal derivative 10. This derivative was not further pursued due to its lipophilicity and the moderate stability of acetals.

10.1128/mBio.02621-21.6 TABLE S1 Shown is a list of derivatives of compound 1 with different groups on the phenol moiety. The IC_50_ values were measured in the quantitative viral inhibition assay based on Vero-cSLAM cells infected with the recombinant CDV OP^neon/nLuc^. Download Table S1, DOCX file, 0.4 MB. Copyright © 2021 Shrestha et al. 2021 Shrestha et al. https://creativecommons.org/licenses/by/4.0/ This content is distributed under the terms of the Creative Commons Attribution 4.0 International license.

### SAR of the benzothiazole moiety.

The screening hit 1 has an unsubstituted benzothiazole bound to the amide. Substitutions on the phenyl ring of the benzothiazole were not well accepted (results shown in Table S2). Already, the introduction of a methyl group in the position 4 or a fluorine in the position 6 leads to an activity decrease. Larger substituents lead to an even larger loss of activity. In contrast to the activity loss of the benzothiazoles, some alkyl-substituted thiazoles showed a significant improvement (all results shown in Table S2). The unsubstituted thiazole 11 does not have a strong inhibitory effect. A methyl group on the position 4 in compound 12 improves the IC_50_ to 2.2 μM and in position 5 in compound 13 improves it to 0.86 μM. The thiazole 14 (IC_50_ = 0.13 μM) with two vicinal methyl groups and compound 15 results in improved inhibitory activity compared to compound 1. The highest inhibitory activity in this series was found with the saturated ring systems in compounds 16 (0.084 μM) and 17 (0.056 μM), consequently. We also tested a wide variety of alkyl amines and anilines, and none of them showed an activity at 100 μM (structures shown in Fig. S5, scheme 6).

10.1128/mBio.02621-21.7 TABLE S2 Shown is a list of derivatives of compound 1 with different groups on the amide position including 2-aminobenzothiazoles and 2-aminothiazoles. The IC_50_ values were measured in the quantitative viral inhibition assay based on Vero-cSLAM cells infected with the recombinant CDV OP^neon/nLuc^. Download Table S2, DOCX file, 0.7 MB. Copyright © 2021 Shrestha et al. 2021 Shrestha et al. https://creativecommons.org/licenses/by/4.0/ This content is distributed under the terms of the Creative Commons Attribution 4.0 International license.

**FIG 5:**
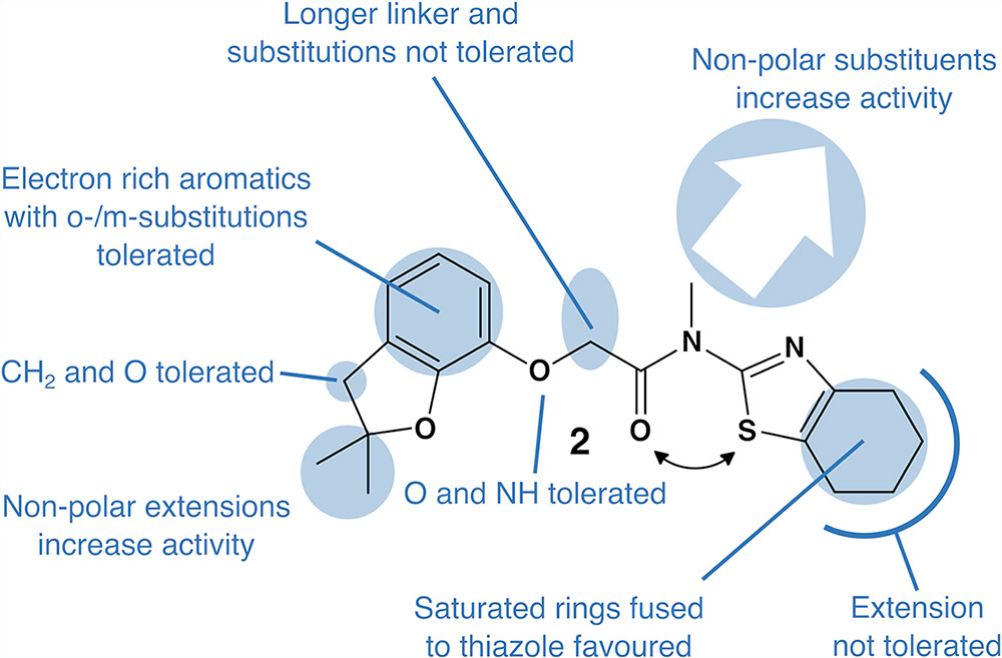
Structure of the optimized inhibitor 2 (ZHAWOC21026) depicting the SAR conclusions. The black arrow visualizes the possible C=O···S interaction (50).

We replaced the benzothiazole with other aromatic heterocycles but could not identify active alternatives (full list in Table S3). The benzothiophene derivative 18 showed no activity at 100 μM, and the benzoxazole 19 and benzimidazole 20 have an IC_50_ higher than 40 μM. The nitrogen seems to play an important role either as an interaction partner for binding to the target or for the electron density in the aromatic system. The loss of the sulfur-to-oxygen interaction (as shown in Fig. 5) might explain the high-affinity loss best. Benzimidazoles have a hydrogen bond donor. This would also enable an intramolecular O···N interaction like the O···S contact (in Fig. 5) but leads to an overall higher polarity. Only benzimidazoles with nonpolar substitutions on the 1-position as in compound 21 remained active. This indicates a nonpolar binding site in this orientation. Other aromatic five-membered rings show less or no activity (Table S3).

10.1128/mBio.02621-21.8 TABLE S3 Shown is a list of derivatives of compound 1 with different groups on the amide position including different aromatic heterocycles and tertiary amines. The IC_50_ values were measured in the quantitative viral inhibition assay based on Vero-cSLAM cells infected with the recombinant CDV OP^neon/nLuc^. Download Table S3, DOCX file, 0.5 MB. Copyright © 2021 Shrestha et al. 2021 Shrestha et al. https://creativecommons.org/licenses/by/4.0/ This content is distributed under the terms of the Creative Commons Attribution 4.0 International license.

A significant improvement of the IC_50_ was observed with extensions on the amide nitrogen (Table S3). These tertiary amides were significantly more potent. A methyl group in compound 22 boosts the activity 3-fold. The hydrogen bond-donating property of the amide NH is therefore not necessary for the target binding. Larger substituents like the pentynyl in compound 23 or benzyl group in compound 24 are also well tolerated. This confirms free space in this orientation and opens up possibilities to attach functional moieties for binding studies. The activity improvements may also be explained with better membrane permeability due to the reduced polarity of the compound. The most active molecules resulted from the combination of the improved thiazole fragments with amide nitrogen alkylations. The saturated fused thiazole derivatives compounds 2 (ZHAWOC21026) and 25 (ZHAWOC21048) with an amide N-methylation have a much higher inhibitory activity than the according benzothiazole 22. The lowest IC_50_ was fond with *N*-allyl in compounds 26 (ZHAWOC21027) and 27 (ZHAWOC20926) with 4.1 and 8.2 nM, respectively.

In summary, the thiazole moiety seems to be very important and small modifications lead to significant decreases in antiviral activity. The change from benzothiazole to the cyclopentyl and cyclohexyl fused thiazoles and the extensions on the amide nitrogen were beneficial for antiviral activity.

### Linker moiety.

We exchanged the glycolic acid linker with linkers of different lengths and functional groups (Table S4). Employing the glycine-like linker in compound 30 was well tolerated with a similar IC_50_ of 0.48 μM. The glycine linker in combination with an N-methylation and the optimized thiazole fragment in compound 31 was significantly more active. A methyl or butyl substituent on the α-position of the glycolic acid in compounds 32 and 33 leads to an over-100-fold activity decrease. Sterically, hindrance or clashes may explain this drop. The longer linker in compound 6 and linkers with ether 34, ester 35, triazole 36, thioamide 37, and amine 38 on the amide position are not tolerated (full list in Table S5).

10.1128/mBio.02621-21.9 TABLE S4 Shown is a list of derivatives of compound 1 with different linkers. The IC_50_ values were measured in the quantitative viral inhibition assay based on Vero-cSLAM cells infected with the recombinant CDV OP^neon/nLuc^. Download Table S4, DOCX file, 0.5 MB. Copyright © 2021 Shrestha et al. 2021 Shrestha et al. https://creativecommons.org/licenses/by/4.0/ This content is distributed under the terms of the Creative Commons Attribution 4.0 International license.

In Fig. 5, we summarize the overall results of our extensive derivatization process leading to detailed SAR understanding.

### Compound 2: a highly potent paramyxovirus and pneumovirus inhibitor.

**FIG 6:**
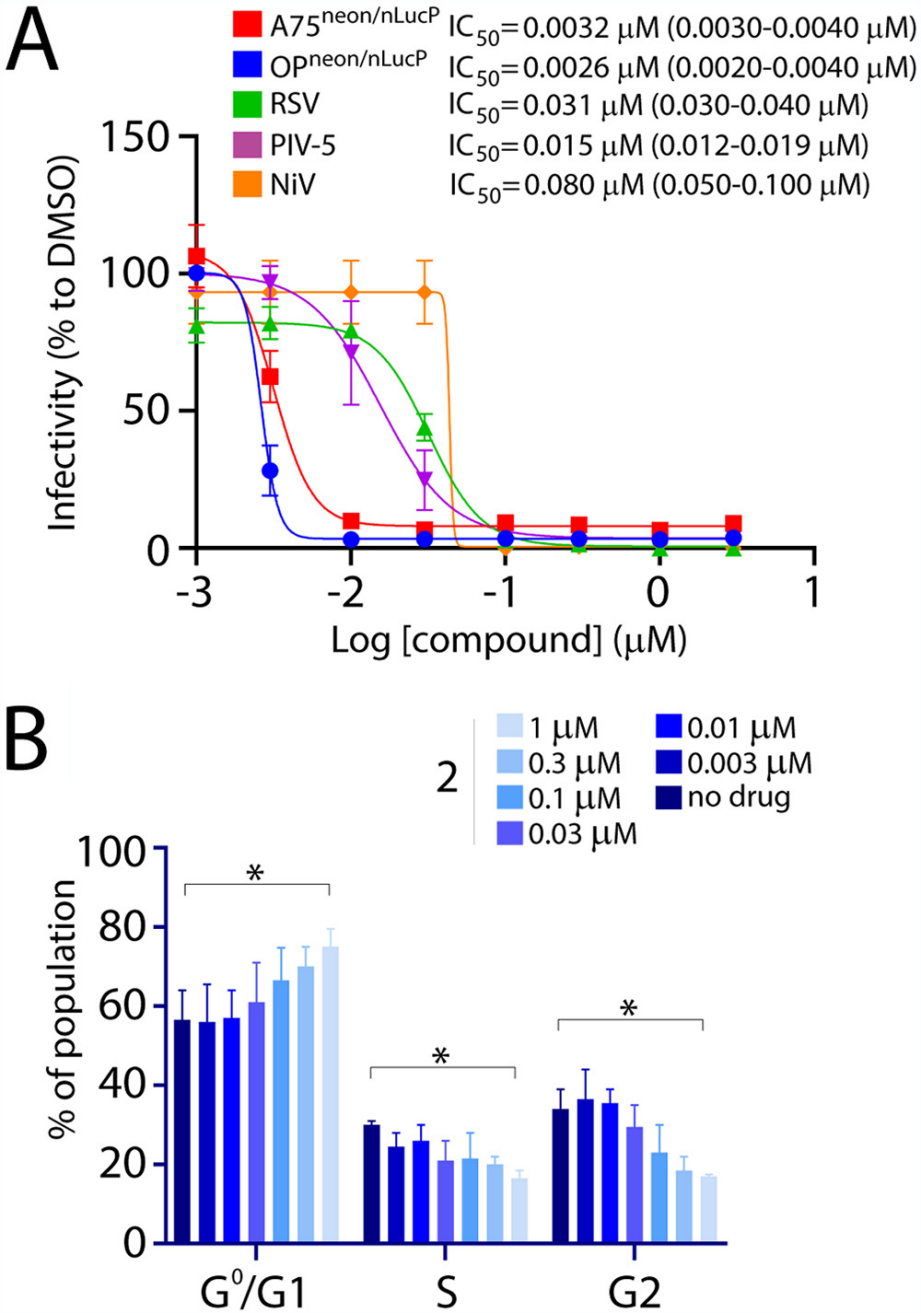
Generation of highly potent variants of compound 1. (A) IC_50_ values of one of the 3 best variants, compound 2, were measured against different members of families *Paramyxoviridae* and *Pneumoviridae* in corresponding cell lines. Ninety-five percent confidence intervals are shown in parentheses. (B) Impact of compound 2 on cell cycle progression of treated cells. Vero cells were incubated in the absence or presence of increasing concentrations of compound 2 for 42 h and analyzed using flow cytometry. The values indicate the means ± SD from three independent experiments. Dunnett’s multiple-comparison test was applied after two-way ANOVA (*, *P* < 0.05).

Overall, our comprehensive SAR studies spotlighted three improved and highly potent derivatives (compounds 2, 26, and 27), characterized by single-digit nanomolar activities. Because those studies were based on infections performed with OP-CDV, it was of high importance to determine whether or not the inhibitory activities of these compounds also extended toward additional viruses. To that end, we selected compound 2 and tested its inhibitory activity against A75/17-CDV, PIV-5, RSV, and NiV in corresponding cells. Strikingly, similarly to OP-CDV, the inhibitory activity of the compound improved by about 100 times against all other tested members of the family *Paramyxoviridae* and *Pneumoviridae* (Fig. 6A). Interestingly, CC_50_ values of compound 2 in all tested cell lines remained similar, which in turn drastically improved the SI values of the compound (Table 1). Moreover, the cell cycle proliferation was abrogated significantly only at concentrations as high as 1 μM (in Vero-cSLAM cells), a concentration about 200 times greater than the inhibitory concentration (Fig. 6B). Note that this ratio was substantially improved compared to the one calculated for our initial compound (of about 60 times for compound 1). Collectively, these findings highlighted the high potential of the newly designed compounds as potent paramyxo- and pneumovirus inhibitors.

## DISCUSSION

Every year, myriad infections caused by paramyxo- and pneumoviruses such as MeV, CDV, and RSV bring a huge disease burden worldwide. Additionally, a highly pathogenic virus like NiV is a particular concern as a potential source of human pandemics. While potent inhibitors indeed have been identified against members of the *Paramyxoviridae* (15–18), the success of developing virus-directed antivirals in general has been invariably challenged by the rapid generation of drug-resistant viruses (29–34). A novel strategy of development of antivirals, by targeting rather a host factor, has been widely explored (35–38). Many compounds identified in such a manner are in the preclinical stages of development, with a few compounds even approved by the FDA (23, 39).

In this study, compound 1 was identified using an HTS performed earlier to discover entry inhibitors against CDV (24). Interestingly, compound 1 clustered with small-molecule compounds, potentially enhancing fusion activity. When investigated in the context of quantitative cell-to-cell fusion assay, compound 1 was indeed confirmed to enhance fusion activity, although membrane fusion increased exclusively at high concentrations (see Fig. S4 in the supplemental material). It is assumed that small molecules at high concentrations may lead to aggregation effects or nonspecific binding. We thus speculate that compound 1, at high concentrations, might have destabilized the CDV prefusion F state and resulted in a moderate acceleration of fusion activity. Alternatively, compound aggregates might have disturbed the cellular lipid membranes (including the plasma membrane), which possibly resulted in accelerated virus-induced fusogenicity. Importantly, the dose-response analysis, in the context of viral infection, highlighted the fact that hit compound 1 strongly inhibited the viral replication at concentrations as low as 1 μM.

10.1128/mBio.02621-21.4 FIG S4 Putative binding of compound 1 with the F-protein and derived impact on activity. Measurement of membrane fusion between effector and target cells in presence of increasing concentration of either compound 3 or 1. Means ± SD of data from three independent experiments are shown. Download FIG S4, TIF file, 3.0 MB. Copyright © 2021 Shrestha et al. 2021 Shrestha et al. https://creativecommons.org/licenses/by/4.0/ This content is distributed under the terms of the Creative Commons Attribution 4.0 International license.

The compound 1 was discovered to act in a host-directed fashion. Host-based interventions inevitably offer advantages over conventional virus-directed antivirals, such as a higher barrier to drug resistance (23) due to lower genetic variability of host factors compared with the mutation-prone nature of viral components. Indeed, no resistance occurred with compound 1 when adapted for the period of 83 days (Fig. 4). While given experiments indicated that the compound targets a host factor, the complete identification of the host factor targeted by this class, however, remains unknown. Undoubtedly, an approach of targeting host factors for antiviral therapy compared to conventional virus-directed agents may carry a burden of cytotoxicity and undesirable drug-induced side effects. When tested in various cell lines, compound 1 appeared to have very low cytotoxicity and only minor impact on the cell cycle progression. Noteworthy is the possibility of minimizing a problem of side effects by orienting the inhibition with precision. In this regard, the application of a host-directed antiviral strategy appears to be more suitable for the inhibition of infections by pathogens predominantly associated with severe acute disease, such as those mediated by most members of the family *Paramyxoviridae*. Indeed, the treatment duration would likely be limited, and therefore, possible drug-related side effects might be reduced.

Often, related viruses use common pathways for their replication in the course of their life cycle, and therefore, a host factor-directed molecule offers a benefit of broad target range. Indeed, compound 1 can very efficiently inhibit members of the family *Paramyxoviridae* and even extends to members of the family *Pneumoviridae* (Table 1), but not the members of the family *Rhabdoviridae*. This also highlights the commonality of host factor usage among the related families of viruses. Interestingly, the compound 1 was codiscovered and hence patented by a company named Retrovirox (40). However, the company identified this compound against primate lentiviruses, which are not the immediate relatives of paramyxo-/pneumoviruses. In the published patent, the mode of action has been described to be via inhibition of virus-mediated downregulation of major histocompatibility complex class I (MHC-I) molecules. Lack of additional publications prevented any interpretations of their findings with regard to our mechanistic studies. Overall, (i) the variability in IC_50_ values depending on the host cell used, (ii) the direct intracellular localization of the compound in uninfected cells, (iii) the pan-paramyxoviral/pneumoviral antiviral activity, and (iv) the lack of rapid emergence of resistant viruses even upon prolonged incubation with the compound strongly supported the notion that compound 1 acts in a host-directed fashion.

In an effort to improve inhibitory efficiency of compound 1, we initiated a SAR study of this compound. To this aim, we synthesized over 100 compounds and tested them in the virus inhibition assay to develop a detailed understanding of the SAR. The set of highly active compounds is quite narrow, and most parts of the hit molecule barely tolerate any modifications. By modifying the benzothiazole fragment and alkylations of the amide nitrogen, we eventually invented very interesting single-digit nanomolar inhibitors. Fortunately, the cytotoxicity (around 50 μM, depending on cell lines) was not changed by these modifications, and the selectivity index consequently increased to remarkable values (SI of 1,000 to 18,000) depending on the virus or cell line tested.

Altogether, we present a novel, host-directed small-molecule antiviral class (e.g., compound 2) with very high potency but low cytotoxicity. The compound inhibits significant members of the families *Paramyxoviridae* and *Pneumoviridae*, including MeV, CDV, NiV, PIV-5, and RSV. This class of compounds inhibits the replication via the RdRp complex but, however in a host-directed manner, which in turn prohibits the emergence of resistant mutants. Further exploration of the compound’s efficacy *in vivo* as well as explicit identification of the host target might present these compounds as promising candidates for therapeutic usage in the future.

## MATERIALS AND METHODS

### Cell culture and transfection.

Vero cells (ATCC CCL1-81), Vero cells stably expressing canine SLAM (Vero-cSLAM, kindly provided by Yusuke Yanagi, Kyushu University, Fukuoka, Japan), Vero-cSLAM-Green Fluorescent Protein (GFP)/LgBiT, Vero-Fs-sH-Red Fluorescent Protein (RFP)/HiBiT, HEp-2 cells (ATCC, CCL-23) baby hamster kidney cells stably expressing T7 polymerase (Bsr-T7/5) (41), and canine mammary anaplastic cancer P114 (kindly provided by Elpetra Timmermans-Sprang, University of Utrecht, Utrecht, Netherlands) (42) were maintained in Dulbecco’s modified Eagle’s medium (DMEM; Gibco, Invitrogen, Carlsbad, CA, USA) containing 10% fetal calf serum (FCS; BioSwissTech, Schaffhausen, Switzerland) and penicillin-streptomycin (pen/strep) at 37°C and 5% CO_2_ (excluding Vero-Fs-sH-RFP/HiBiT cells, which were cultured in the presence of 10 μM asunaprevir). All the transfections were done using Trans-It reagent according to the manufacturer’s instruction (Mirus). Stable transfection of Vero-cSLAM-Green Fluorescent Protein (GFP)/LgBiT and Vero-Fs-sH-Red Fluorescent Protein (RFP)/HiBiT has been described previously (24).

### Measles virus (MeV) rescue.

The recombinant MeV Moraten vaccinal strain, expressing firefly luciferase, was generated using pB(+)Mor-Luc plasmid, produced by introducing the firefly luciferase gene sequence into a new transcription unit located between the P and M genes in the pB(+)MVvac2 plasmid, kindly provided by Roberto Cattaneo (Mayo Clinic, USA). The recombinant MeV IC323 strain, expressing firefly luciferase, was generated by engineering pB(+)IC323-EGFP plasmid, kindly provided by Yusuke Yanagi (Kyushu University, Japan). Briefly, the enhanced GFP (EGFP) gene was excised and the firefly luciferase gene sequence was introduced into a new transcription unit located between the H and L genes. Recombinant Moraten and IC323-luciferase were rescued in 293-3-46 cells as previously described (43). Viral strains were produced and titrated on Vero/hSlam cells.

### Parainfluenza virus type 5 (PIV-5) rescue.

A plasmid containing the full-length genome of PIV-5 strain W3A (termed pBH276; kindly provided by Biao He, Department of Infectious Diseases, University of Georgia College of Veterinary Medicine, USA) was modified in two steps. First, a T7 promoter, an Hhrbz sequence, the PIV-5 W3A leader sequence, and an mNeonGreen gene were inserted to the N terminus of the N gene together with a P2A motif (commercially constructed in one fragment; Eurofins Genomics Germany GmbH) for translational separation. Then, the nLucP gene (nanoluciferase gene fused to the “pest” degradation motif; Promega) was cloned as an additional transcription unit between the P and M genes. The obtained plasmid was designated PIV-5^neon/nLucP^. Recombinant PIV-5^neon/nLucP^ was rescued in Vero cells as previously described (24).

### Virus inhibition assay.

OP^neon/nLucP^ (unpublished data), wild-type CDV strain (A75/17^neon/nLucP^) (44), MeV-Edm, MeV-ICB (mentioned above), RSV-GFP (45), rNiV-EGFP (46), and PIV-5^neon/nLucP^ (mentioned above) have been rescued as described previously. Desired inhibitors, dissolved in DMSO, were added in a 96-well plate (Greiner Bio-One) starting at 100 nM with increasing concentration of half a log up to 100 μM. Desired virus was then added to the plates at a multiplicity of infection (MOI) of 0.04 (OP^neon/nLucP^, A75/17^neon/nLucP^, MeV-Edm, MeV-ICB, RSV, rNiV-EGFP) or 1 (PIV-5) and incubated at 4°C for 1 h. Mixtures were then added to Vero-cSLAM cells (OP^neon/nLucP^, A75/17^neon/nLucP^, MeV-Edm, MeV-ICB, RSV, rNiV-EGFP) or Vero cells (PIV-5) preseeded in a separate 96-well plate and incubated at 37°C for 24 h. The luminescence was measured using the Nano-Glo live cell assay system (Promega) and a multiplate reader (Cytation 5; BioTek, Winooski, VT, USA).

For RSV, due to the lack of luciferase reporter, plaques were counted. In order to do that, after 48 h of infection, the cells were fixed and incubated with a biotinylated anti-RSV antibody (Bio-Rad) for 1 h, followed by 30 min of incubation with ExtrAvidin peroxidase (Sigma) and staining with the 3,3′-diaminobenzidine substrate (Sigma). The average plaque count of four replicates of RSV-GFP (originally generated by Mark Peeples, Nationwide Children’s Hospital, Columbus, OH, USA [47])-infected HEp-2 cells at 1,000 PFU was taken as 0% inhibition.

Nipah virus was amplified and titrated in Vero E6 cells. PGSA745-EFNB2 cells (kindly provided by B. Lee) were cultured in DMEM–F-12 supplemented with nonessential amino acids (NEAA) and were seeded in 96-well plates. The following day, subconfluent cells were infected with 100 PFU of rNiV-EGFP. After 1 h, cells were treated with antiviral compound in order to obtain expected final concentrations. Controls were treated with a corresponding amount of vehicle (i.e., DMSO). After 24 or 48 h, RNA was extracted from supernatant fluids and reverse transcribed. Obtained cDNA was used to quantify NiV N (using historical primers; For, GGCAGGATTCTTCGCAACCATC; Rev, GGCTCTTGGGCCAATTTCTCTG) using Platinum SYBR green qPCR SuperMix-UDG on a StepOnePlus real-time PCR system. Results were obtained from 3 separate experiments.

MeV and NiV infections were carried out at the CIRI in a biosafety level 2 (BSL2) laboratory (Lyon) and at the INSERM Jean Mérieux BSL4 laboratory in Lyon, France.

Cell viability was determined using either the MT cell viability kit (RealTime-Glo MT cell viability assay; Promega) or alamarBlue. After the measurement, all statistical analysis was carried out using the GraphPad Prism 8 package. IC_50_ and CC_50_ values were calculated from dose-response data sets through 4-parameter variable slope regression modeling; values are expressed with 95% confidence intervals (CIs).

### Virucidal effect.

OP^neon/nLucP^ (MOI of 4) was added in a plate containing or not each compound and incubated for 60 min at 4°C. Afterward, the virus and compound mixture was diluted to a noninhibitory drug concentration and added to a monolayer of Vero-cSLAM cells. After 24 h, luminescence was measured using the Nano-Glo live cell assay system (Promega).

### Time-of-addition assay.

Vero-cSLAM cells (96-well plate format) were infected with OP^neon/nLucP^ at an MOI of 0.04, in the presence of compound 3G (final concentration, 30 μM), ERDRP-0519 (final concentration, 10 μM), or ZHAWO9045 (final concentration 10 μM) added at the indicated time points. Control cells were infected in the presence of equal amounts of DMSO. At 48 h postinfection, luminescence was measured using the Nano-Glo live cell assay system (Promega).

### Minireplicon transient expression assay.

A cDNA-based OP-CDV (AF305419.1) minigenome was synthetically synthesized (referred to as mREP^neon/nLucP^). Briefly, the minigenome consists of the following sequence arrangements (from 5′ to 3′): T7 promoter, hammerhead (HH) ribozyme, leader, gene transcription start, gene expression cassette (expressing both the mNeonGreen and nLuc reporter proteins separated by a P2A sequence), gene transcription stop, trailer, T7 terminator, and the hepatitis delta ribozyme (HDV) sequence. In parallel, the OP-CDV sequence of the N, P, and L genes was cloned into the T7-driven pTM expression vector. Note that (i) the C protein was knocked out from the pTM-P vector (pTM-PCko) and (ii) the highly conserved glutamic acid at position 13 of the L protein had to be repaired (L-V13E) to recover functionality. Finally, for detection purposes, the L protein was additionally tagged with the hemagglutinin (HA) sequence at the N-terminal region (HA-L-V13E [unpublished data]). For the CDV-based replication assays, Bsr-T7/5 cells were transfected with plasmid DNAs encoding CDV-OP-minigenome mREP^neon/nLucP^ (0.8 μg), pTM CDV(OP)-N (0.3 μg), or pTM CDV(OP)-P/Cko (0.3 μg) and pTM CDV(OP)-HA-L-V13E (0.1 μg) (unpublished data) in the presence of compounds. After 48 h of transfection, the luminescence was measured using the Nano-Glo live cell assay system (Promega) and a multiplate reader (Cytation 5; BioTek, Winooski, VT, USA). For MeV, the minireplicon transfection was performed as described previously (48). As a negative control, compound 3 (entry inhibitor, 50 μM) and, as a positive control, replication inhibitor (ERDRP-0519, 10 μM) were taken.

### Click chemistry.

Cells were seeded in a 24-well plate containing glass coverslips. Alkyne-tagged compounds were added at the concentration of 2 μM and incubated at 37°C for 3 h. Afterward, cells were washed, fixed with 4% paraformaldehyde for 15 min, permeabilized with 0.2% Triton X-100 for 5 min, and blocked with 2% bovine serum albumin for 15 min. The click reaction was then performed with the Click-iT cell reaction buffer kit (Invitrogen) according to the manufacturer’s instructions for 1 h. The cells were washed, stained with 4′,6-diamidino-2-phenylindole (DAPI), and analyzed by confocal microscopy (Olympus, Japan).

### Virus adaptation.

Vero-SLAM cells were infected with A75/17-CDV (A75^green^) at an MOI of 0.01 and incubated in the presence of gradually increasing concentration of ZHAWOC9045 starting at 0.5 μM. Simultaneously, the virus polymerase inhibitor ERDRP-0519 was examined in parallel. As soon as extensive cell-to-cell fusion was visualized, cell-associated viral particles were extracted via freeze-thaw cycles, diluted 10-fold, and inoculated in a fresh cell monolayer in the presence of compound at unchanged or doubled concentrations. When cultures became overconfluent, cells were reseeded for continued incubation in the presence of the same compound concentration as before. Cultures treated with the highest compound concentrations in which virus-induced cytopathicity became detectable were used for further adaptation. The adaptation was halted after 83 days of continued incubation or, in the case of ERDRP-0519, when virus-induced cytopathicity was readily detectable in the presence of 30 μM compound in accordance with previous results.

### Synthetic chemistry.

All final compounds were obtained with >95% purity.
