# Supplementary figures and images for: Highly Potent Host-Specific Small-Molecule Inhibitor of Paramyxovirus and Pneumovirus Replication with High Resistance Barrier
Source: mBio. 2021 Nov 2;12(6):e02621-21. doi: 10.1128/mBio.02621-21 (PMC8561388; doi:10.1128/mBio.02621-21)

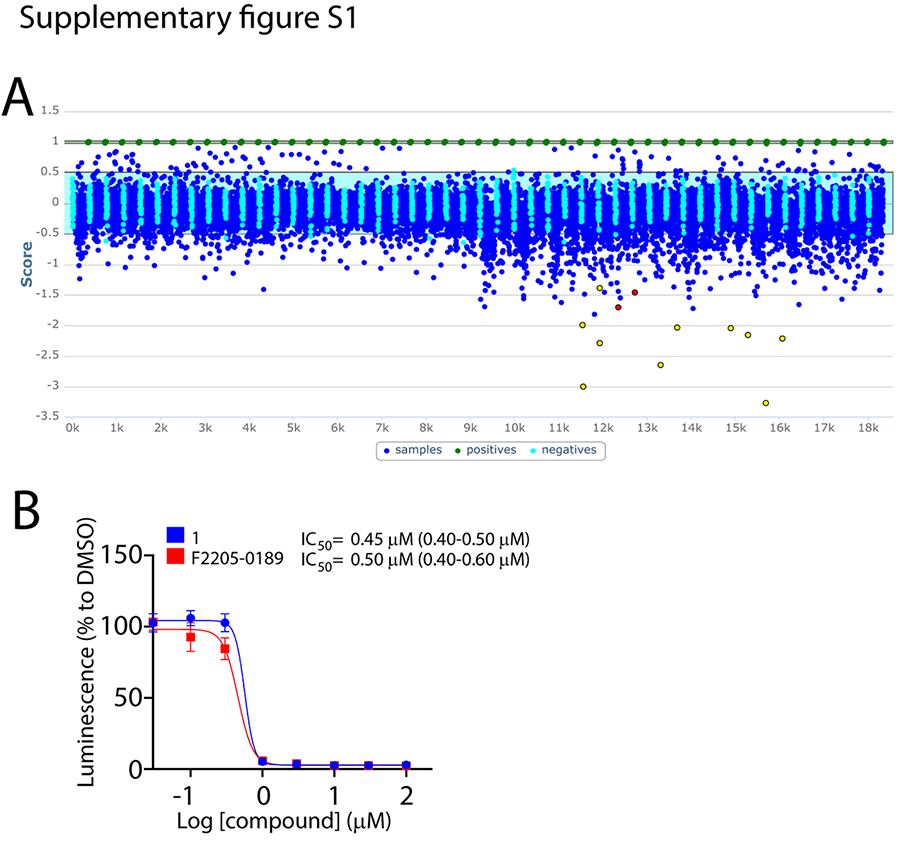

Supplement: FIG S1 [file mbio.02621-21-sf001.tif]

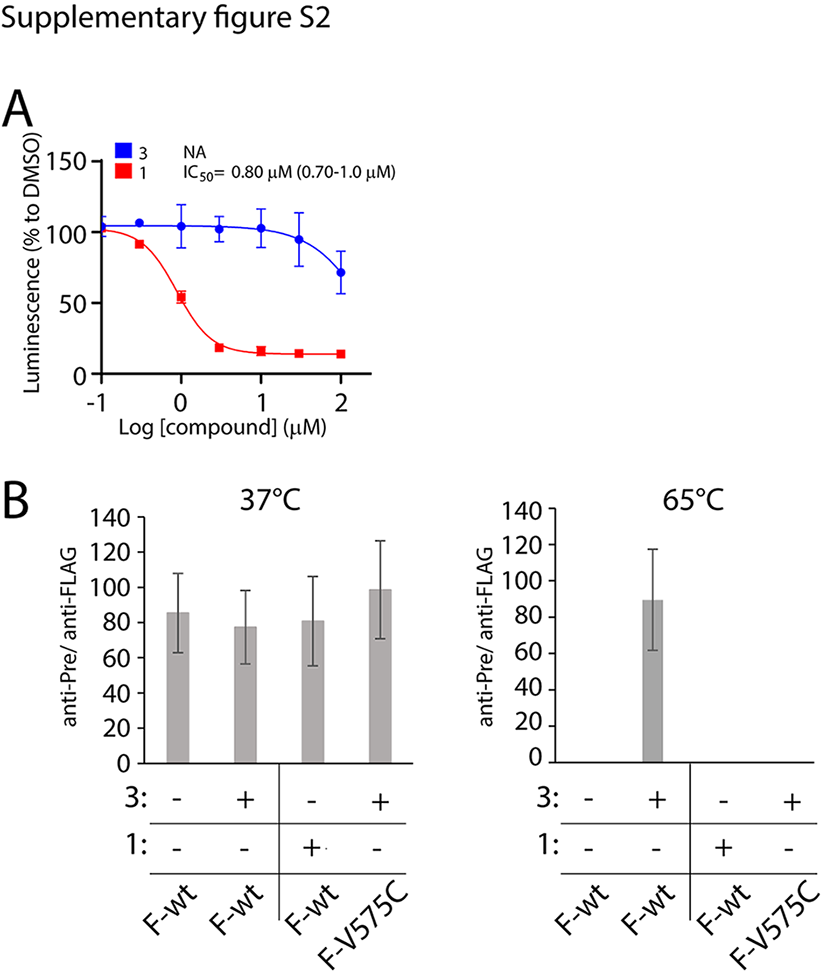

Supplement: FIG S2 [file mbio.02621-21-sf002.tif]

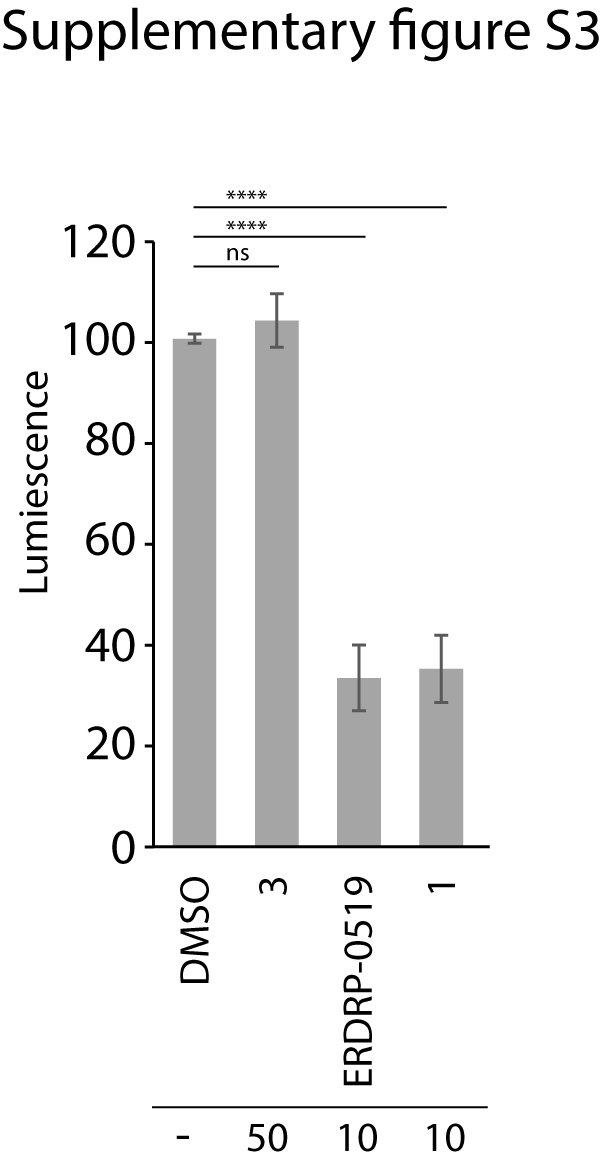

Supplement: FIG S3 [file mbio.02621-21-sf003.tif]

## Supplementary figure S5

## Scheme 1


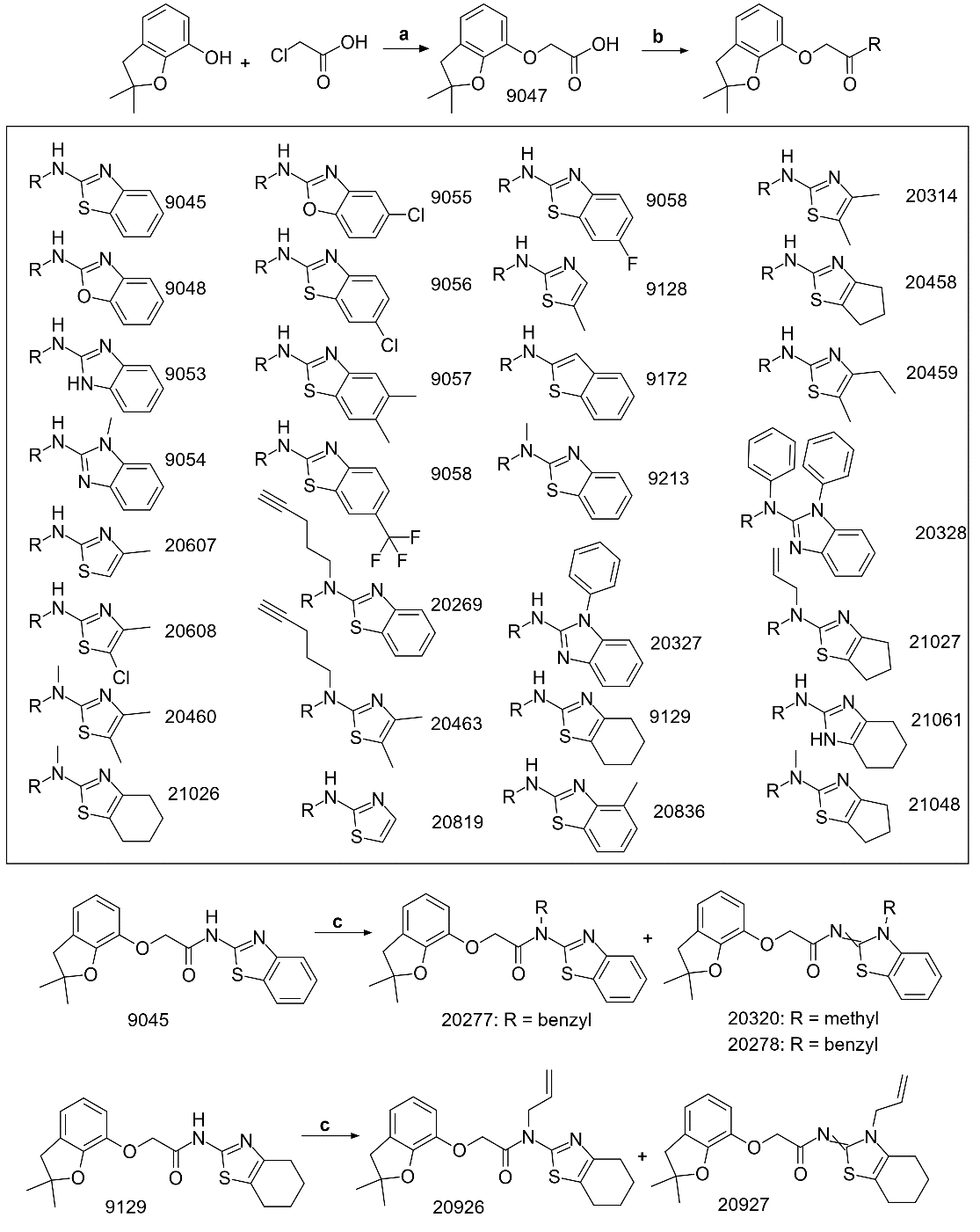


## Scheme 2


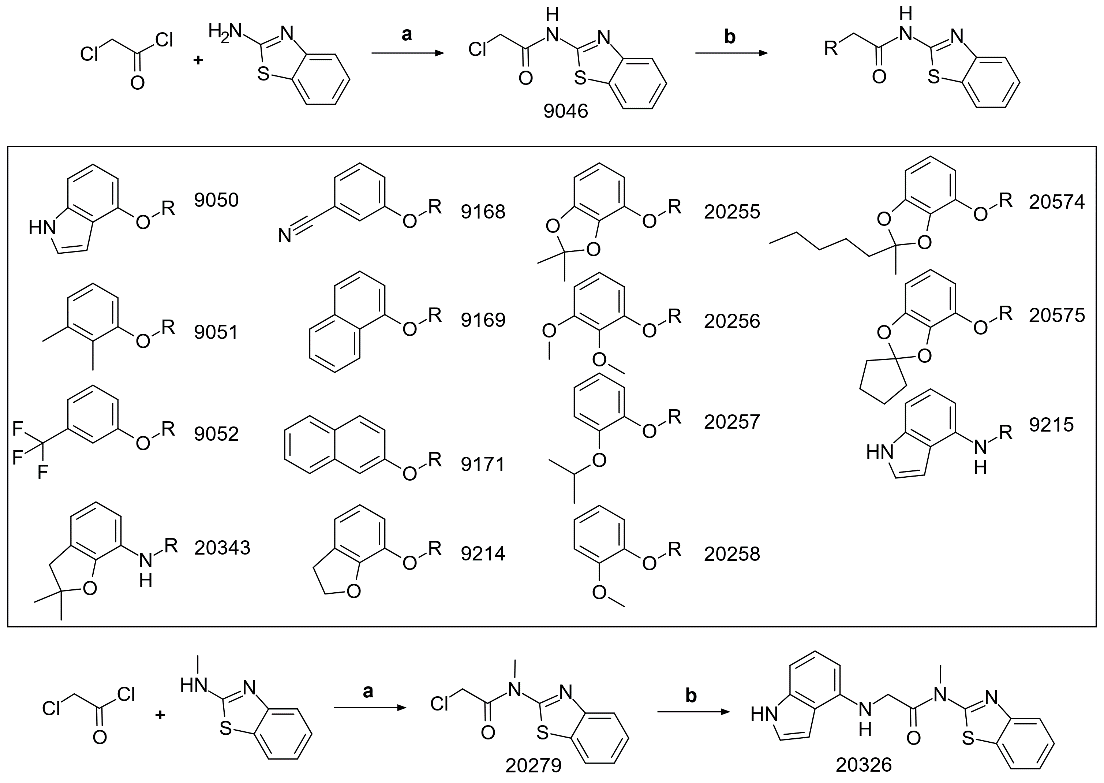


**Scheme 3**


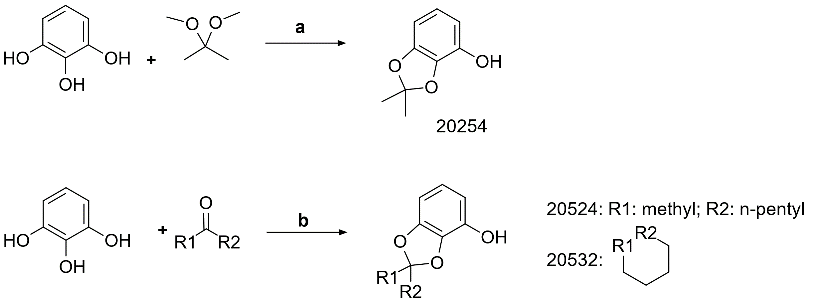


**Scheme 4**


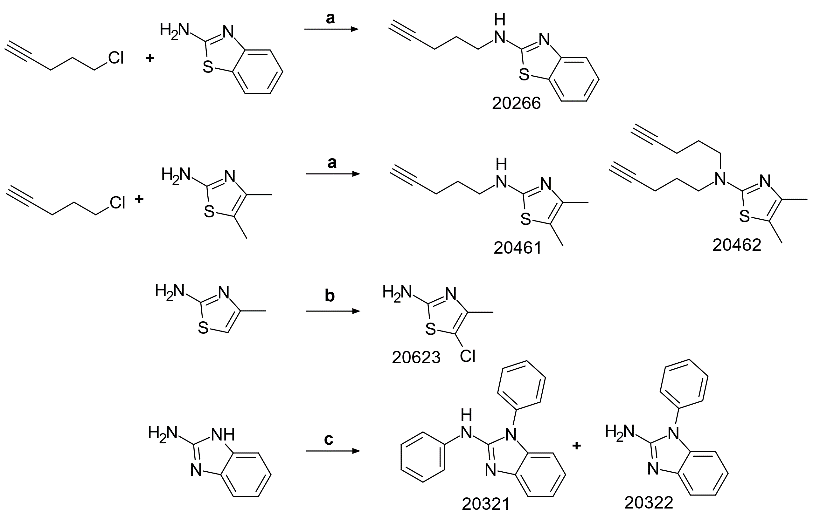


**Scheme 5**


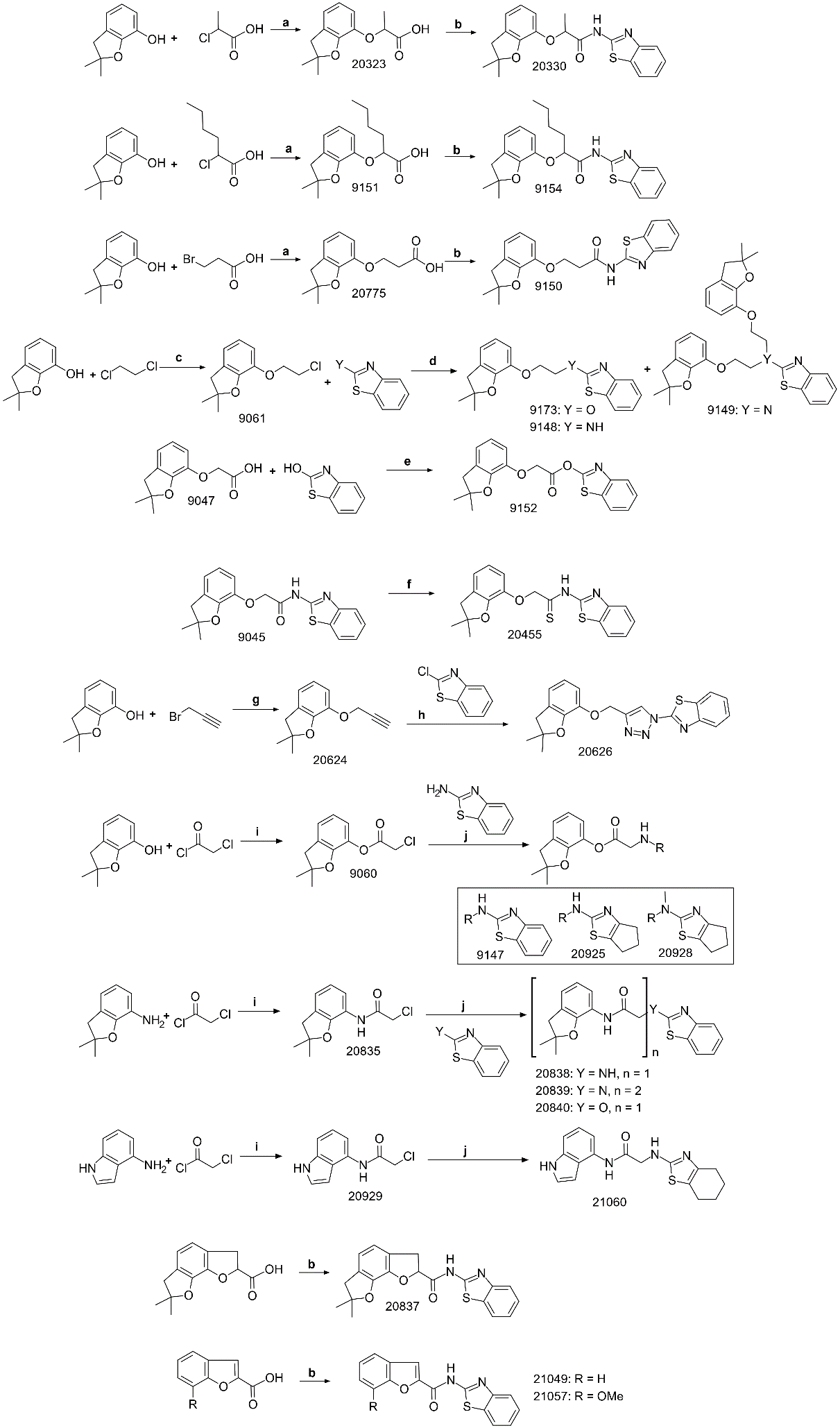


**Scheme 6**


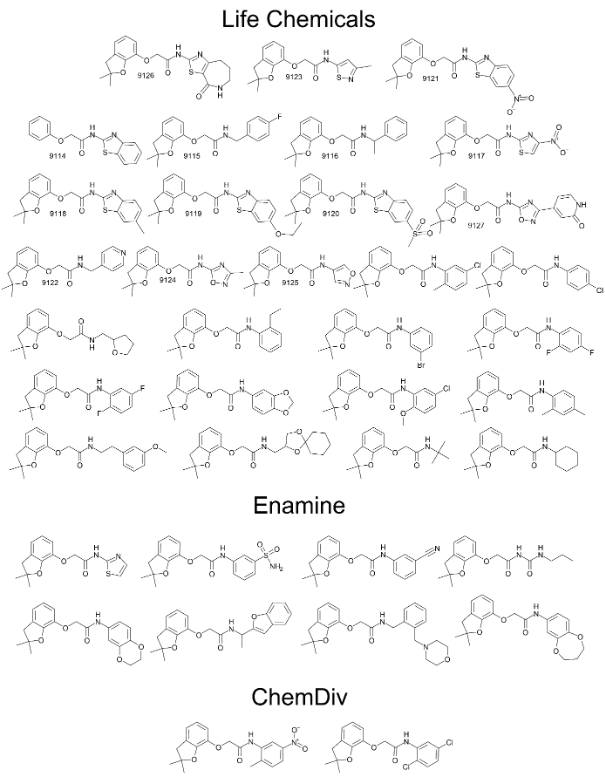

Supplement: FIG S5 [file mbio.02621-21-sf005.docx]

| **Residue** | **ID** | | **IC50** | **Residue** | **ID** | **IC50** |
| --- | --- | --- | --- | --- | --- | --- |
| 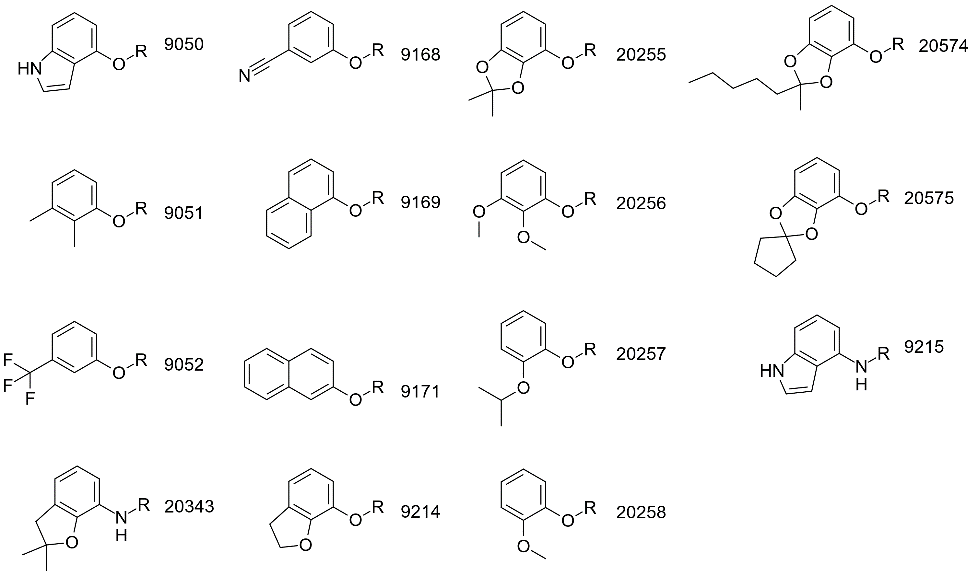 | 20574 | | 0.11 [0.072-0.17] µM | 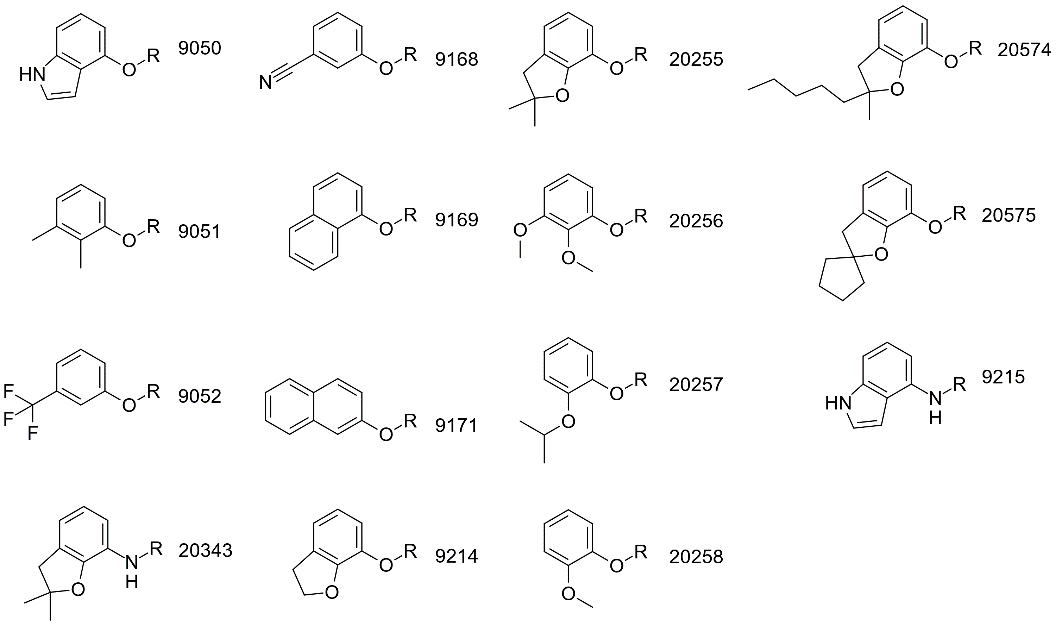 | 9169 | >100 µM |
| 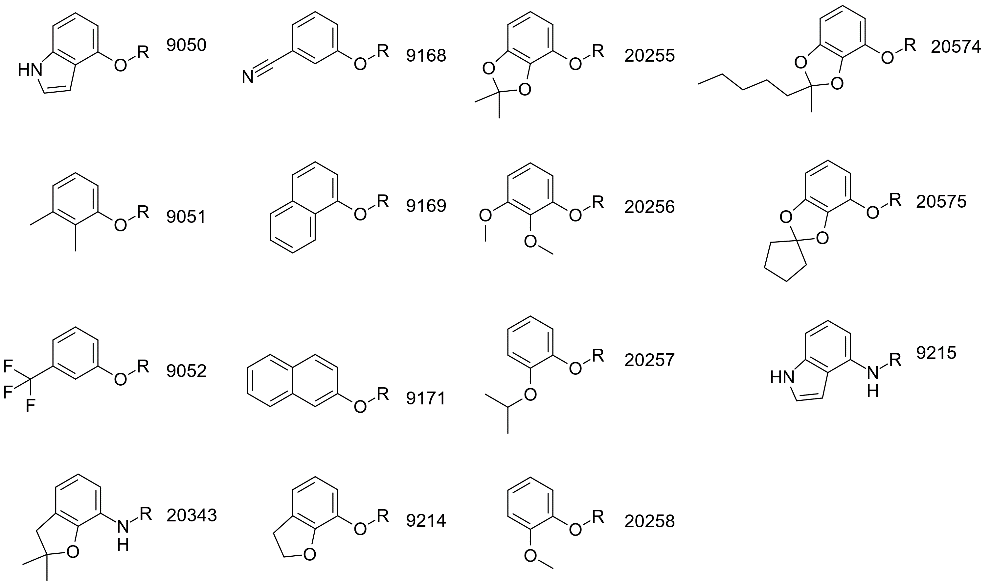 | 20575 | | 0.35 [0.25-0.49] µM | 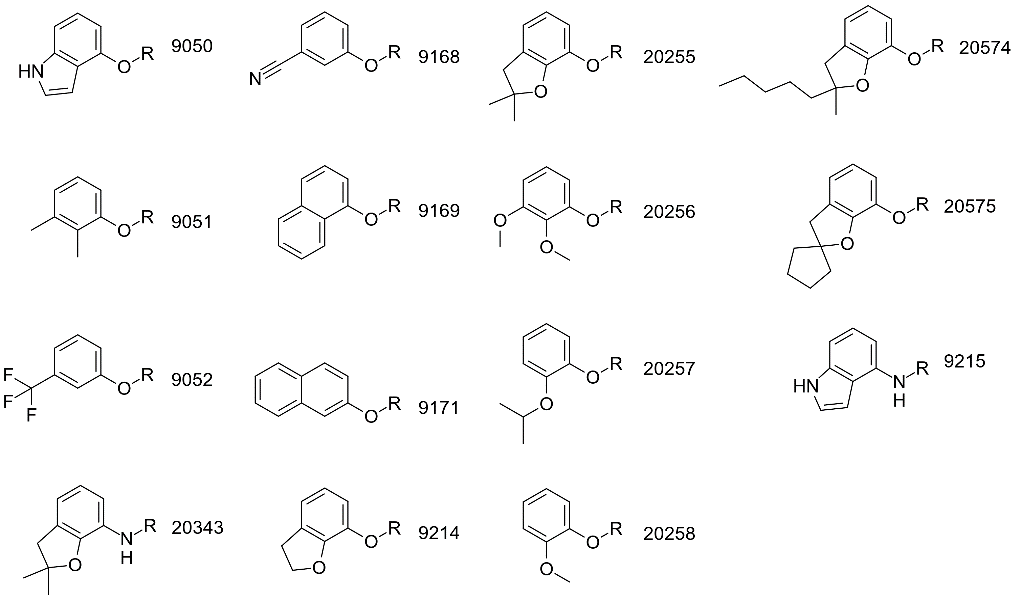 | 9171 | >100 µM |
| 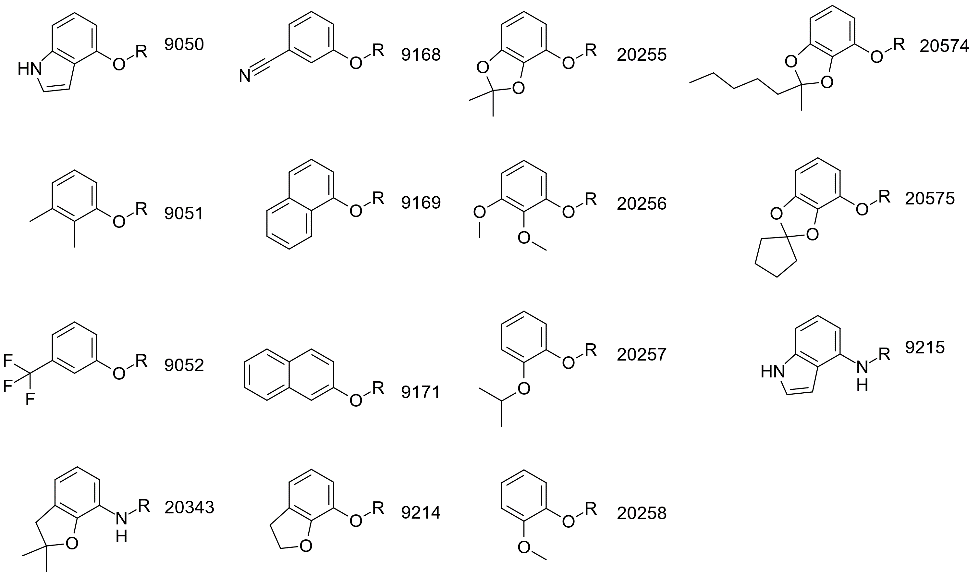 | 20255 | | 0.61 [0.48-0-79] µM  0.40 [0.32-0.50] µM | 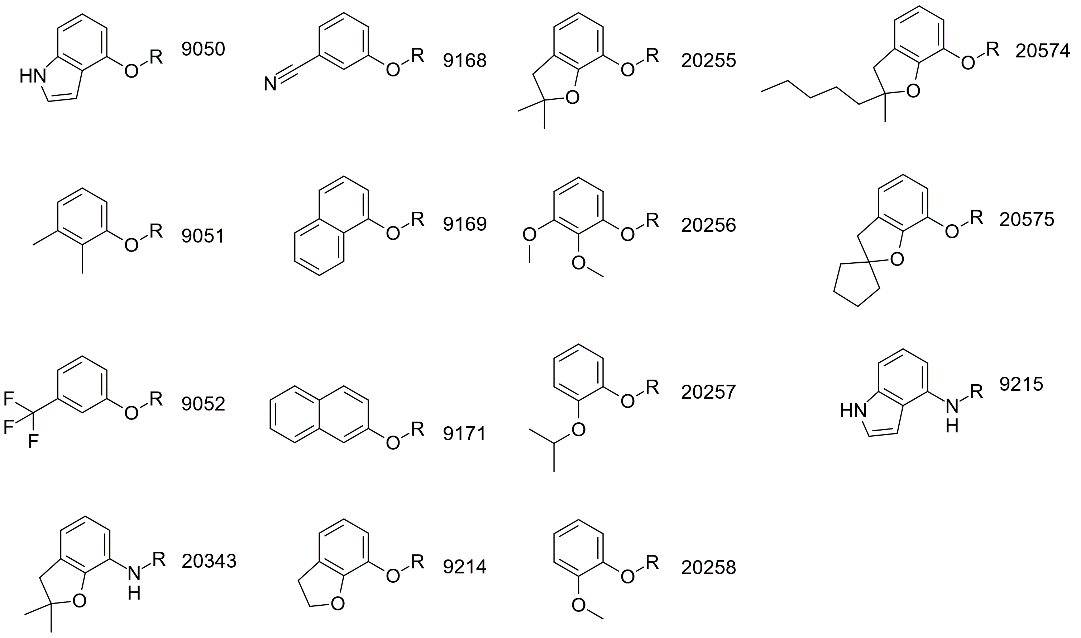 | 9114 | >100 µM |
| 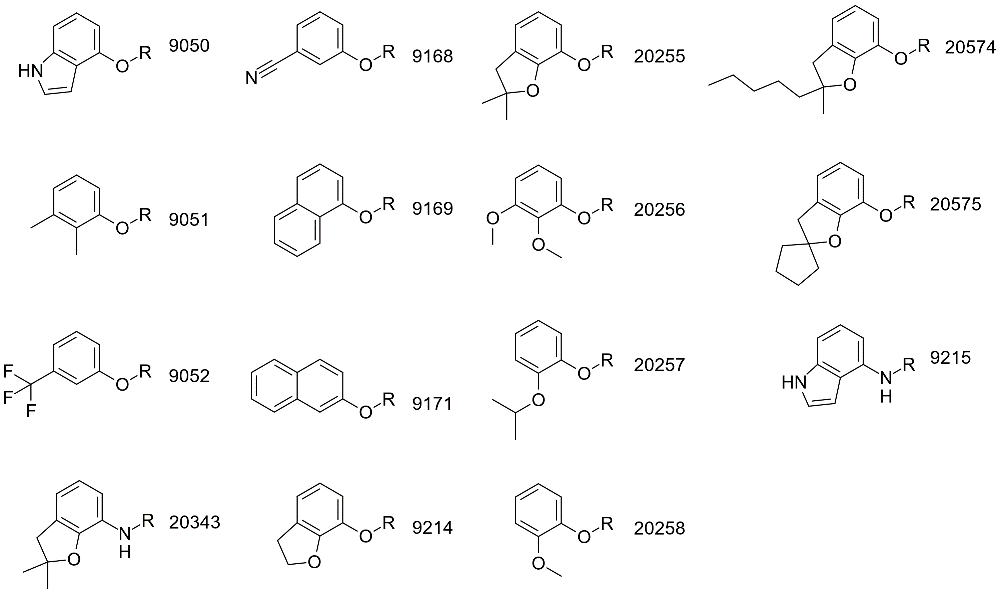 | 9215 | | 0.73 [0.41-1.2] µM | 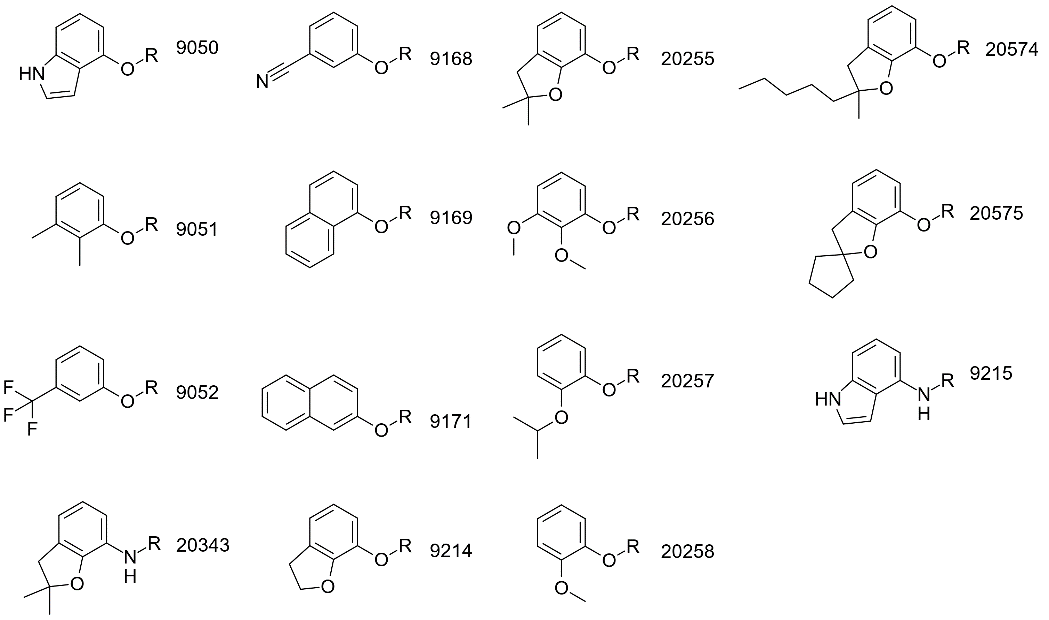 | 20256 | >100 µM |
| 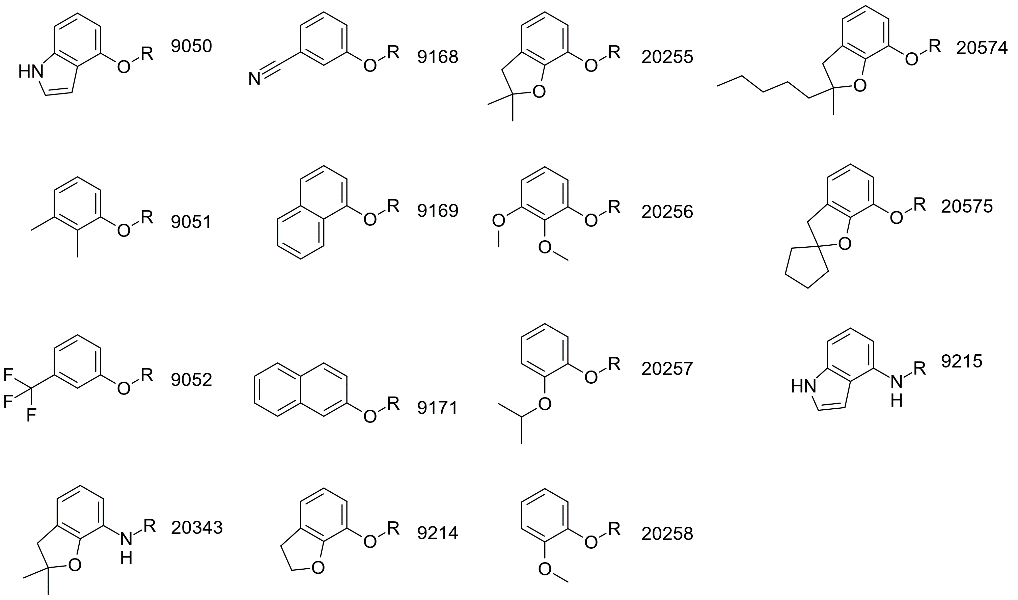 | 20343 | | 1.7 [1.1-2.5] µM | 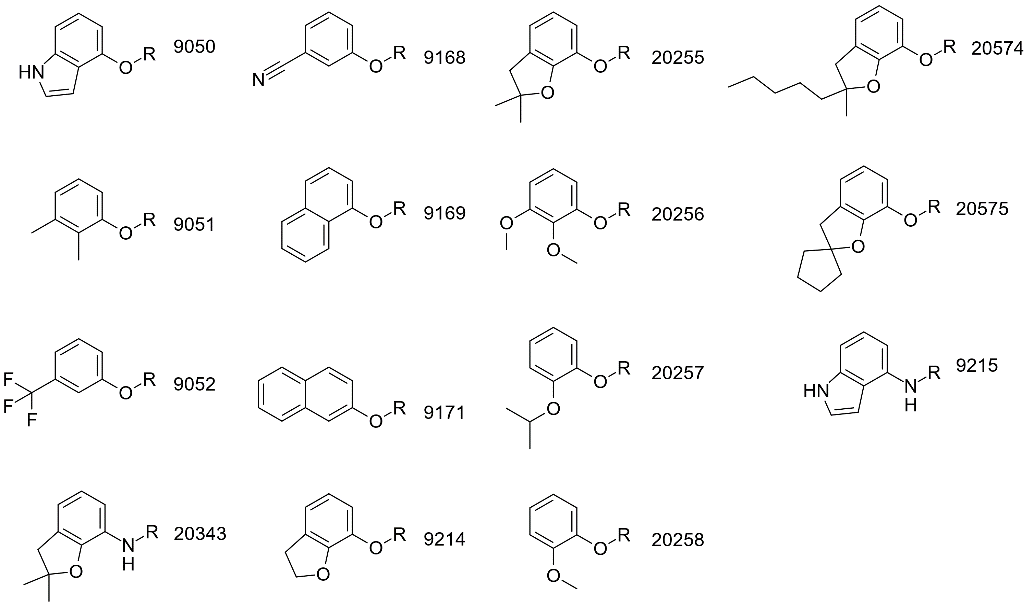 | 20258 | >100 µM |
| 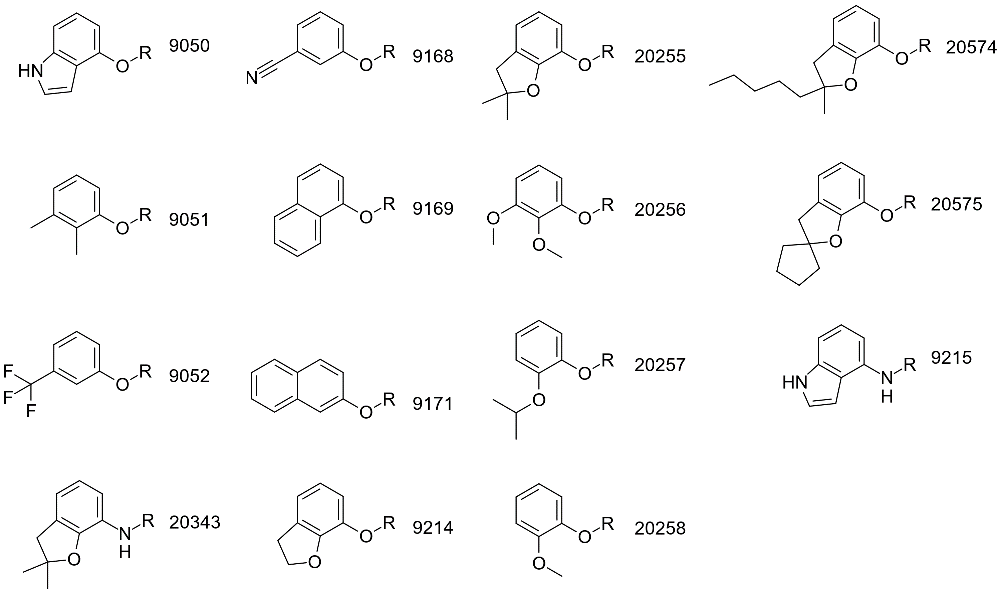 | 9050 | | 8.3 [6.3-11] µM | 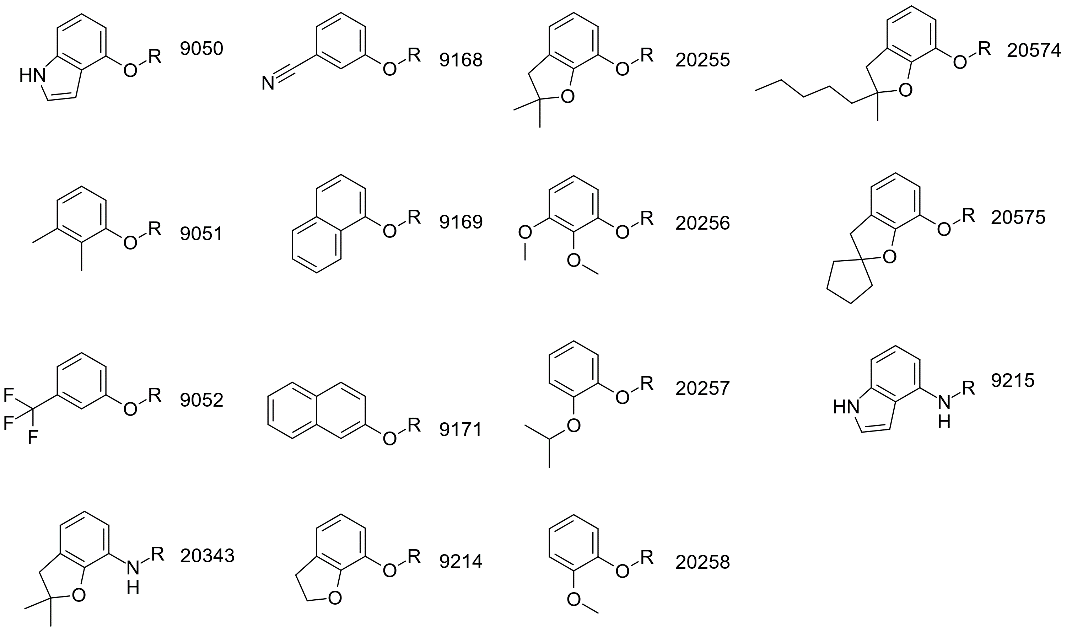 | 9051 | >100 µM |
| 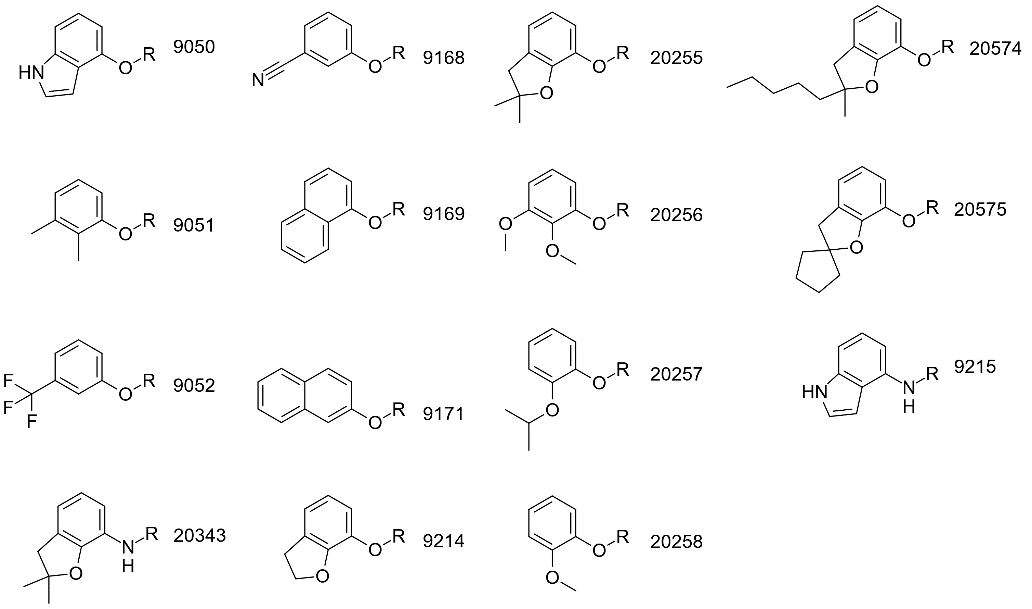 | 20257 | | 48 [31-77] µM | 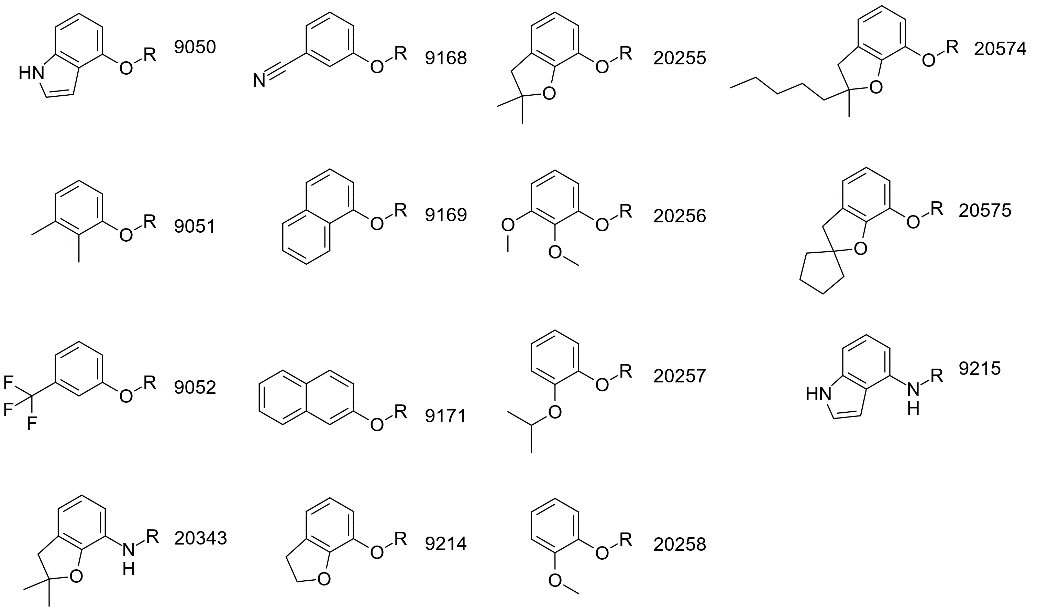 | 9052 | >100 µMs |
| 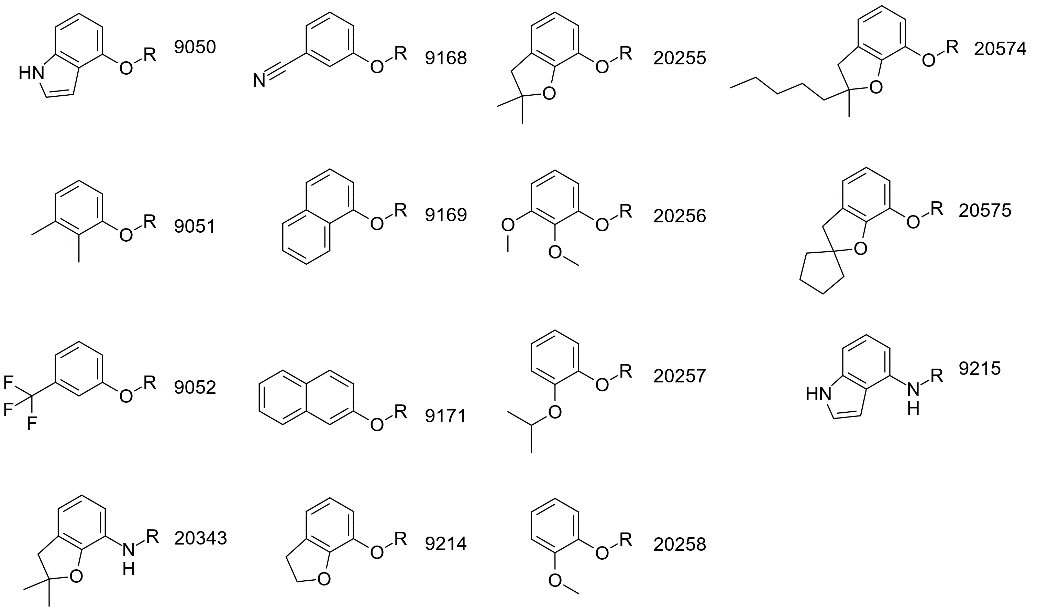 | 9214 | | 63 [45-93] µM | 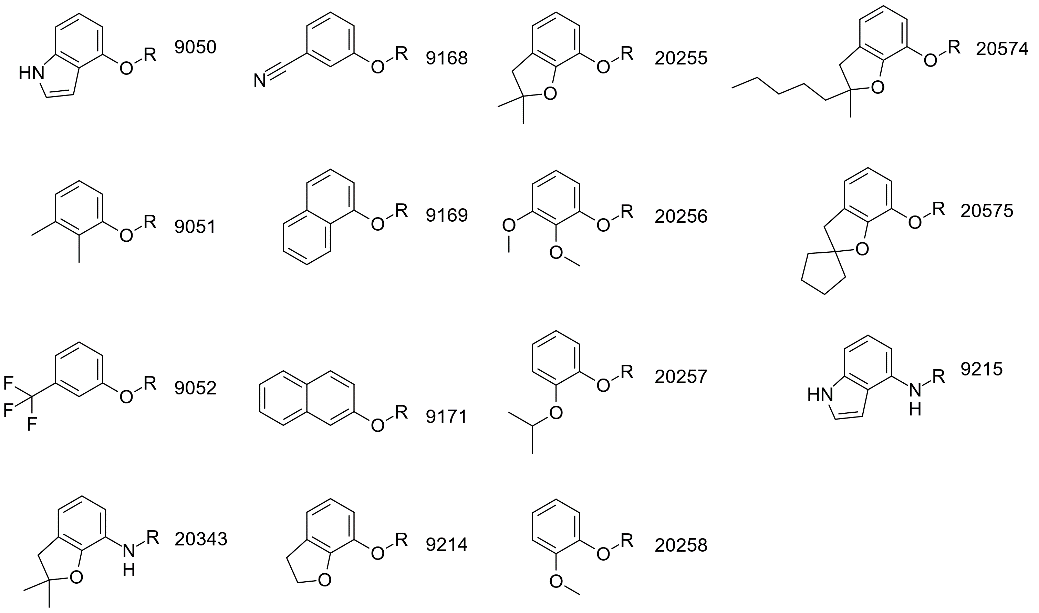 | 9168 | >100 µM |
|  |  | |  |  |  |  |
| **R =** | | **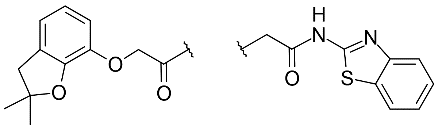** | | | | |

Supplement: TABLE S1 [file mbio.02621-21-st001.docx]

| **Residue** | **ID** | **IC50** | **Residue** | **ID** | **IC50** |
| --- | --- | --- | --- | --- | --- |
| 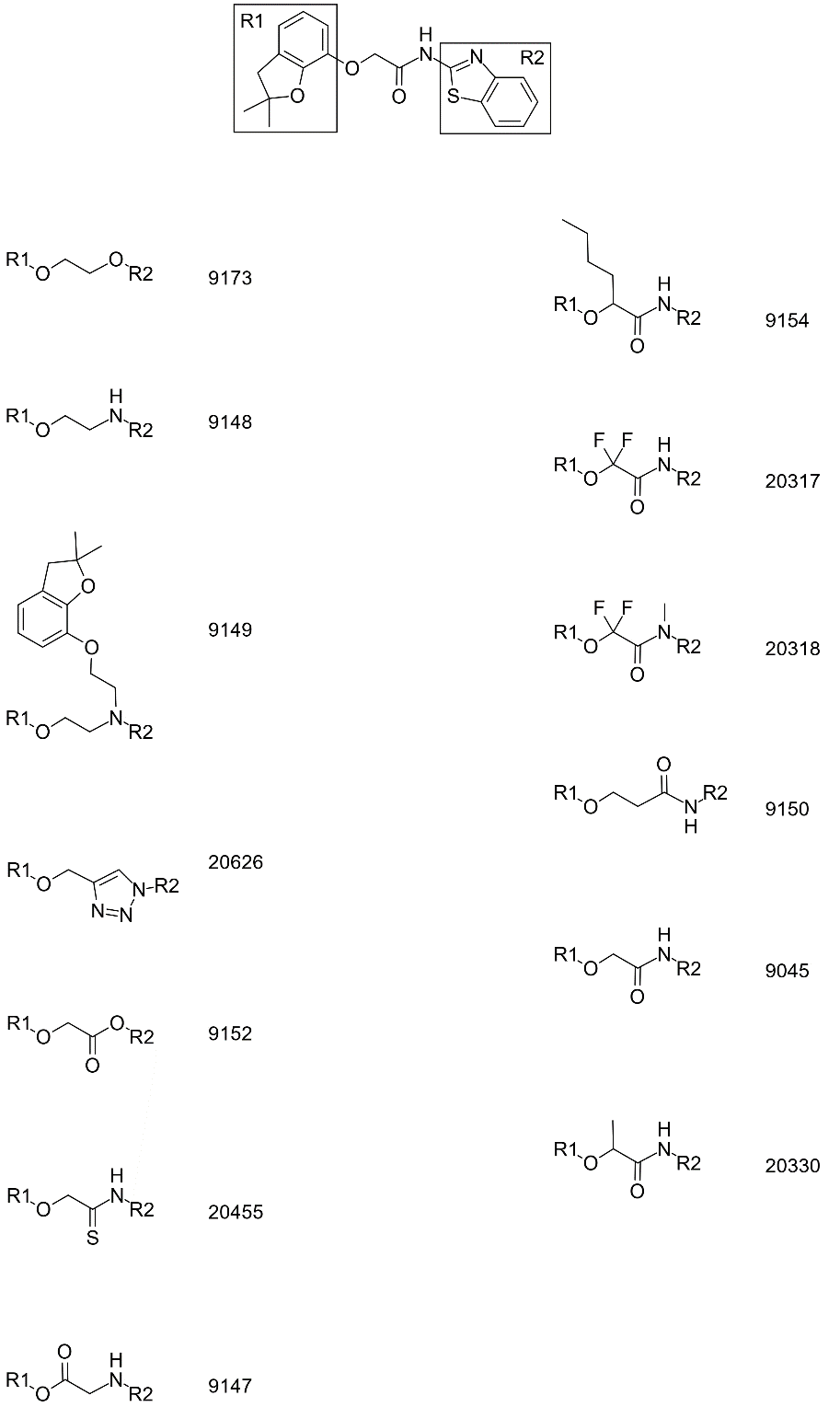 | 9045 | 0.52 [0.42-0.64] µM | 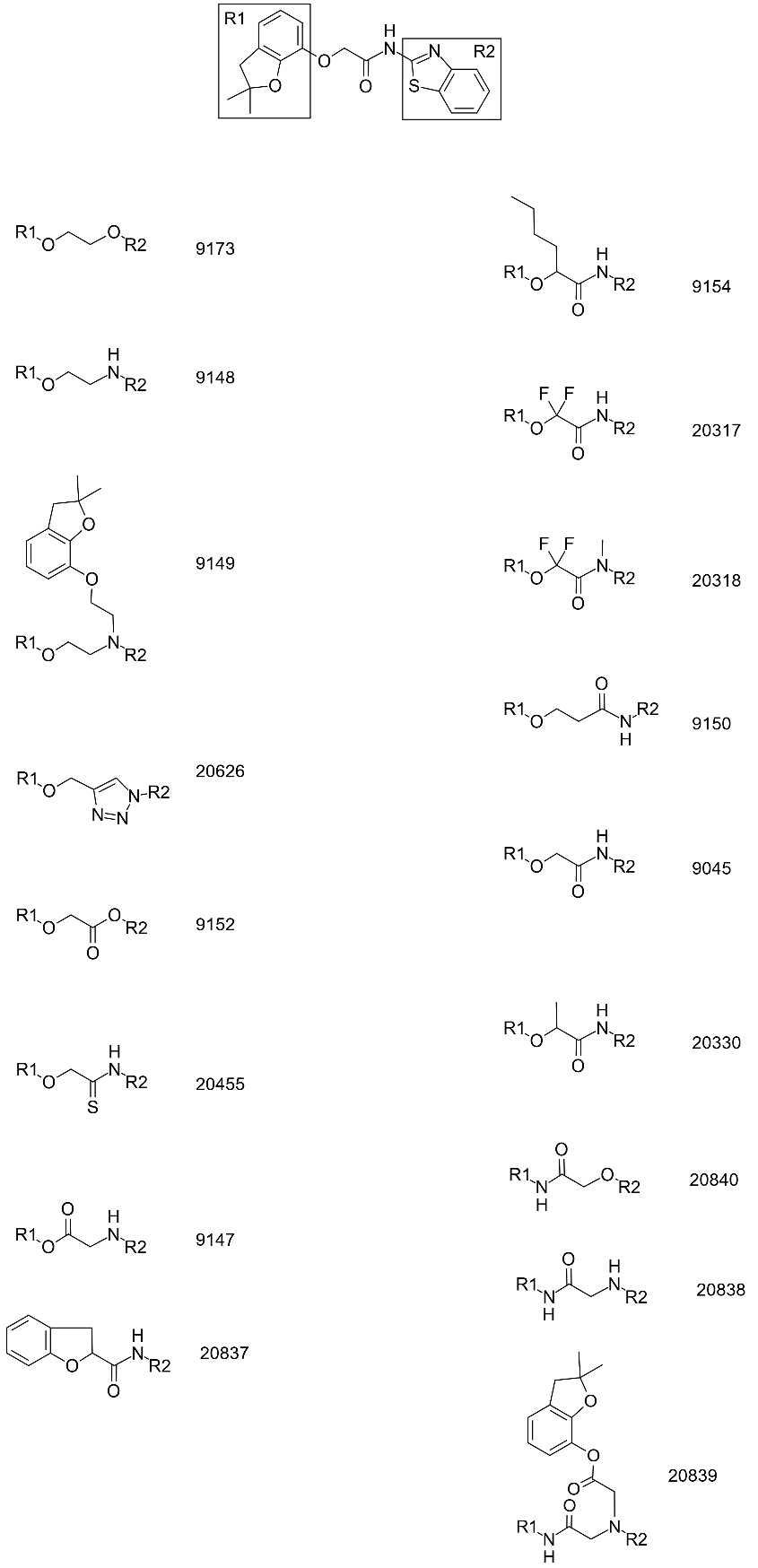 | 20838 | >100 µM |
| 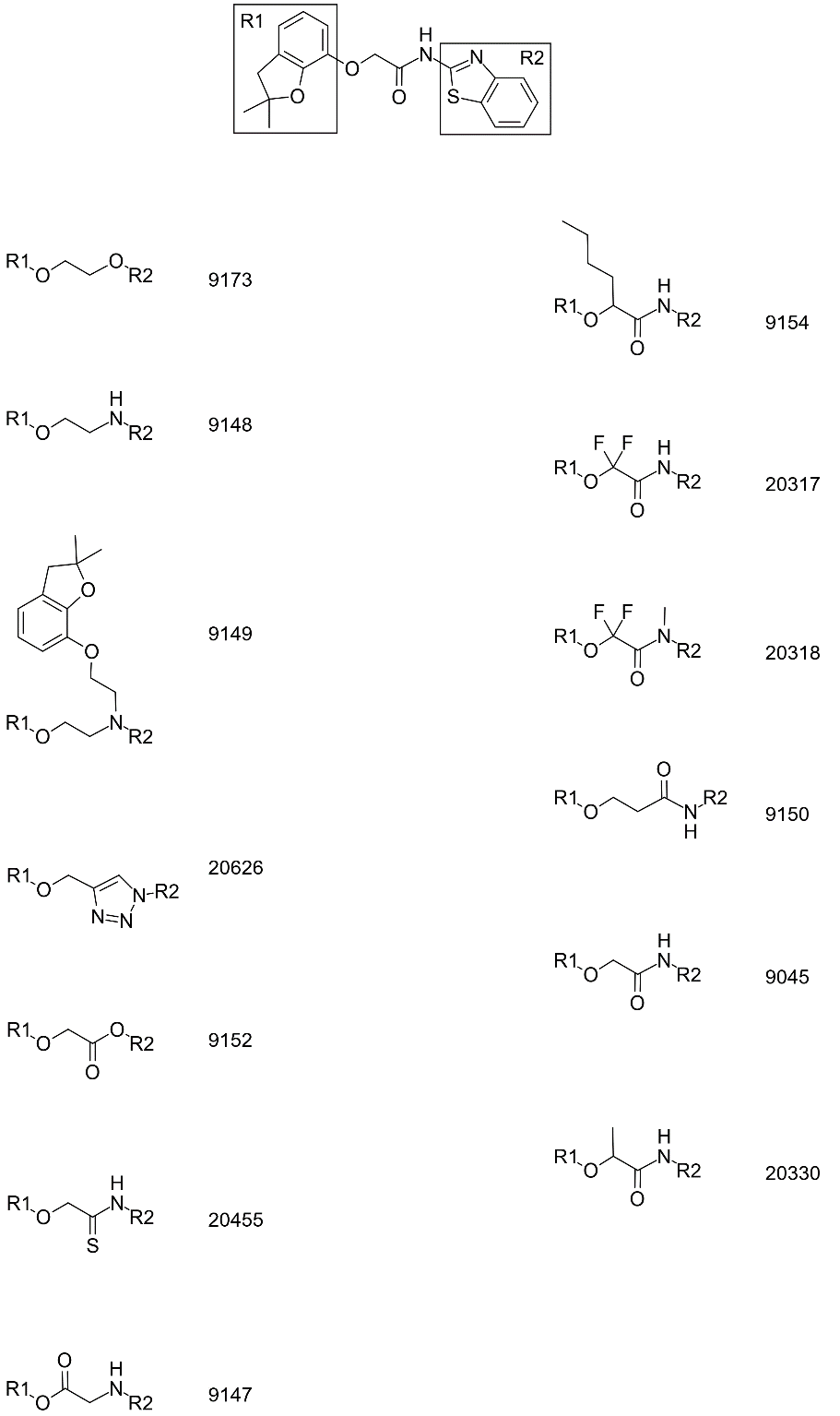 | 9147 | 0.48 [0.37-0.63] µM | 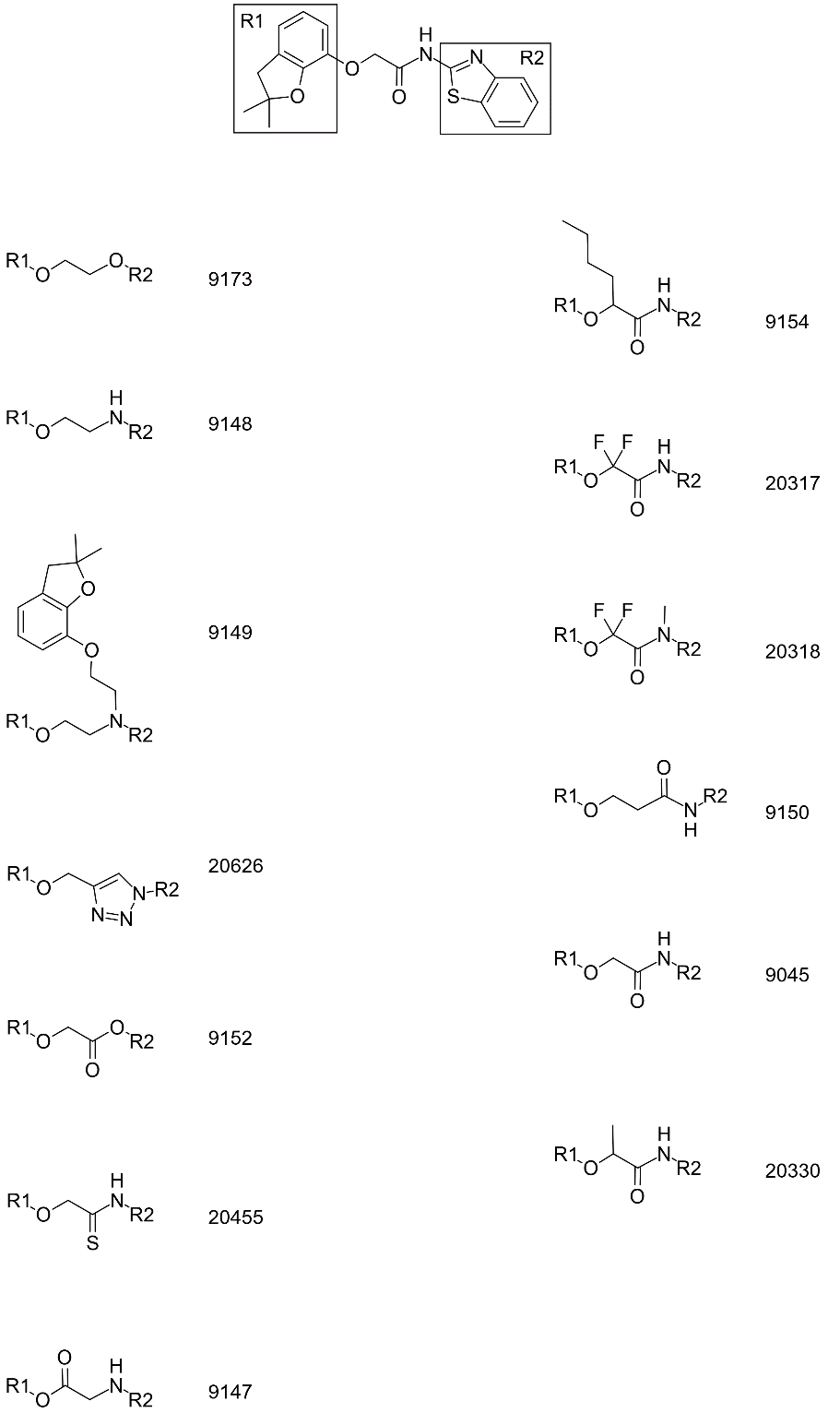 | 9154 | >100 µM |
| 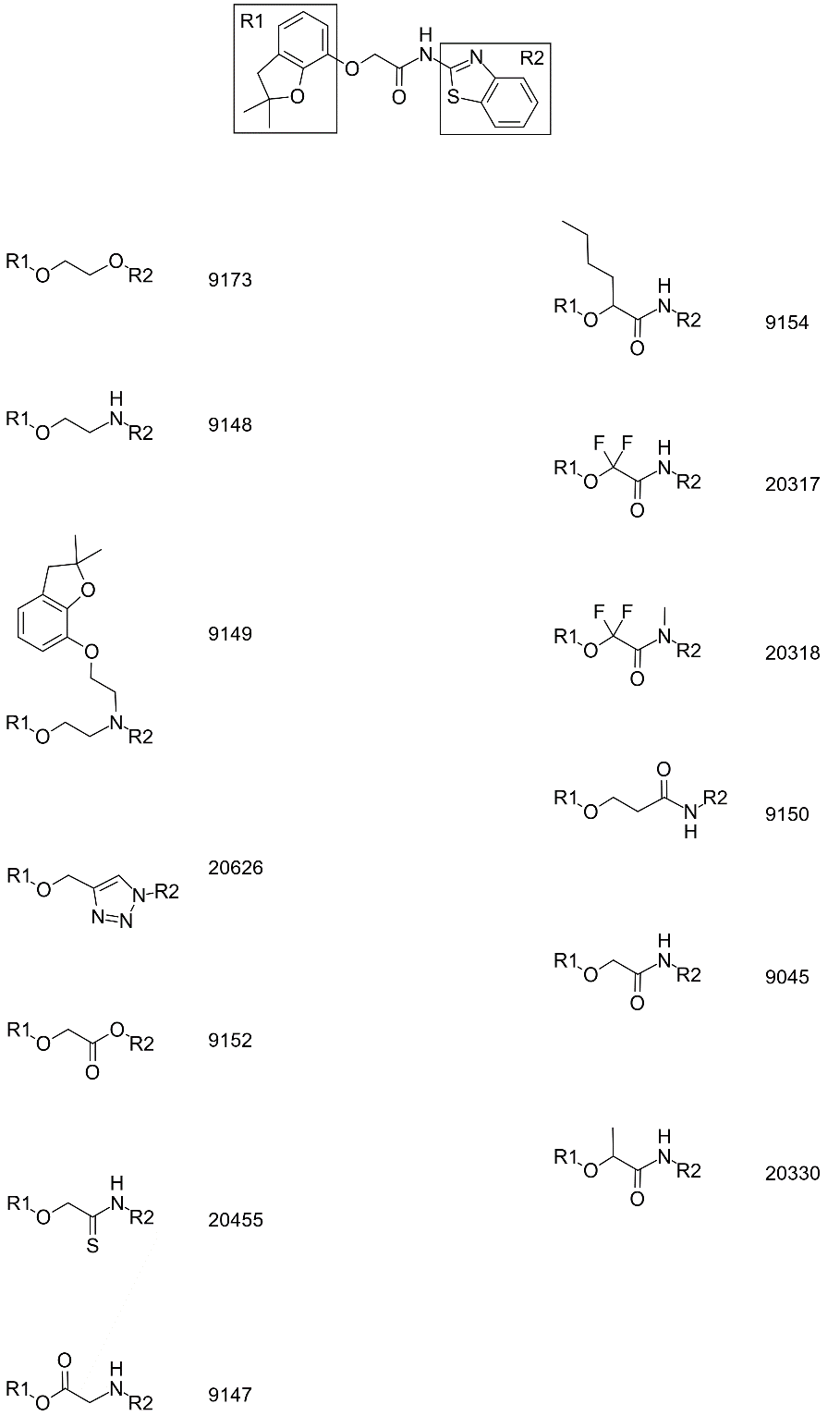 | 9149 | 26 [10-67] µM | 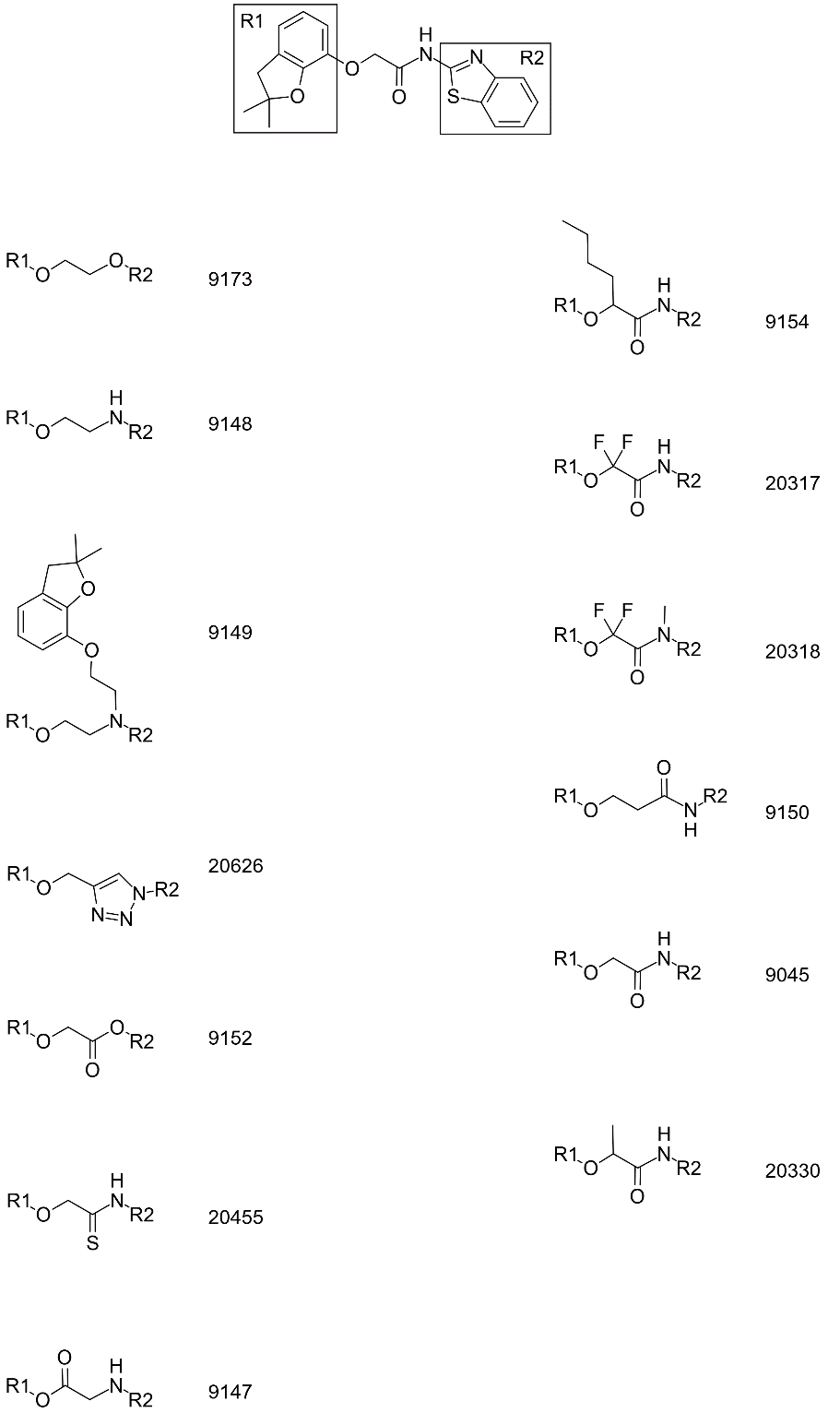 | 9152 | >100 µM |
| 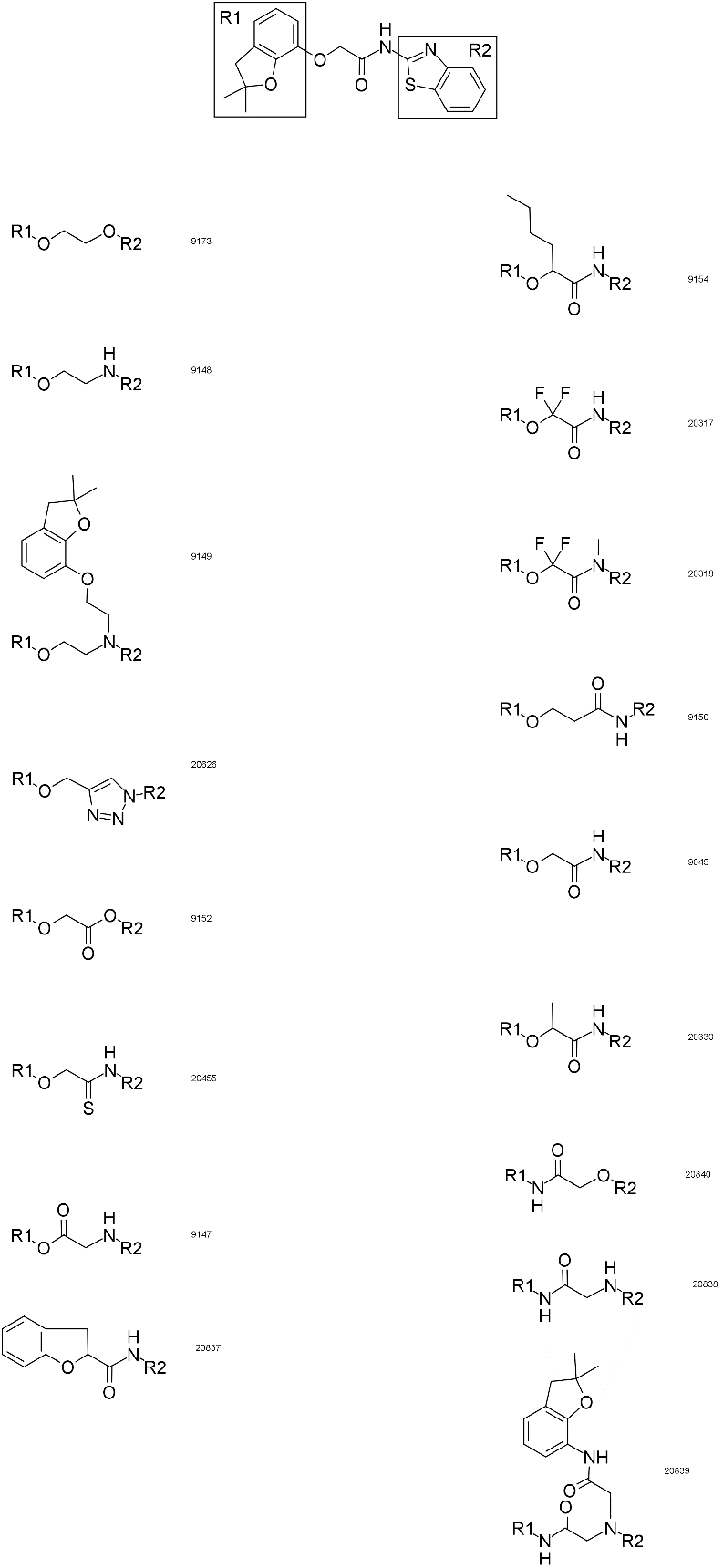 | 20839 | 44 [12 - 89] µM | 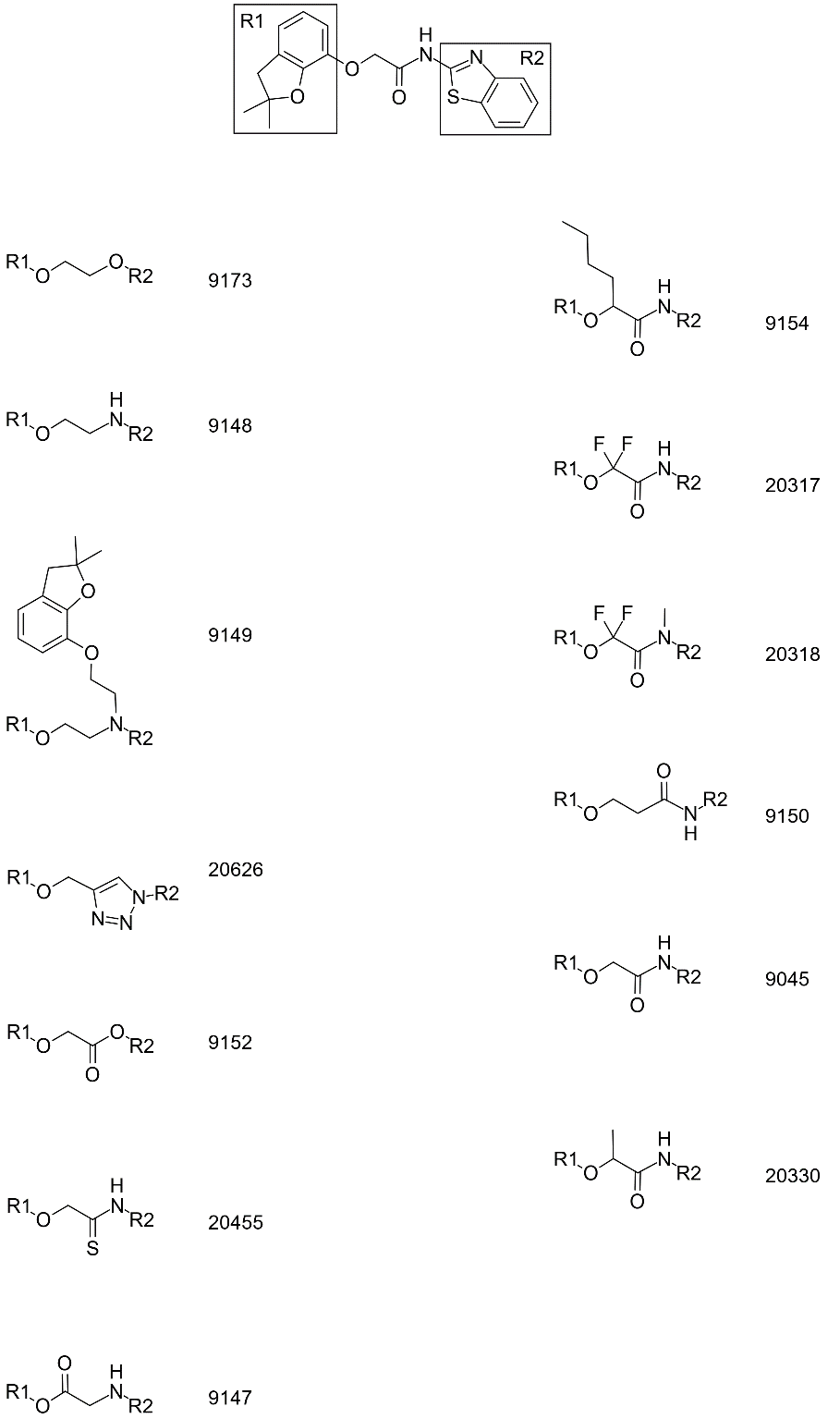 | 9173 | >100 µM |
| 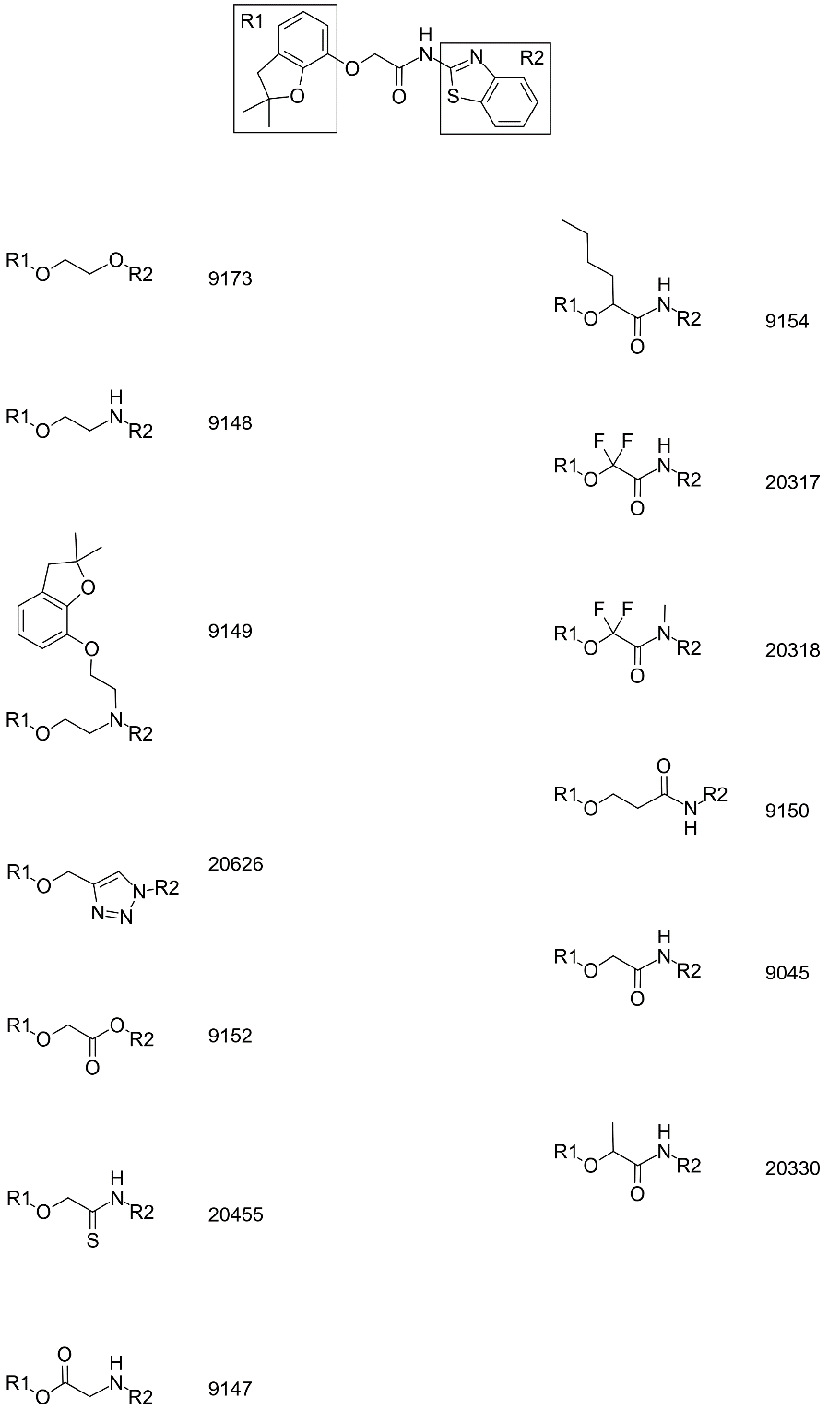 | 20330 | 53 [21-142] µM | 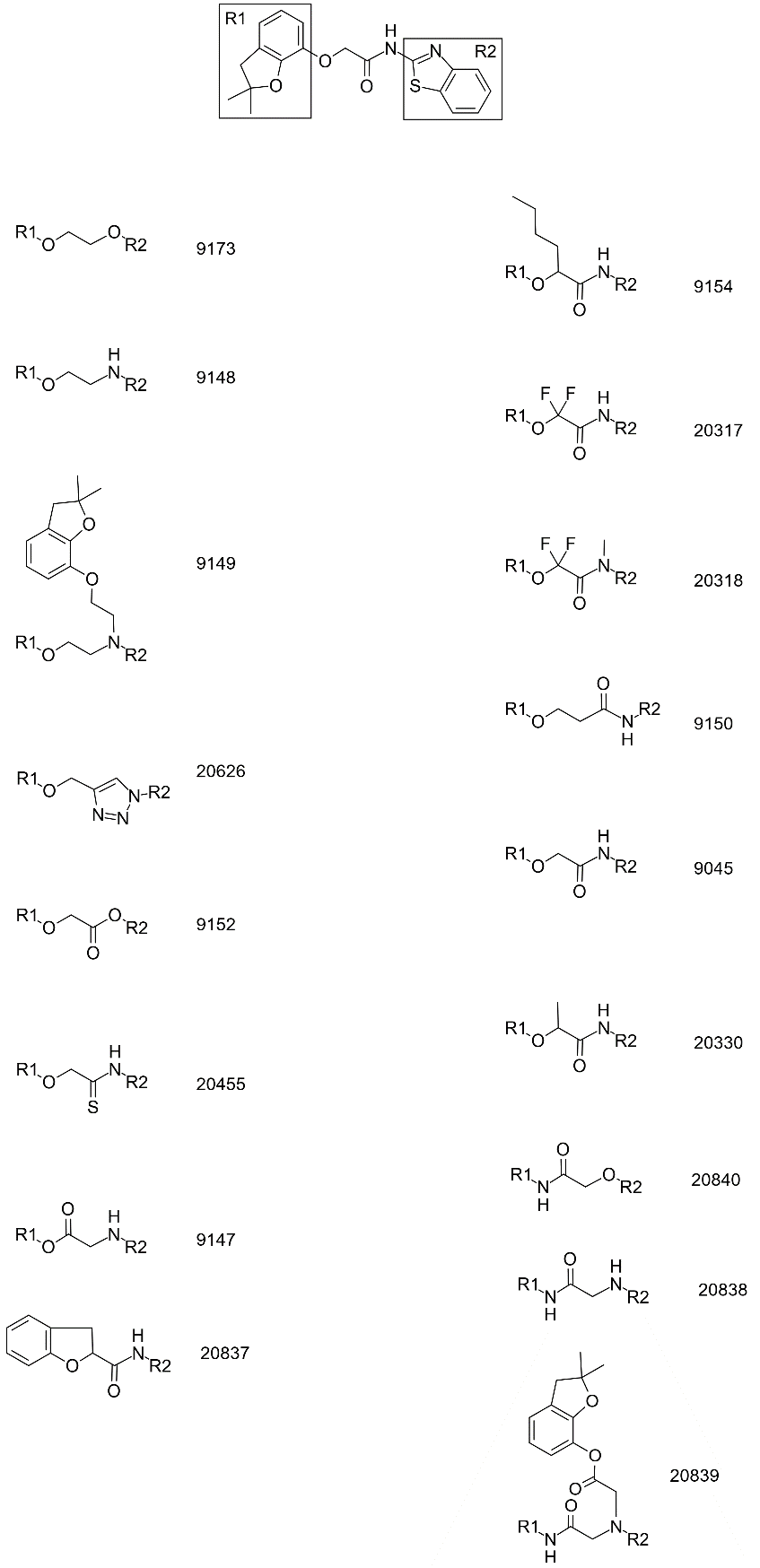 | 20840 | >100 µM |
| 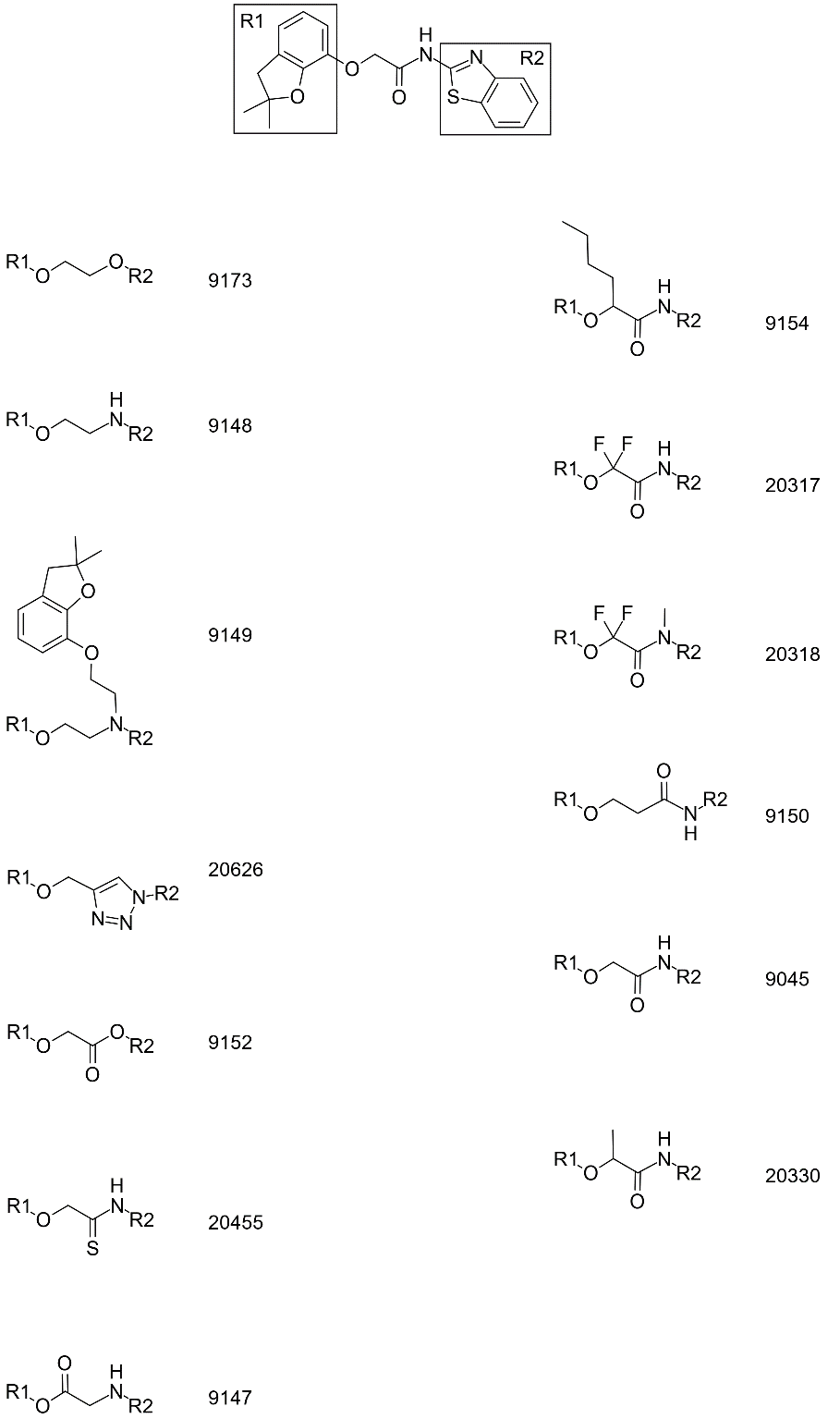 | 20455 | 59 [26-142] µM | 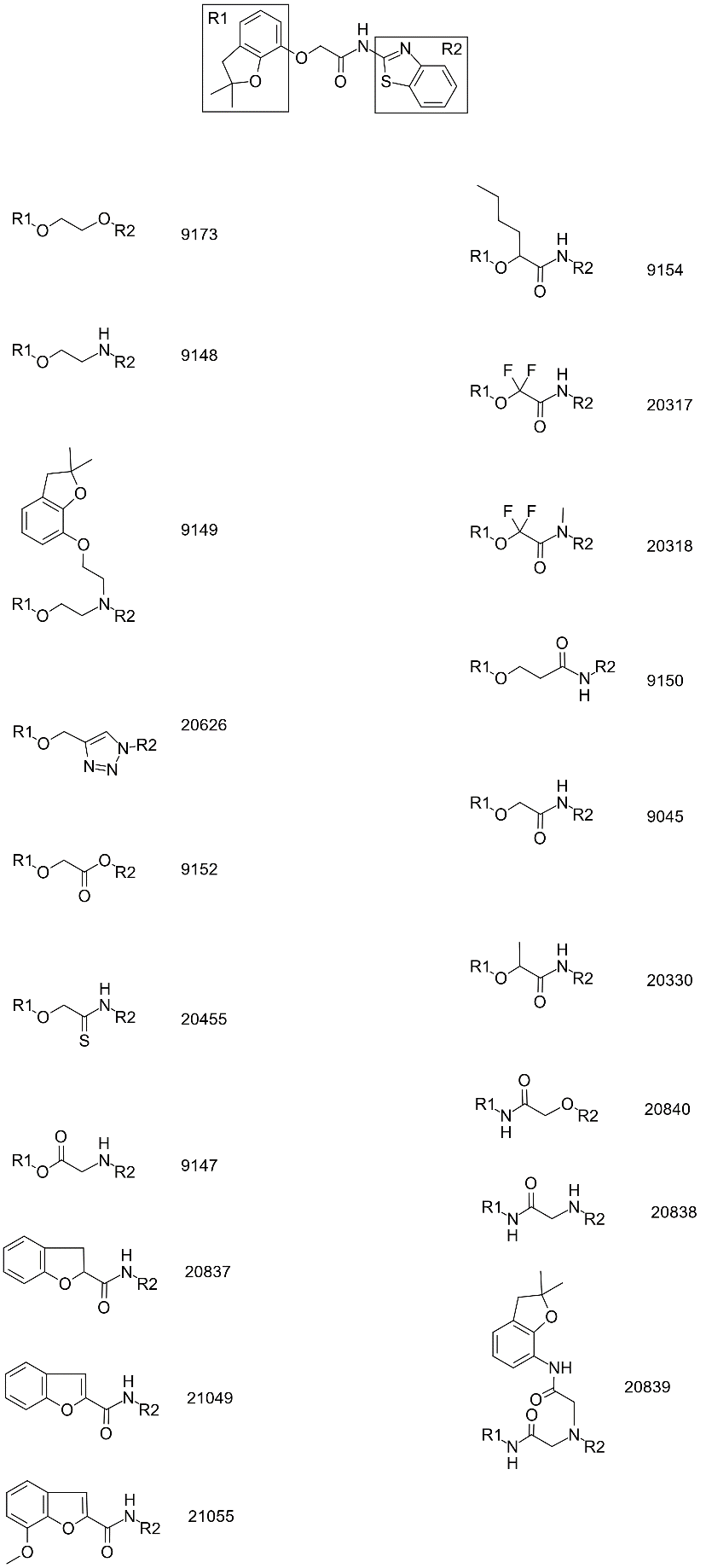 | 21057 | >100 µM |
| 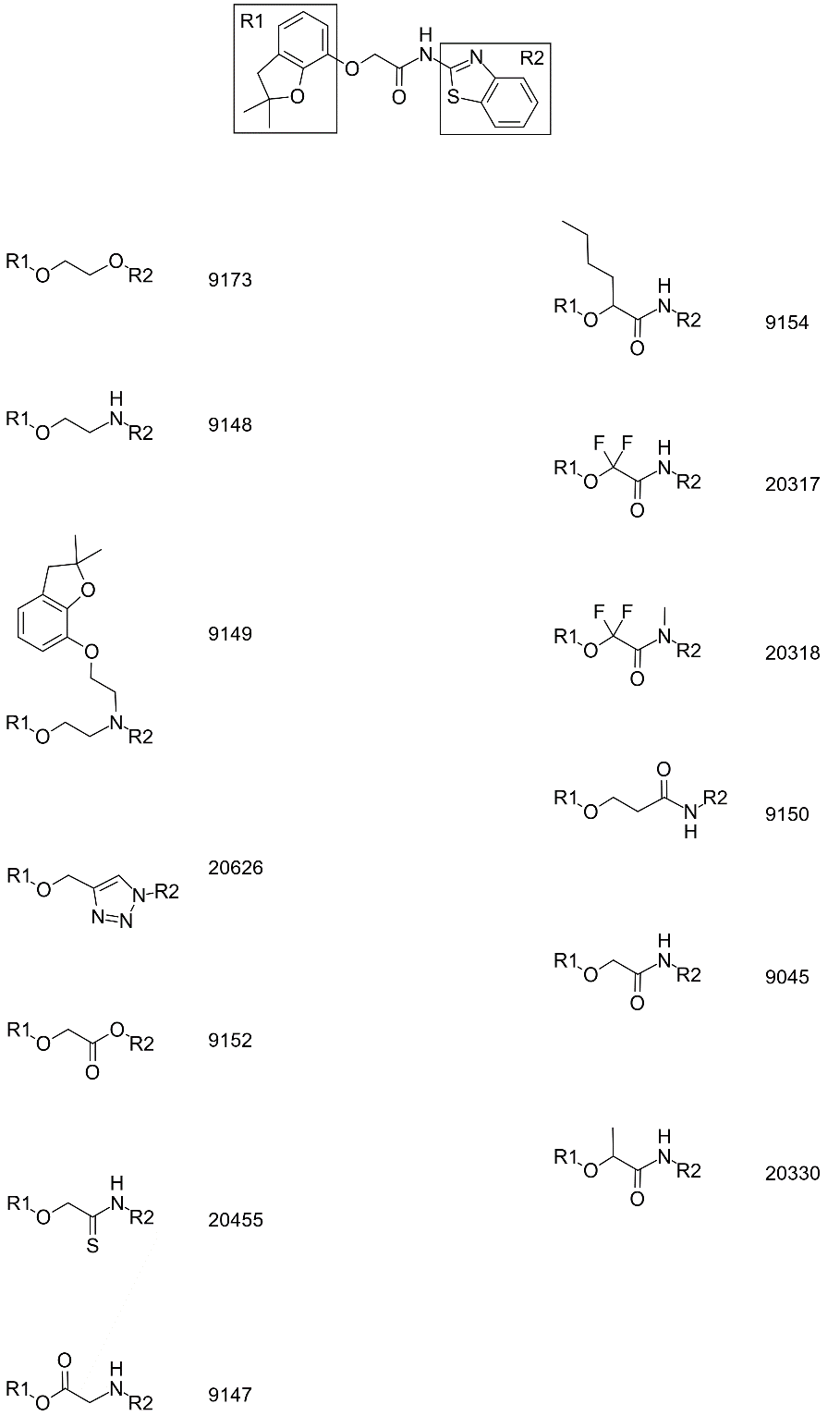 | 9148 | 85 [42-172] µM | 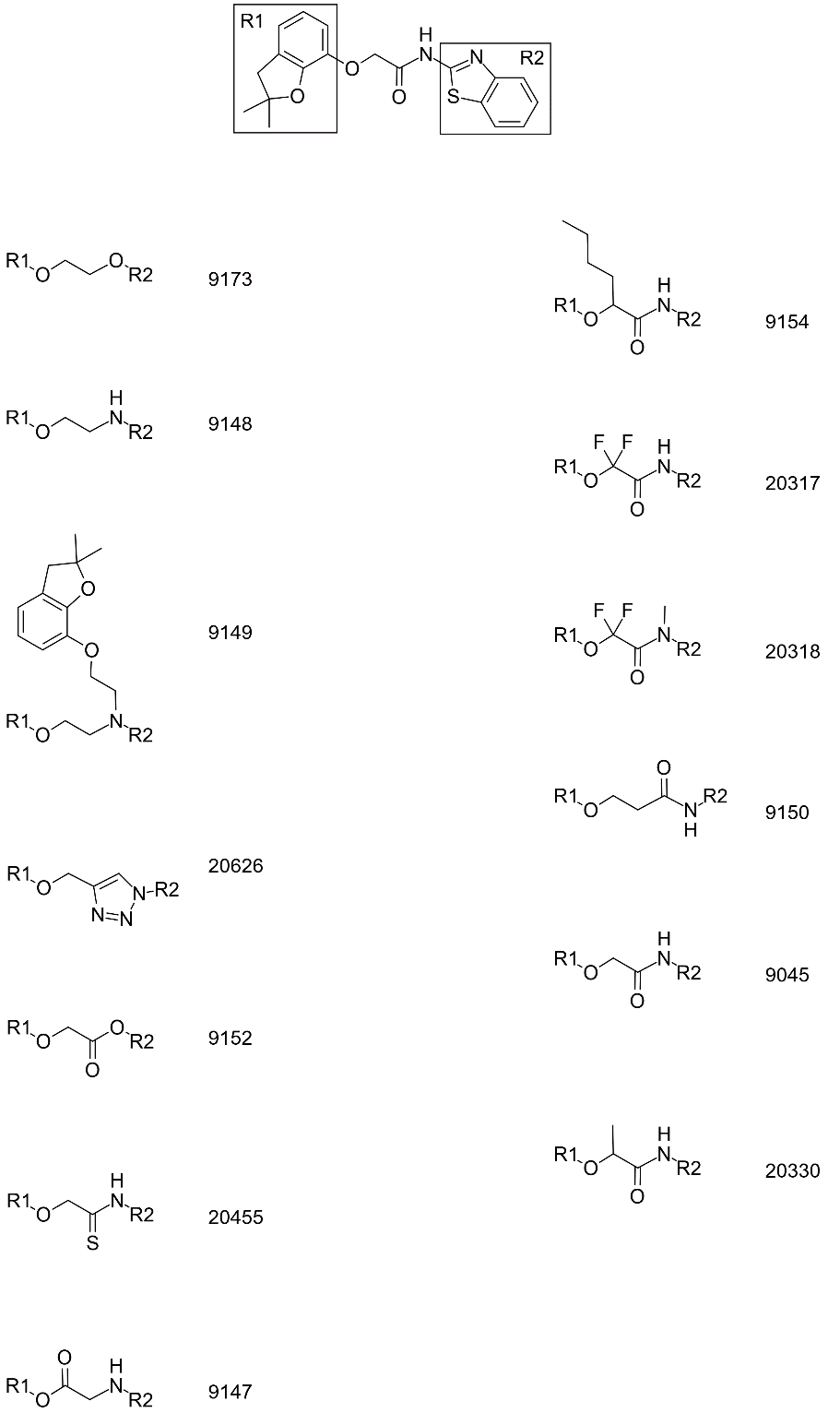 | 20626 | >100 µM |
| 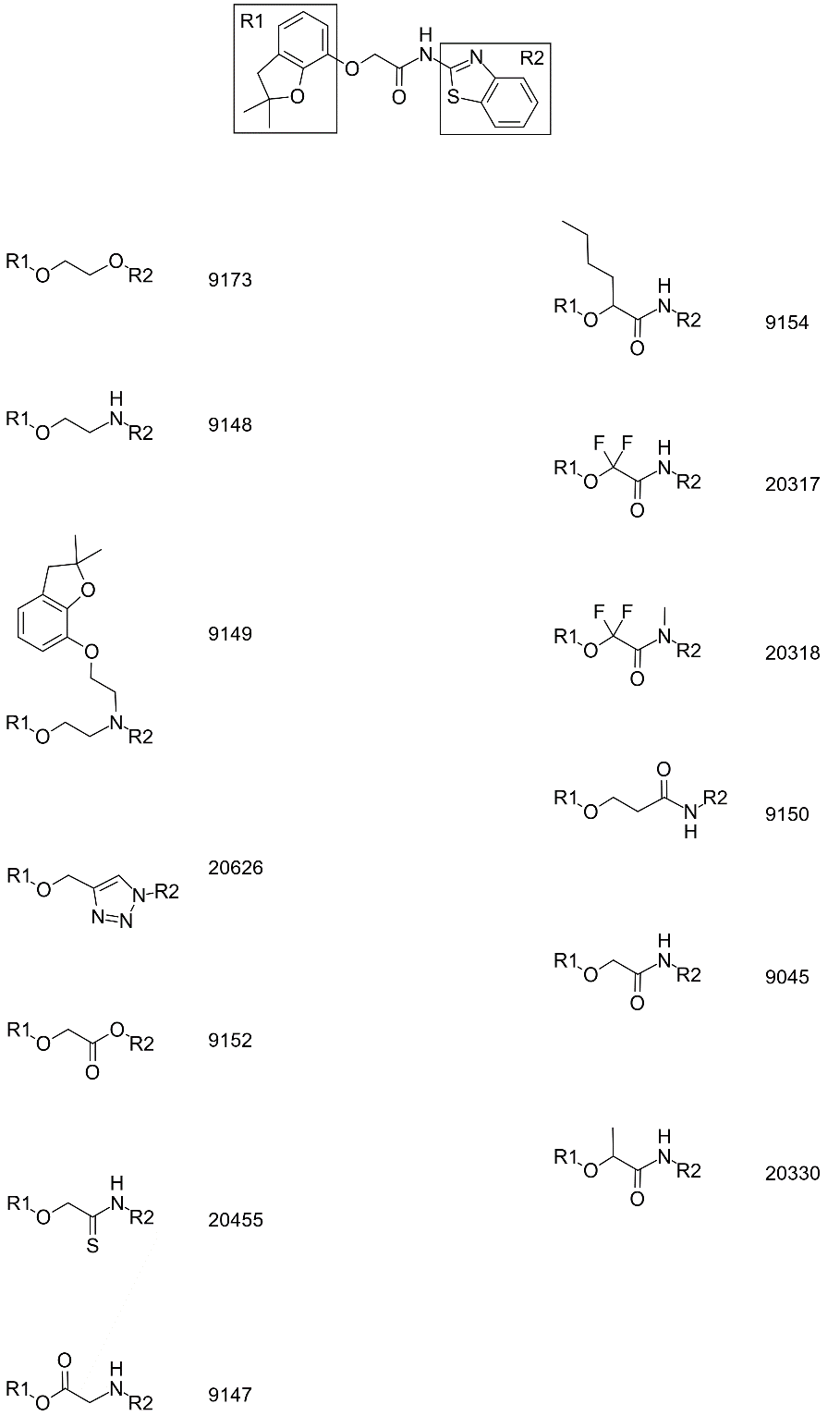 | 9150 | 91 [54-153] µM | 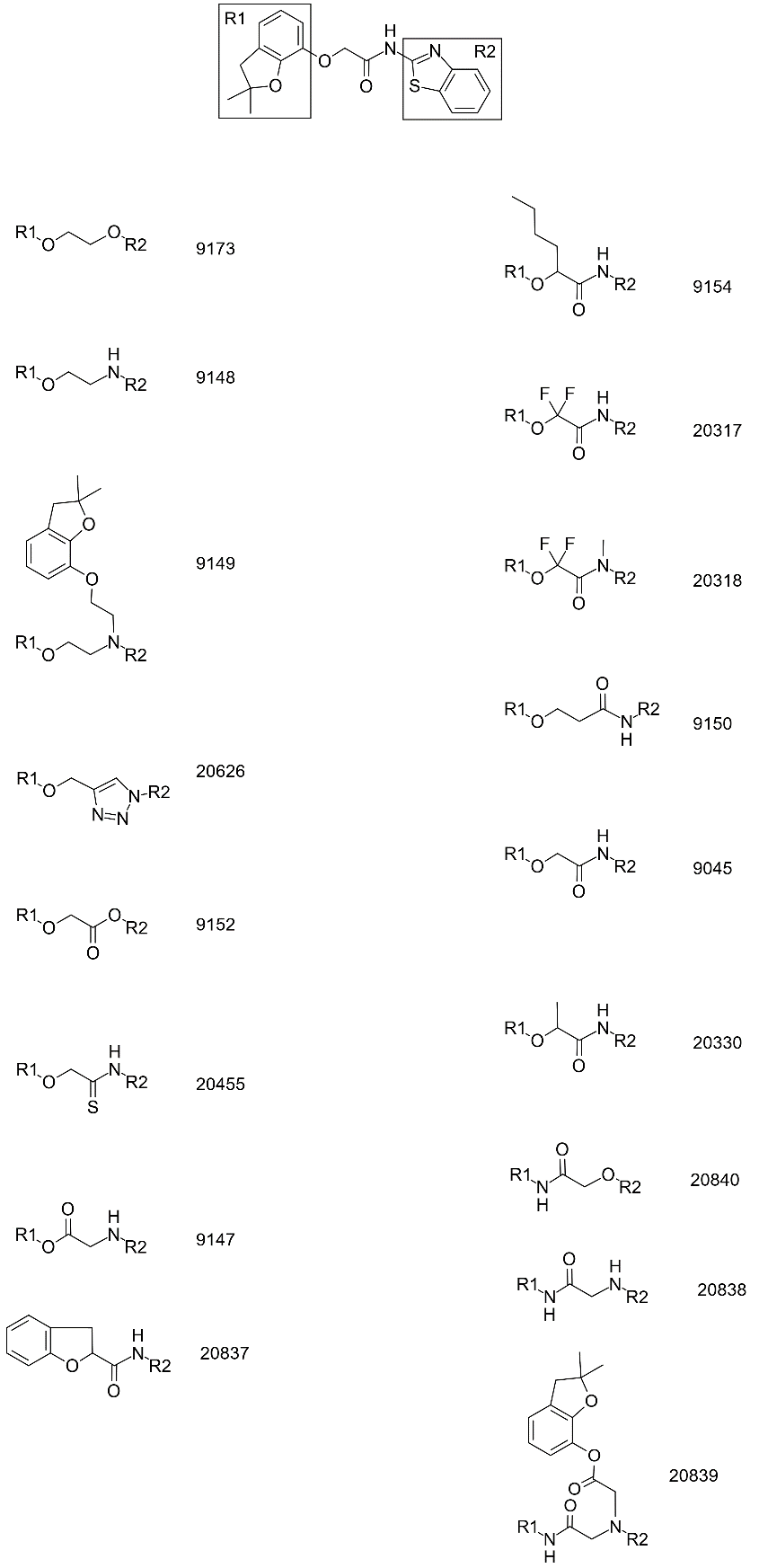 | 20837 | >100 µM |
| 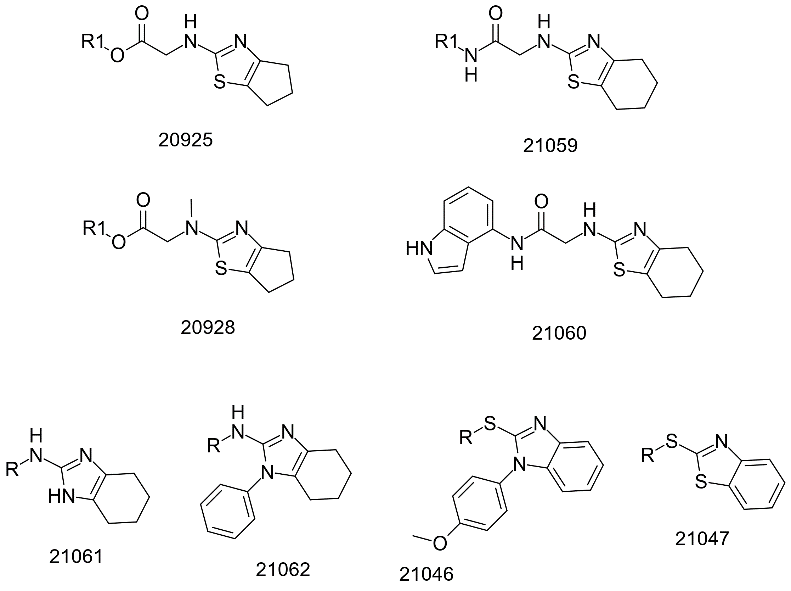 | 20928 | 0.076 [0.060-0.092] µM | 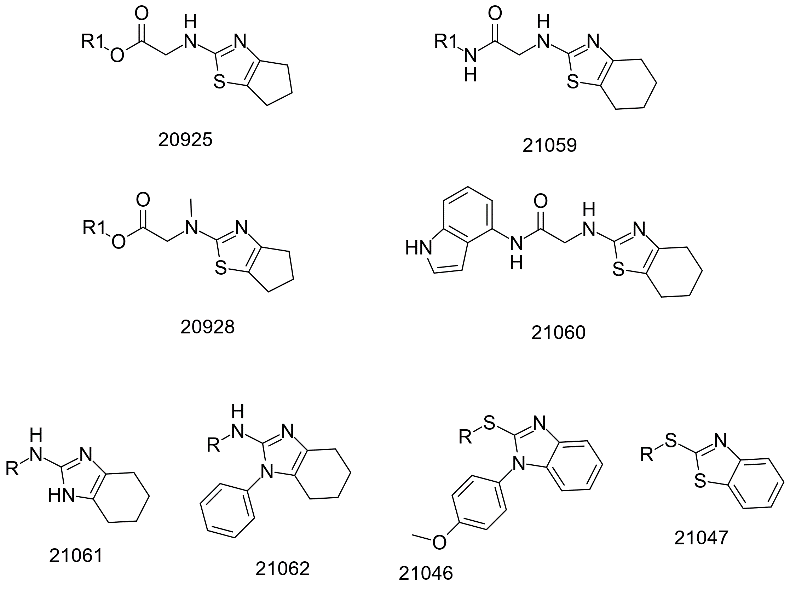 | 20925 | 0.87 [0.67-1.1] µM |
| 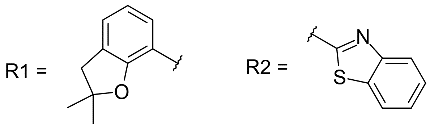 | | | | | |

Supplement: TABLE S4 [file mbio.02621-21-st004.docx]

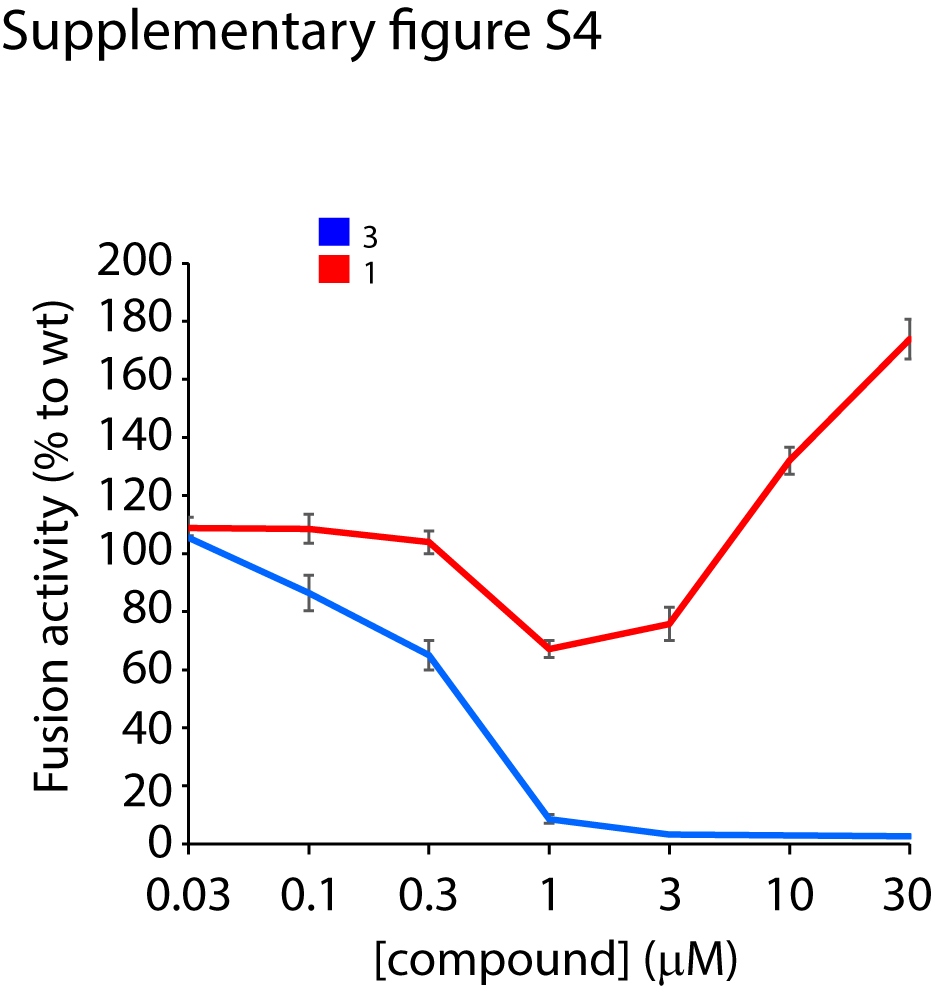

Supplement: FIG S4 [file mbio.02621-21-sf004.tif]
