# Supplementary material for: Highly Potent Host-Specific Small-Molecule Inhibitor of Paramyxovirus and Pneumovirus Replication with High Resistance Barrier
Source: mBio. 2021 Nov 2;12(6):e02621-21. doi: 10.1128/mBio.02621-21 (PMC8561388; doi:10.1128/mBio.02621-21)
Supplement: TABLE S5 [file mbio.02621-21-st005.docx]

| **Number in Manuscript** | **ZHAWOC** | **IC_50_ (CDV)** | **Structure** |
| --- | --- | --- | --- |
| **Purchased** | F2205-0189 | 0.53 [0.44-0.64] µM | 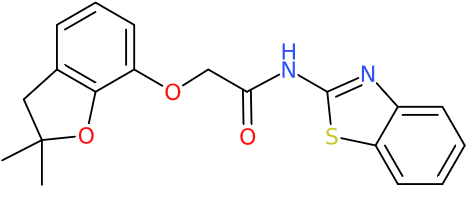 |
| **1** | 9045 | 0.52 [0.42-0.64] µM (n=15) | 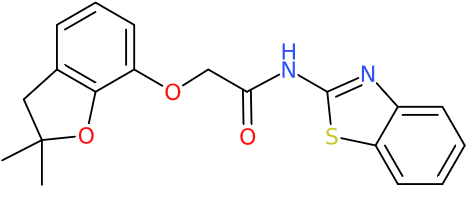 |
| **2** | 21026 | 0.0084 [0.0058-0.012] µM | 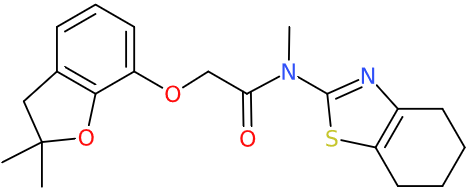 |
| **3** | 7613 (**3G**) | 26 [18-37] µM | 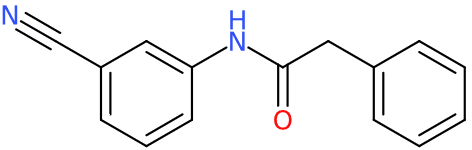 |
| **4** | 9047 | >100 µM | 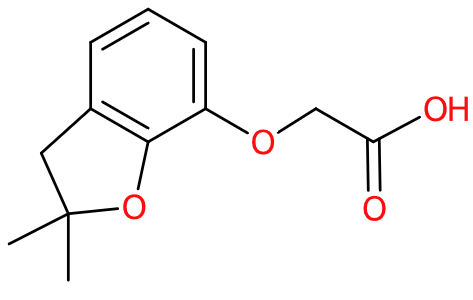 |
| **5** | 9215 | 0.73 [0.41-1.2] µM | 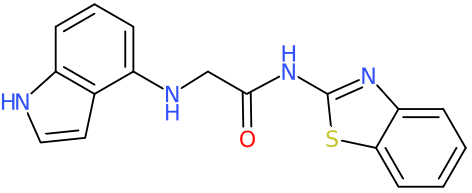 |
| **6** | 9150 | 91 [54-153] µM | 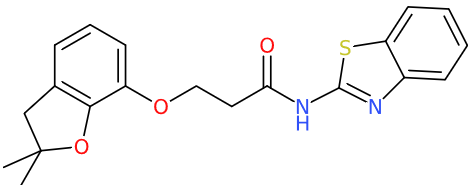 |
| **7** | 20343 | 1.7 [1.1-2.5] µM | 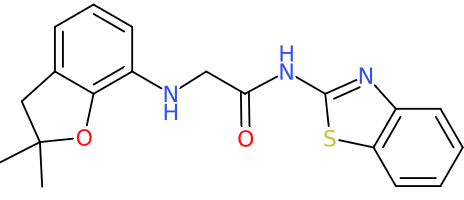 |
| **8** | 20575 | 0.35 [0.25-0.49] µM | 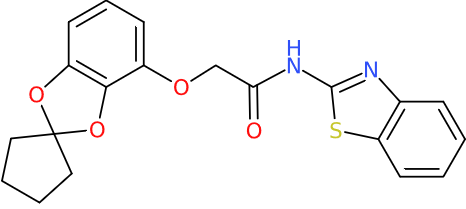 |
| **9** | 20255 | 0.40 [0.32-0.50] µM | 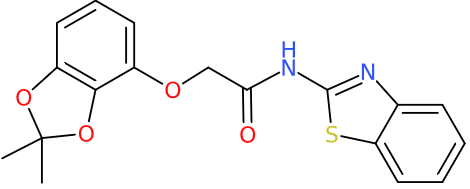 |
| **10** | 20574 | 0.11 [0.072-0.17] µM | 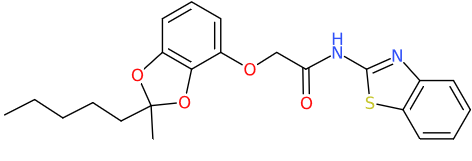 |
| **11** | 20819 | 39 [21-72] µM | 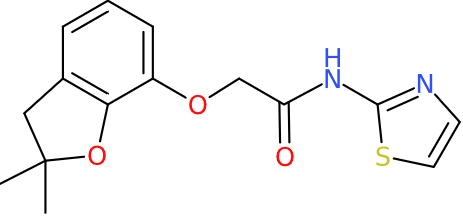 |
| **12** | 20607 | 2.2 [1.0-4.8] µM | 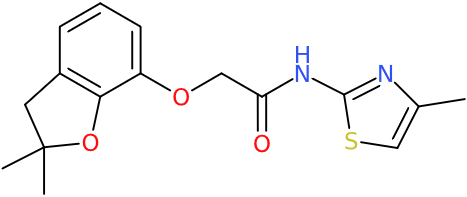 |
| **13** | 9128 | 0.86 [0.53-1.40] µM | 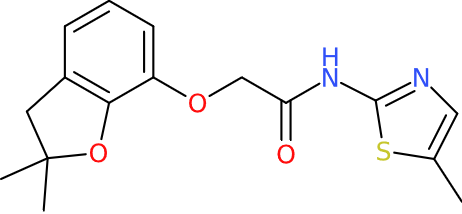 |
| **14** | 20314 | 0.13 [0.11-0.16] µM | 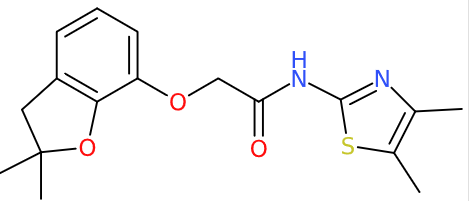 |
| **15** | 20459 | 0.16 [0.12-0.21] µM | 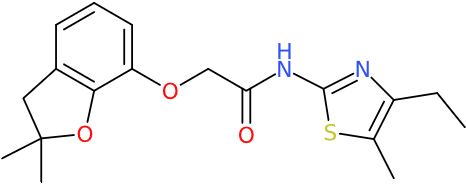 |
| **16** | 9129 | 0.084 [0.035-0.20] µM | 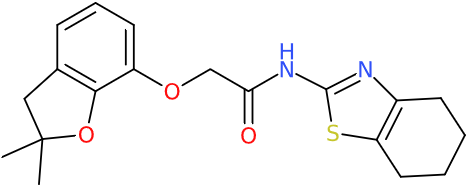 |
| **17** | 20458 | 0.056 [0.033-0.10] µM | 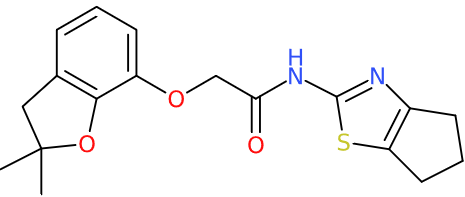 |
| **18** | 9172 | >100 µM | 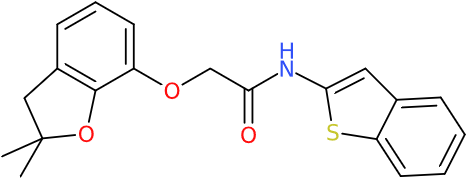 |
| **19** | 9048 | 44 [30-67] µM | 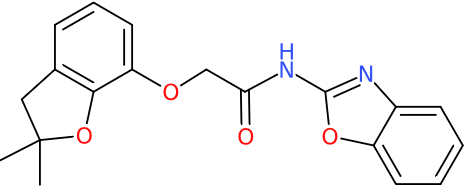 |
| **20** | 9053 | 67 [44-94] µM | 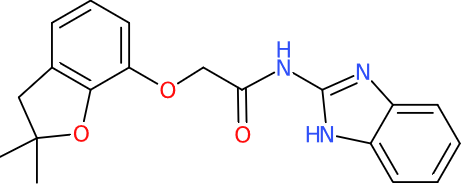 |
| **21** | 20327 | 0.34 [0.28-0.41] µM | 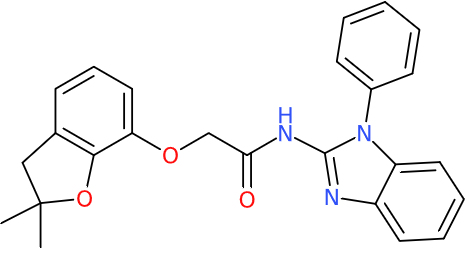 |
| **22** | 9213 | 0.16 [0.13-0.19] µM | 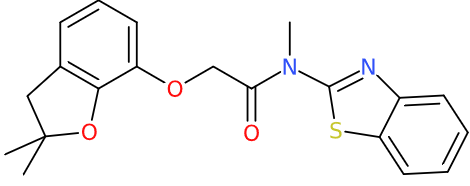 |
| **23** | 20269 | 0.27 [0.22-0.31] µM | 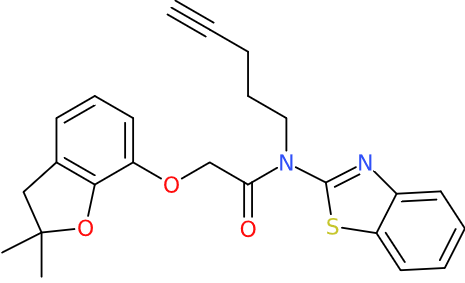 |
| **24** | 20277 | 0.25 [0.20-0.32] µM | 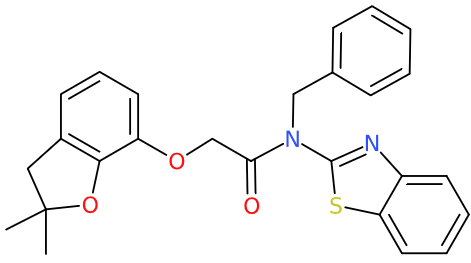 |
| **25** | 21048 | 0.080 [0.069-0.091] µM | 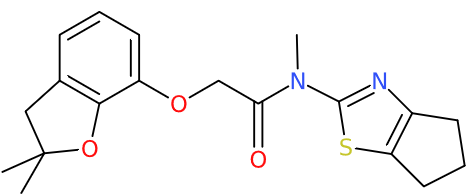 |
| **26** | 21027 | 0.0041 [0.0026-0.0066] µM | 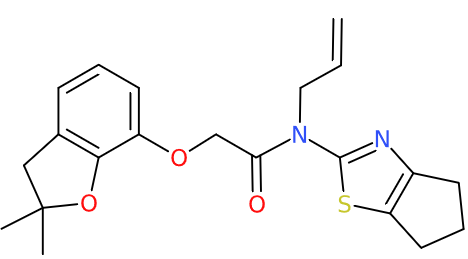 |
| **27** | 20926 | 0.0082 [0.0062-0.011] µM | 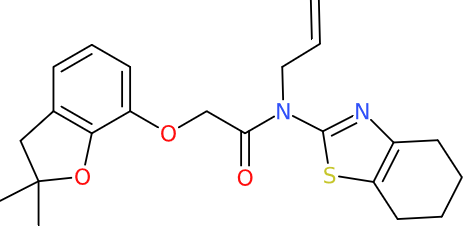 |
| **28** | 20463 | 0.26 [0.18-0.38] µM | 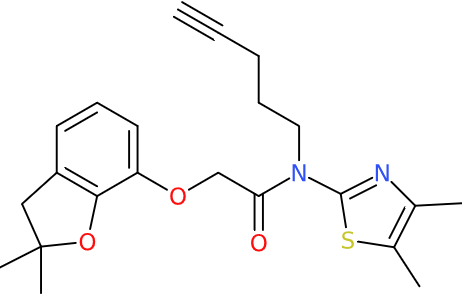 |
| **29** | 20460 | 1.7 [0.85-3.4] µM | 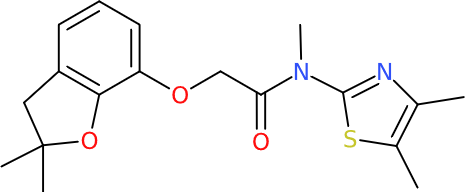 |
| **30** | 9147 | 0.48 [0.37-0.63] µM | 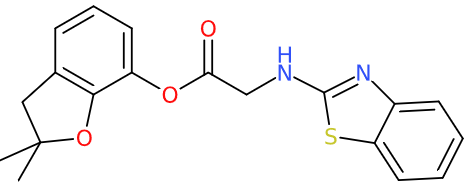 |
| **31** | 20928 | 0.076 [0.060-0.092] µM | 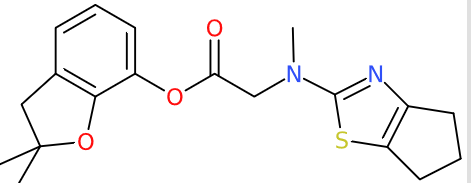 |
| **32** | 20330 | 53 [21-142] µM | 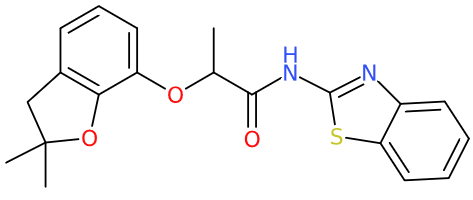 |
| **33** | 9154 | >100 µM | 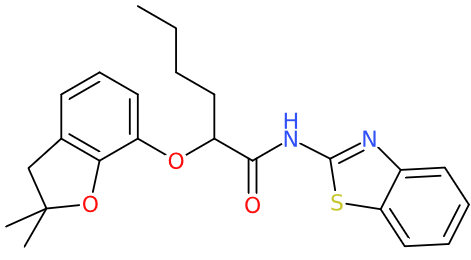 |
| **34** | 9173 | >100 µM | 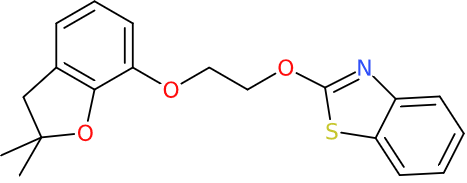 |
| **35** | 9152 | >100 µM | 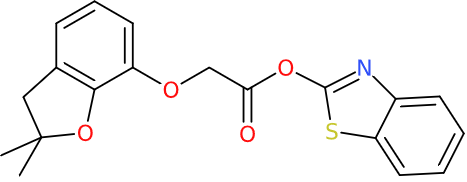 |
| **36** | 20626 | >100 µM | 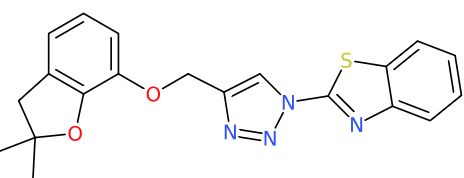 |
| **37** | 20455 | 59 [26-142] µM | 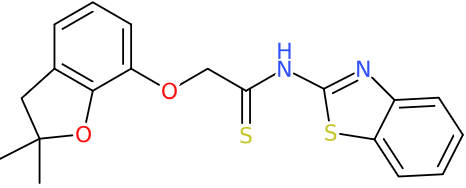 |
| **38** | 9148 | 85 [42-172] µM | 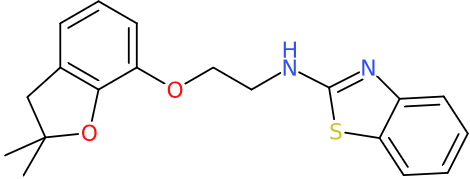 |
| **39** | 20925 | 0.87 [0.67-1.12] µM | 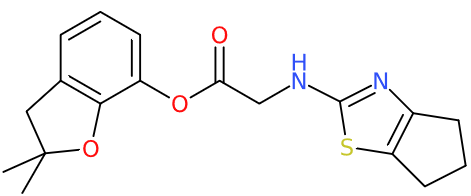 |
|  | 9050 | 8.3 [6.3-11] µM | 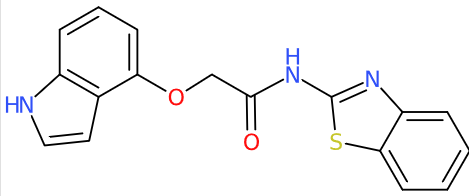 |
|  | 9051 | >100 µM | 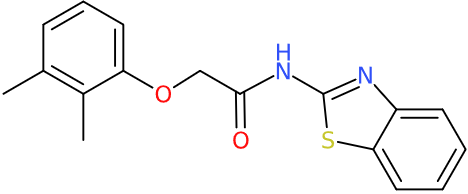 |
|  | 9052 | >100 µM | 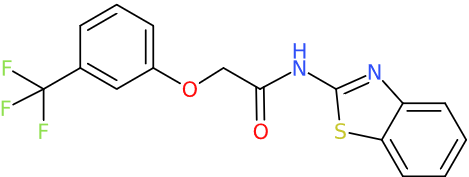 |
|  | 9054 | 37 [30-52] µM | 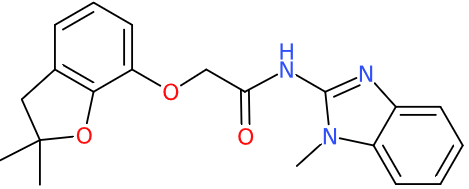 |
|  | 9055 | 48 [38-71] µM | 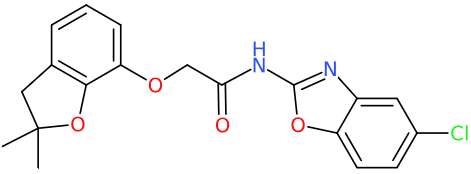 |
|  | 9056 | 57 [39-81] µM | 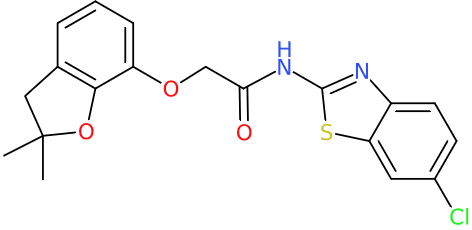 |
|  | 9057 | >100 µM | 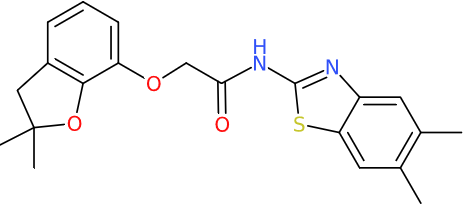 |
|  | 9058 | 83 [54-135] µM | 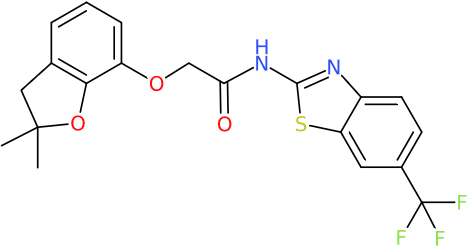 |
|  | 9059 | 3.4 [2.8-4.1] µM | 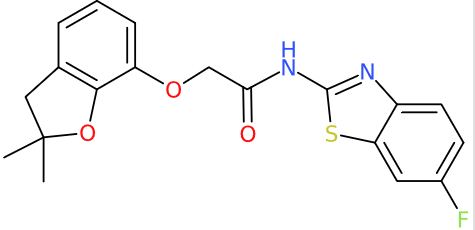 |
|  | 9114 | 47 [16-98] µM | 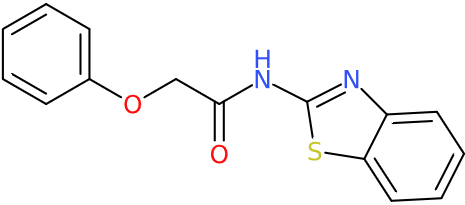 |
|  | 9115 | >100 µM | 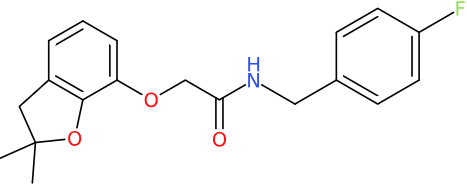 |
|  | 9116 | >100 µM | 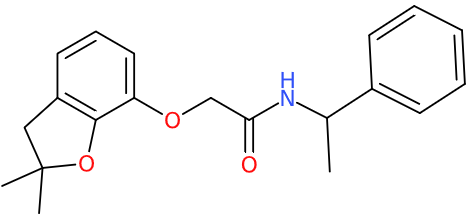 |
|  | 9117 | >100 µM | 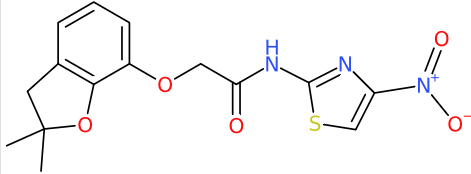 |
|  | 9118 | >100 µM | 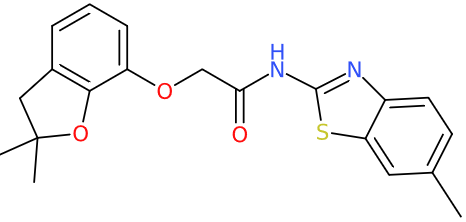 |
|  | 9119 | >100 µM | 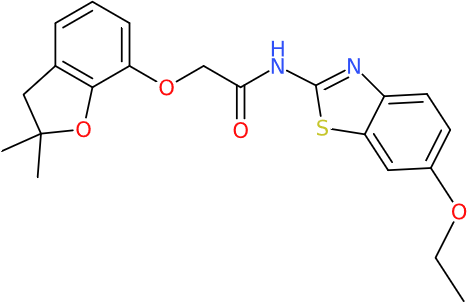 |
|  | 9120 | >100 µM | 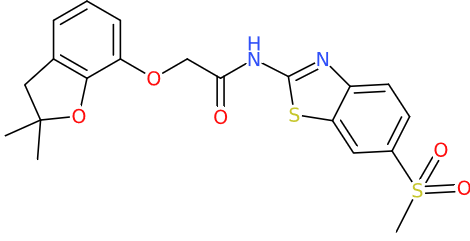 |
|  | 9121 | 40 [17-71] µM | 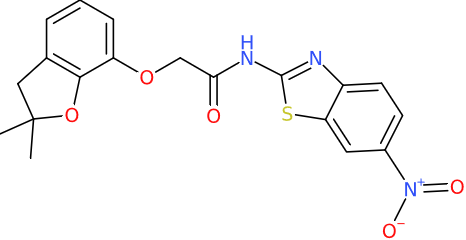 |
|  | 9122 | >100 µM | 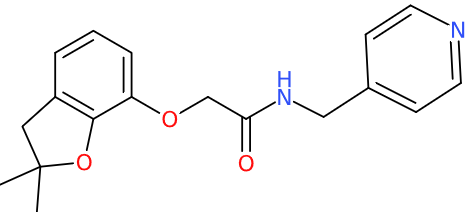 |
|  | 9123 | 85 [34-210] µM | 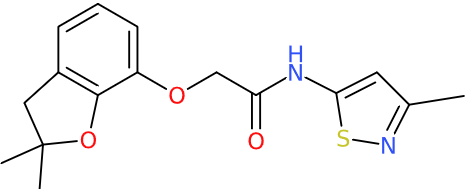 |
|  | 9124 | >100 µM | 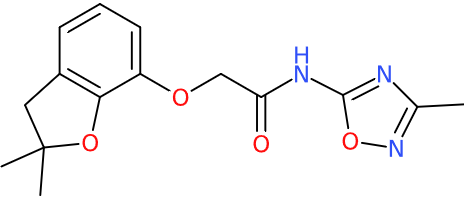 |
|  | 9125 | >100 µM | 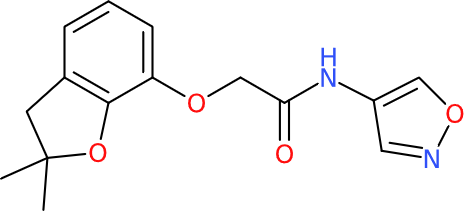 |
|  | 9126 | 26 [12-51] µM | 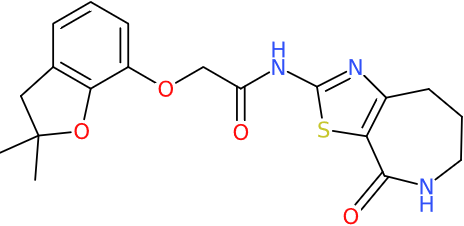 |
|  | 9127 | >100 µM | 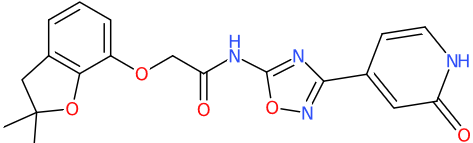 |
|  | 9149 | 26 [10-67] µM | 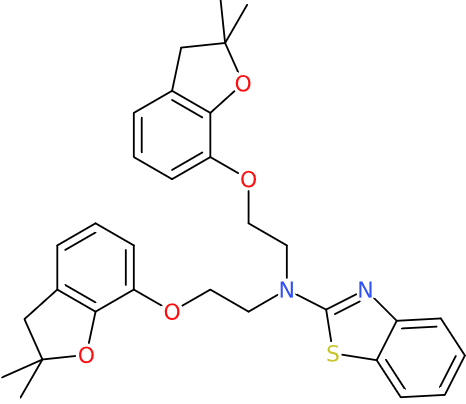 |
|  | 9168 | >100 µM | 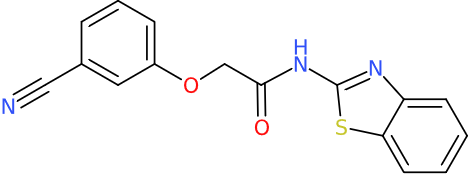 |
|  | 9169 | >100 µM | 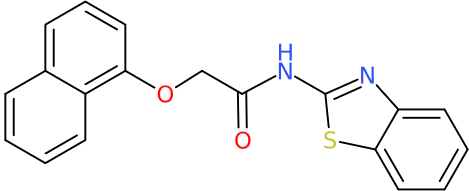 |
|  | 9171 | >100 µM | 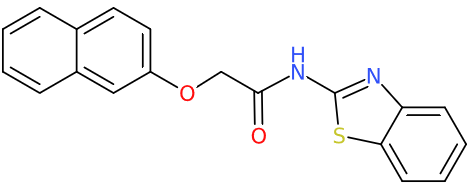 |
|  | 9214 | 63 [45-93] µM | 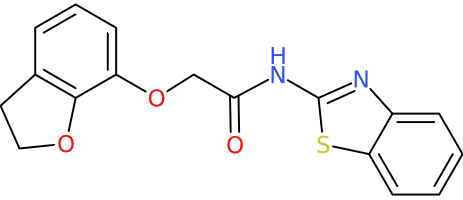 |
|  | 20256 | >100 µM | 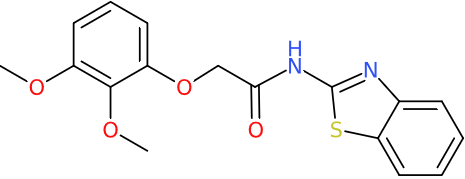 |
|  | 20257 | 48 [31-77] µM | 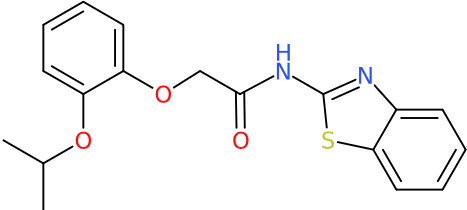 |
|  | 20258 | >100 µM | 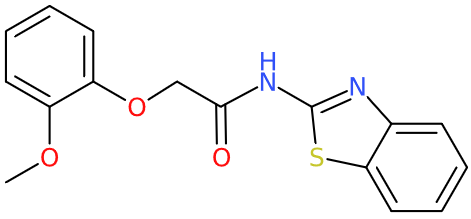 |
|  | 20278 | 34 [1.4-163] µM | 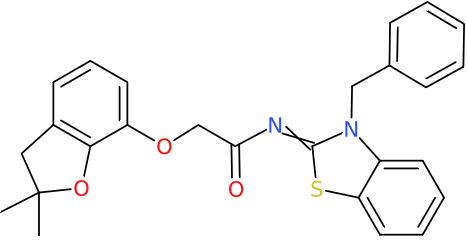 |
|  | 20320 | 56 [20-165] µM | 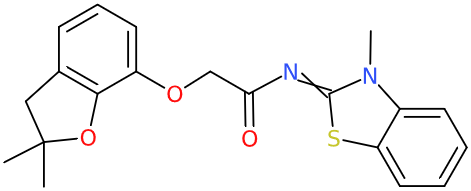 |
|  | 20326 | 64 [28-146] µM | 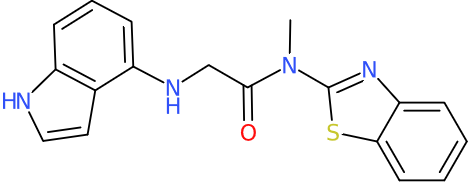 |
|  | 20328 | 30 [6.9-79] µM | 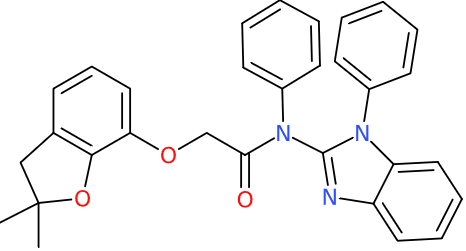 |
|  | 20331 | 64 [38-107] µM | 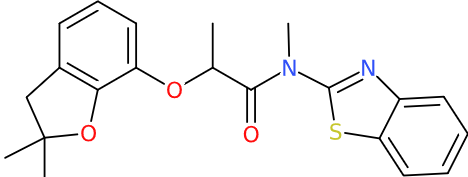 |
|  | 20608 | 1.1 [0.59-2.1] µM | 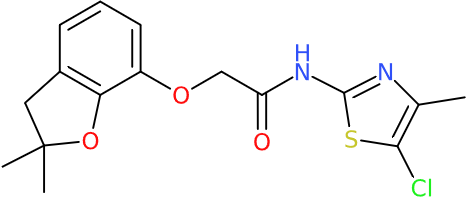 |
|  | 20836 | 0.93 [0.53-1.3] µM | 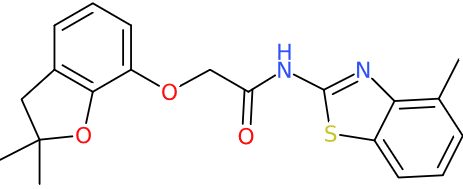 |
|  | 20837 | >100 µM | 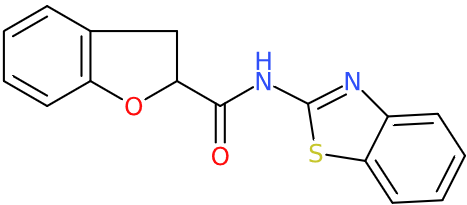 |
|  | 20838 | >100 µM | 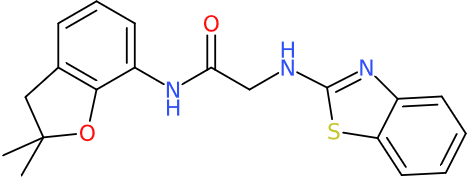 |
|  | 20839 | 44 [12 - 89] µM | 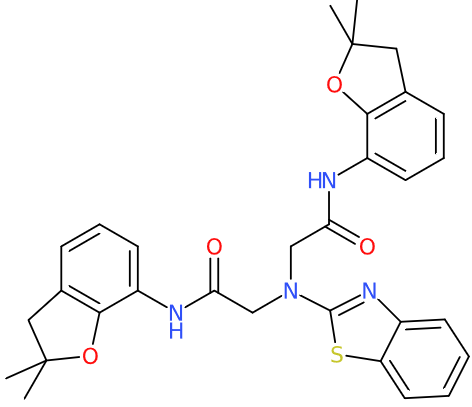 |
|  | 20840 | >100 µM | 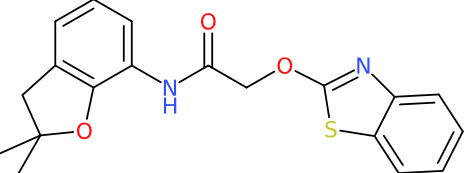 |
|  | 20927 | 29 [20.7-46.8] µM | 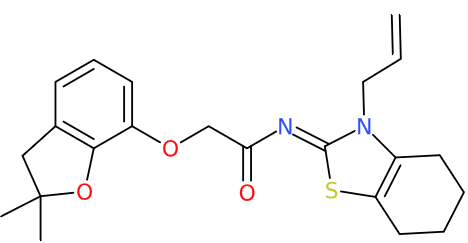 |
|  | 21057 | >100 µM | 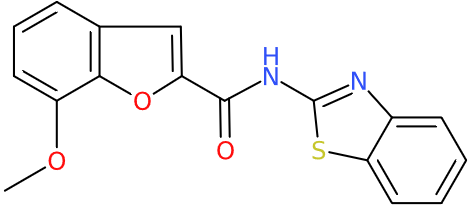 |
