# Supplementary material for: Highly Potent Host-Specific Small-Molecule Inhibitor of Paramyxovirus and Pneumovirus Replication with High Resistance Barrier
Source: mBio. 2021 Nov 2;12(6):e02621-21. doi: 10.1128/mBio.02621-21 (PMC8561388; doi:10.1128/mBio.02621-21)
Supplement: TABLE S2 [file mbio.02621-21-st002.docx]

| **Benzothiazole derivatives** | | | | | | |
| --- | --- | --- | --- | --- | --- | --- |
| **Residue** | **ID** | | **IC_50_** | **Residue** | **ID** | **IC_50_** |
| 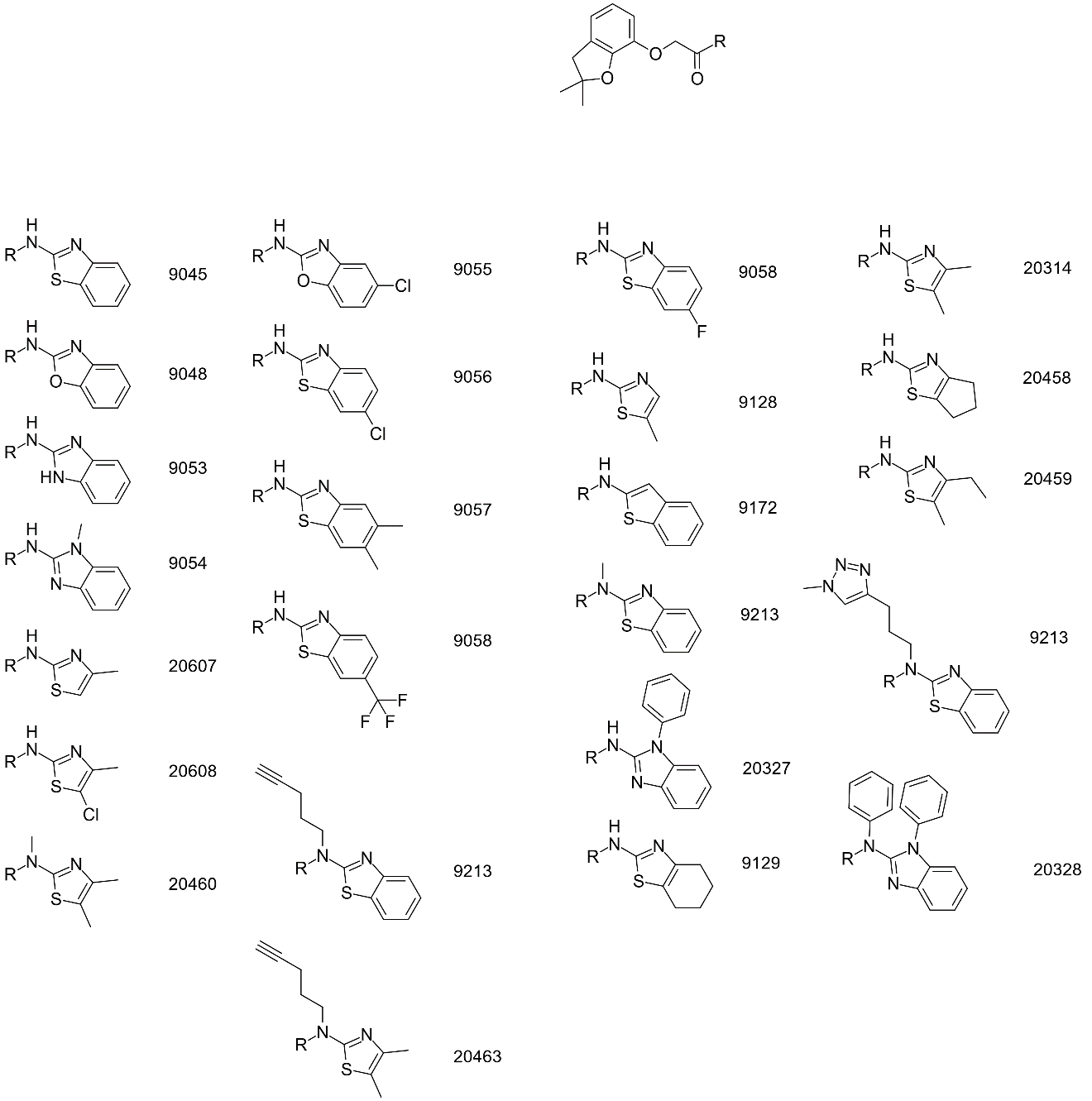 | 9045 | | 0.52 [0.42-0.64] µM | 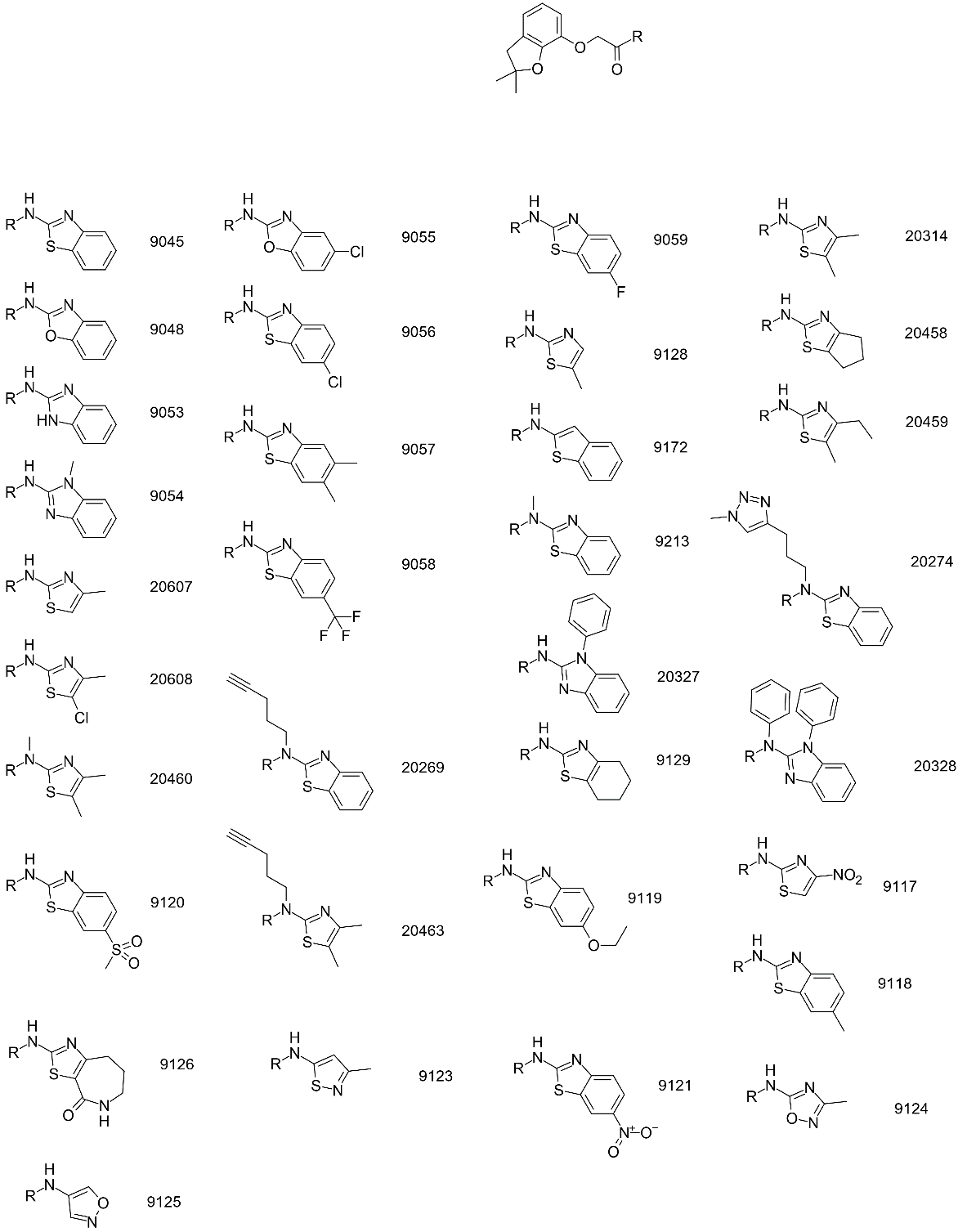 | 9119 | >100 µM |
| 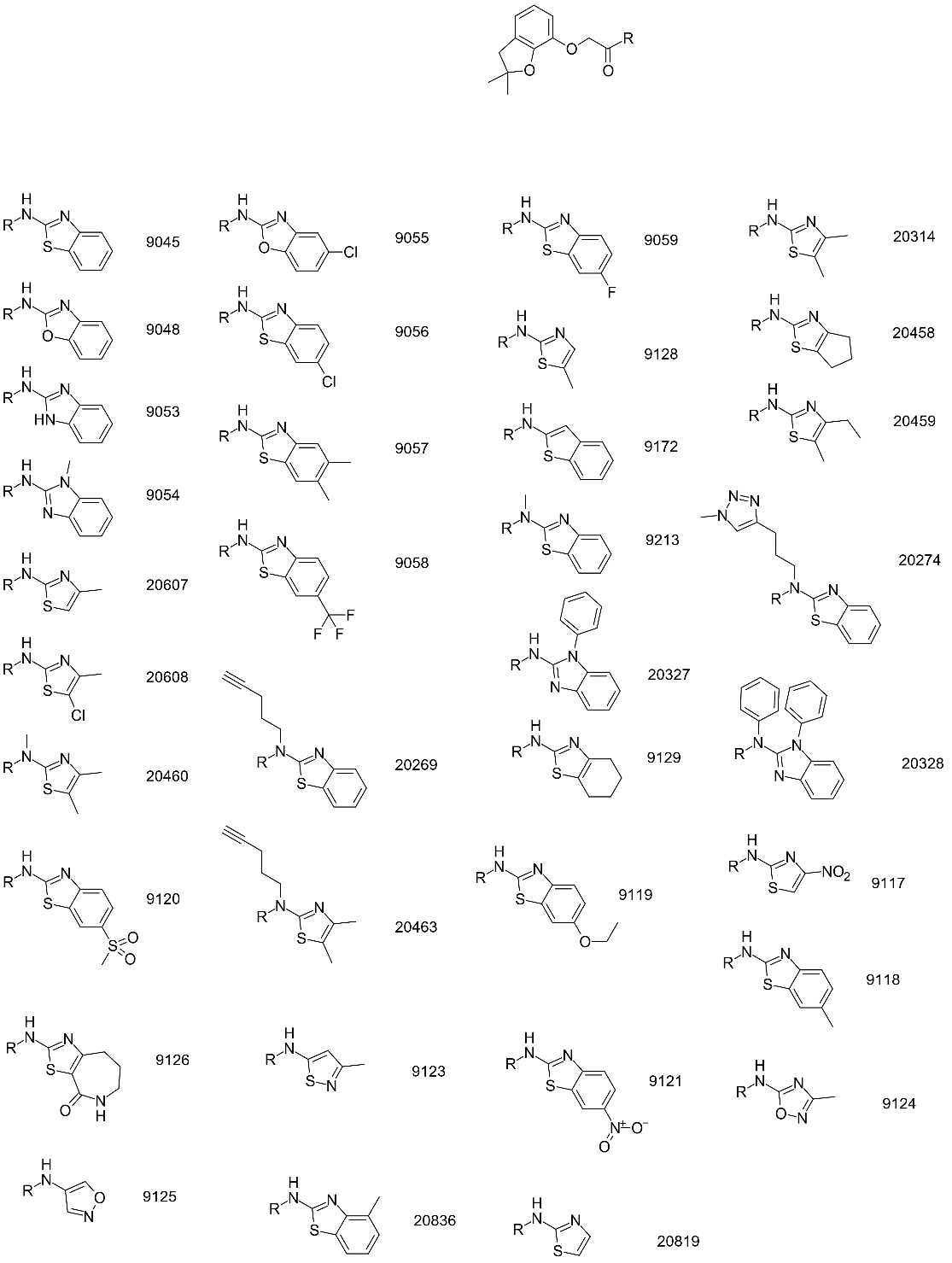 | 20836 | | 0.93 [0.53-1.3] µM | 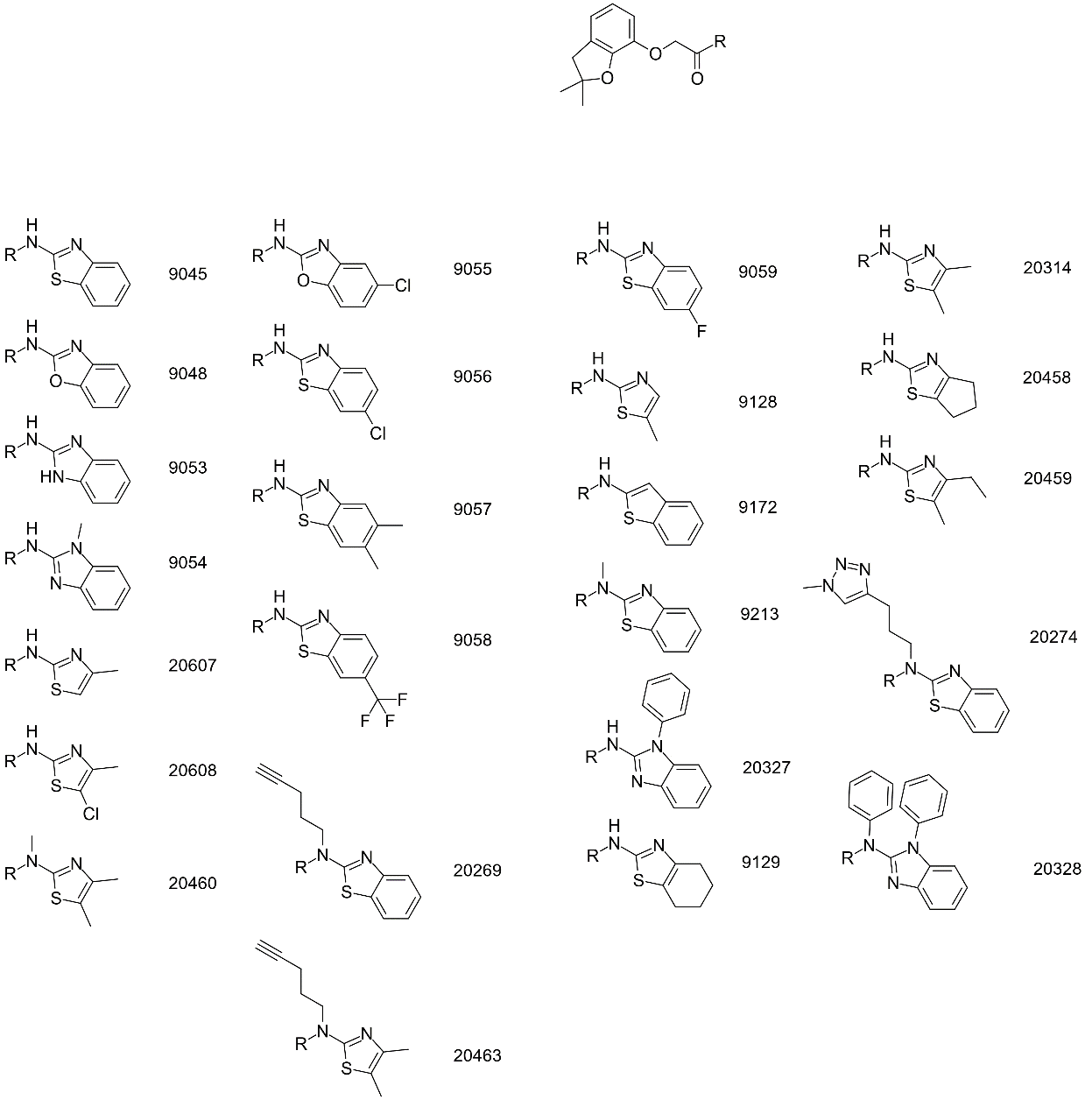 | 9057 | >100 µM |
| 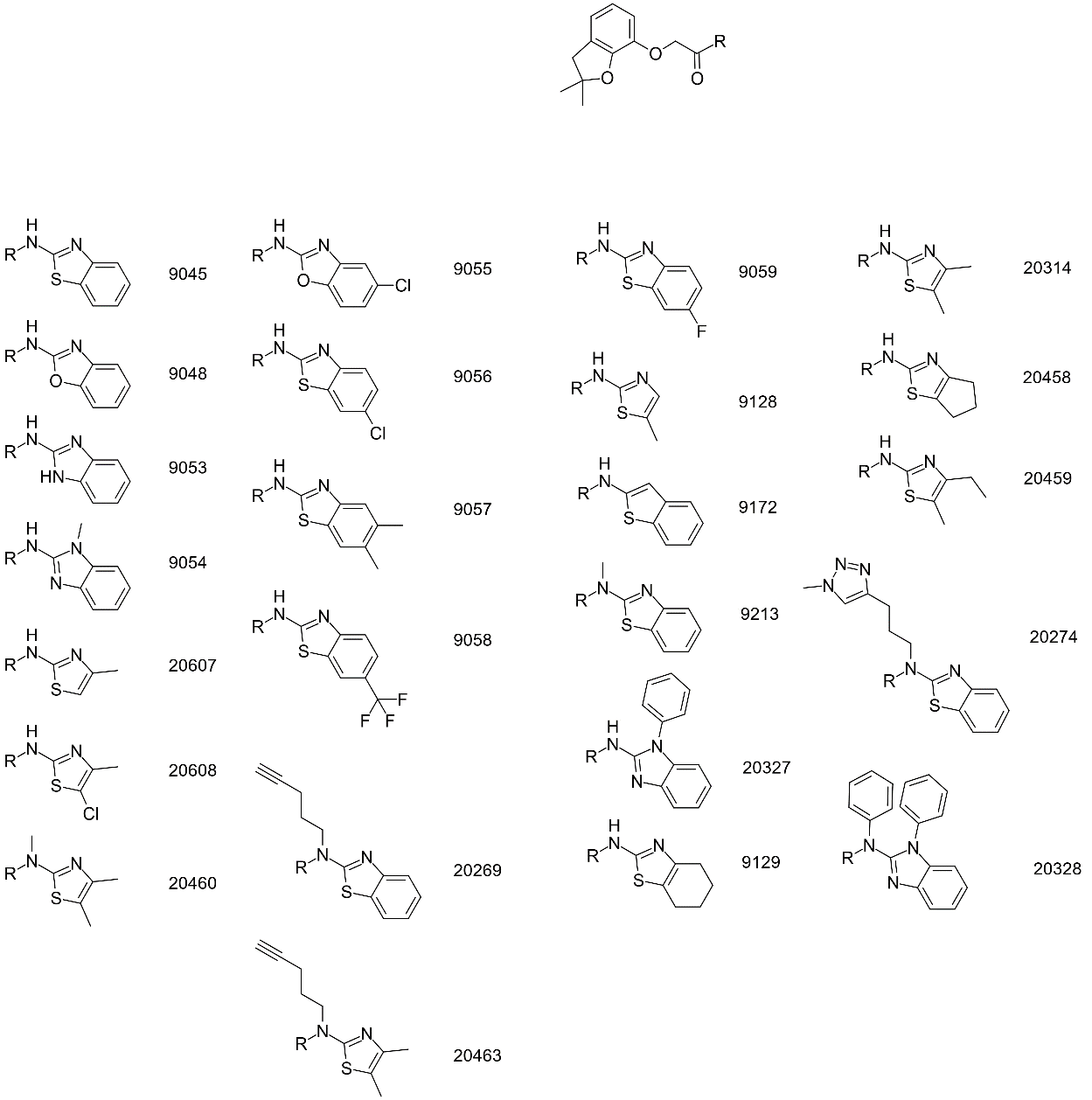 | 9059 | | 3.4 [2.8-4.1] µM | 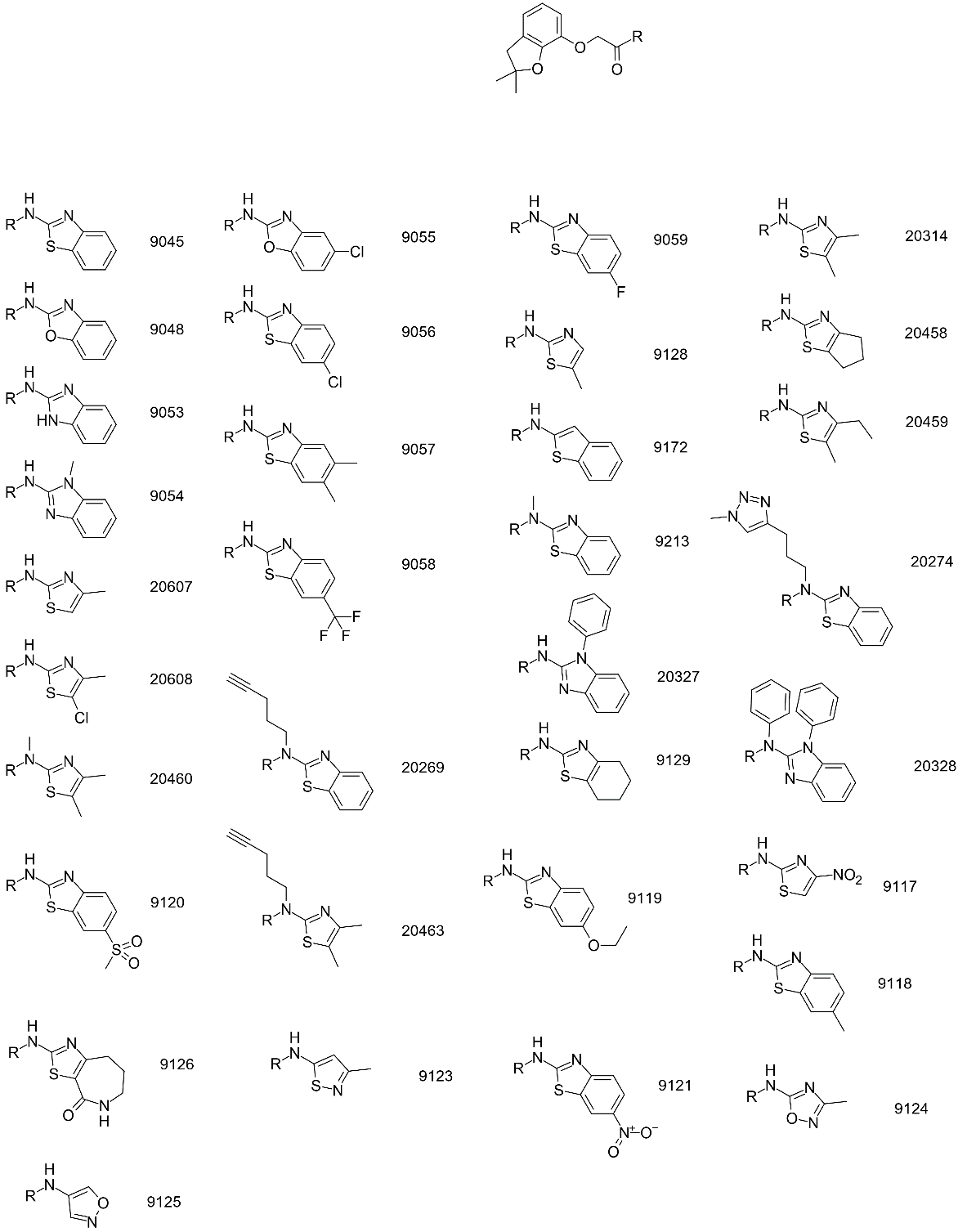 | 9120 | >100 µM |
| 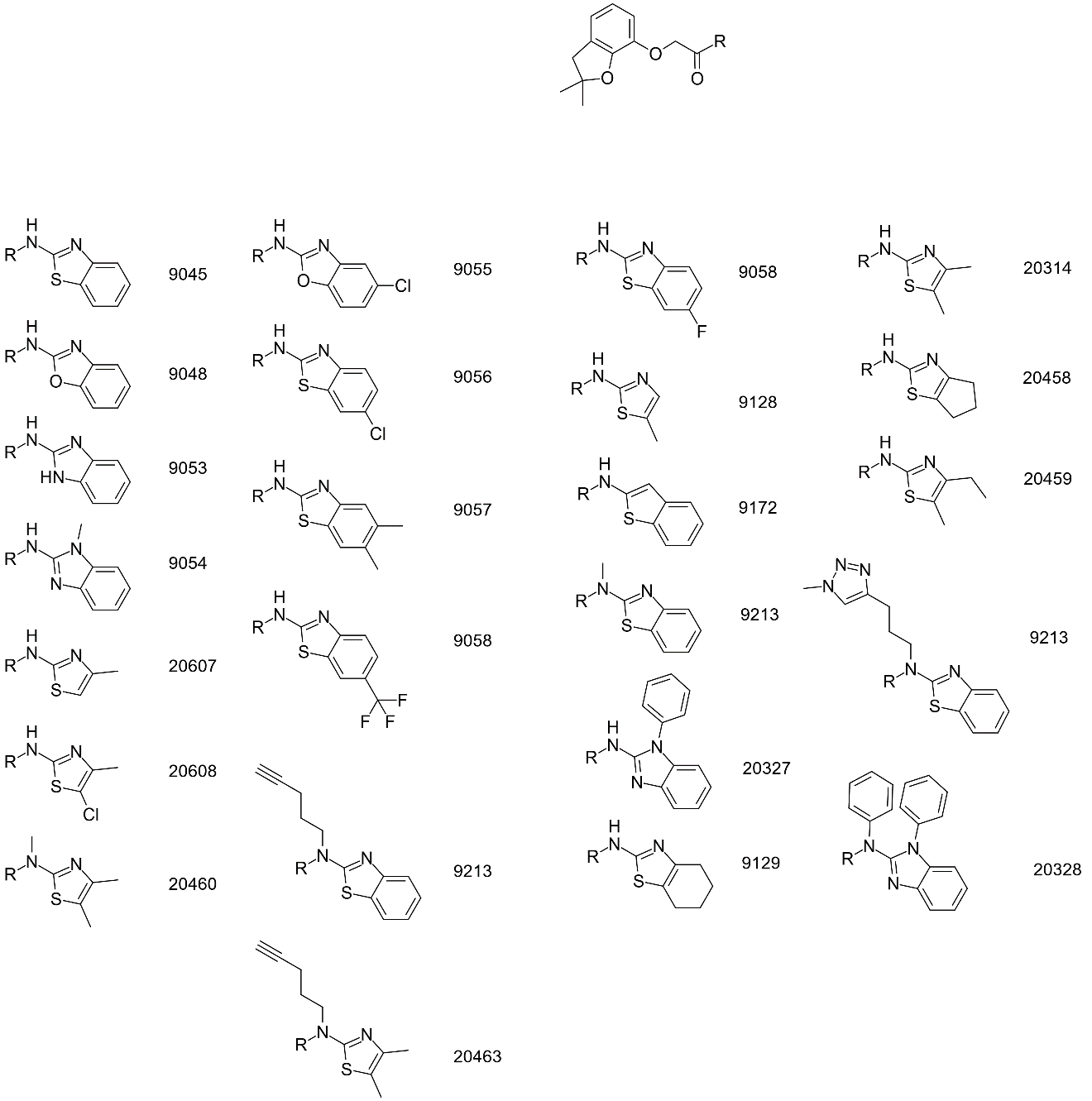 | 9056 | | 57 [39-81] µM | 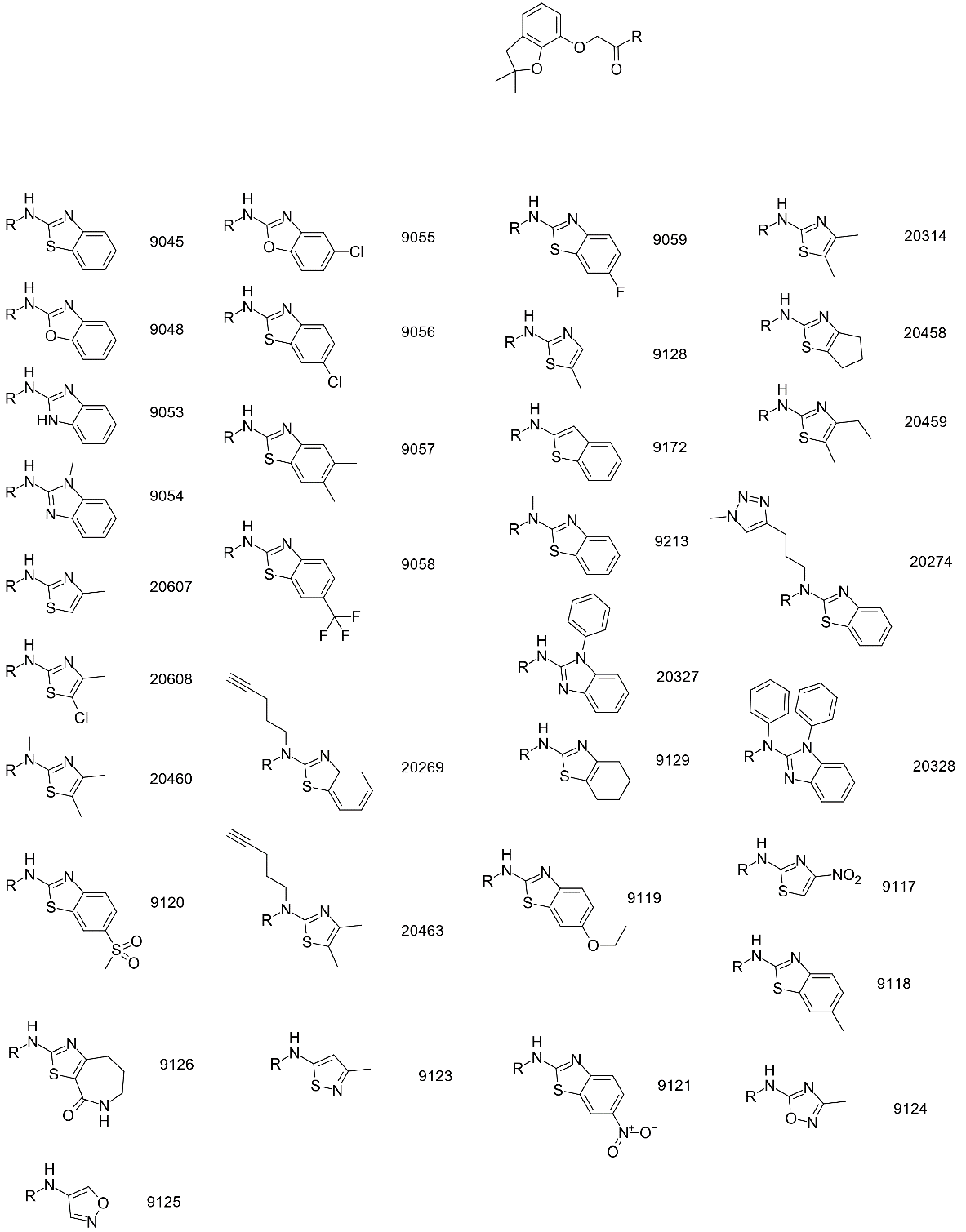 | 9121 | >100 µM |
| 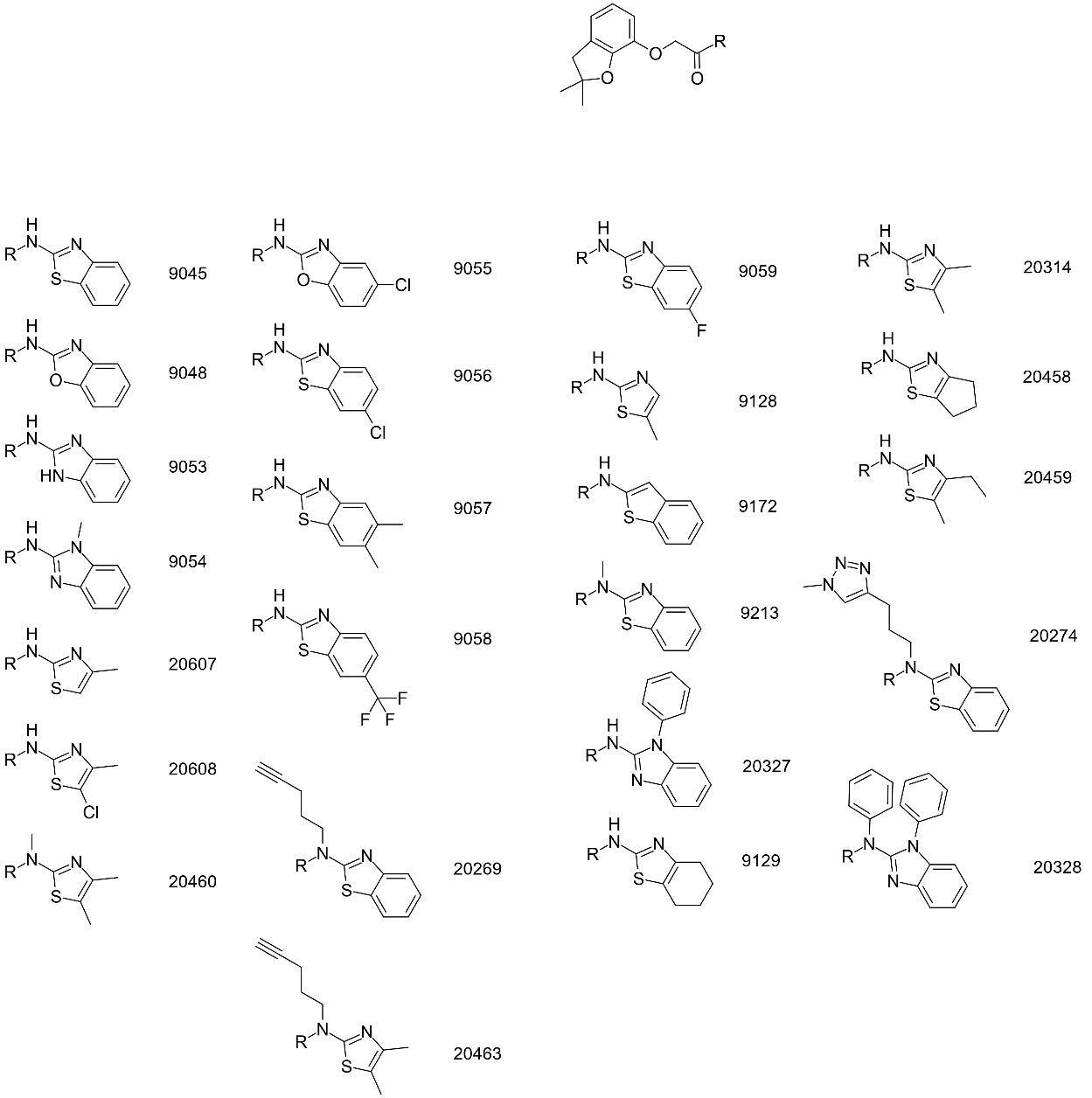 | 9058 | | 83 [54-135] µM | 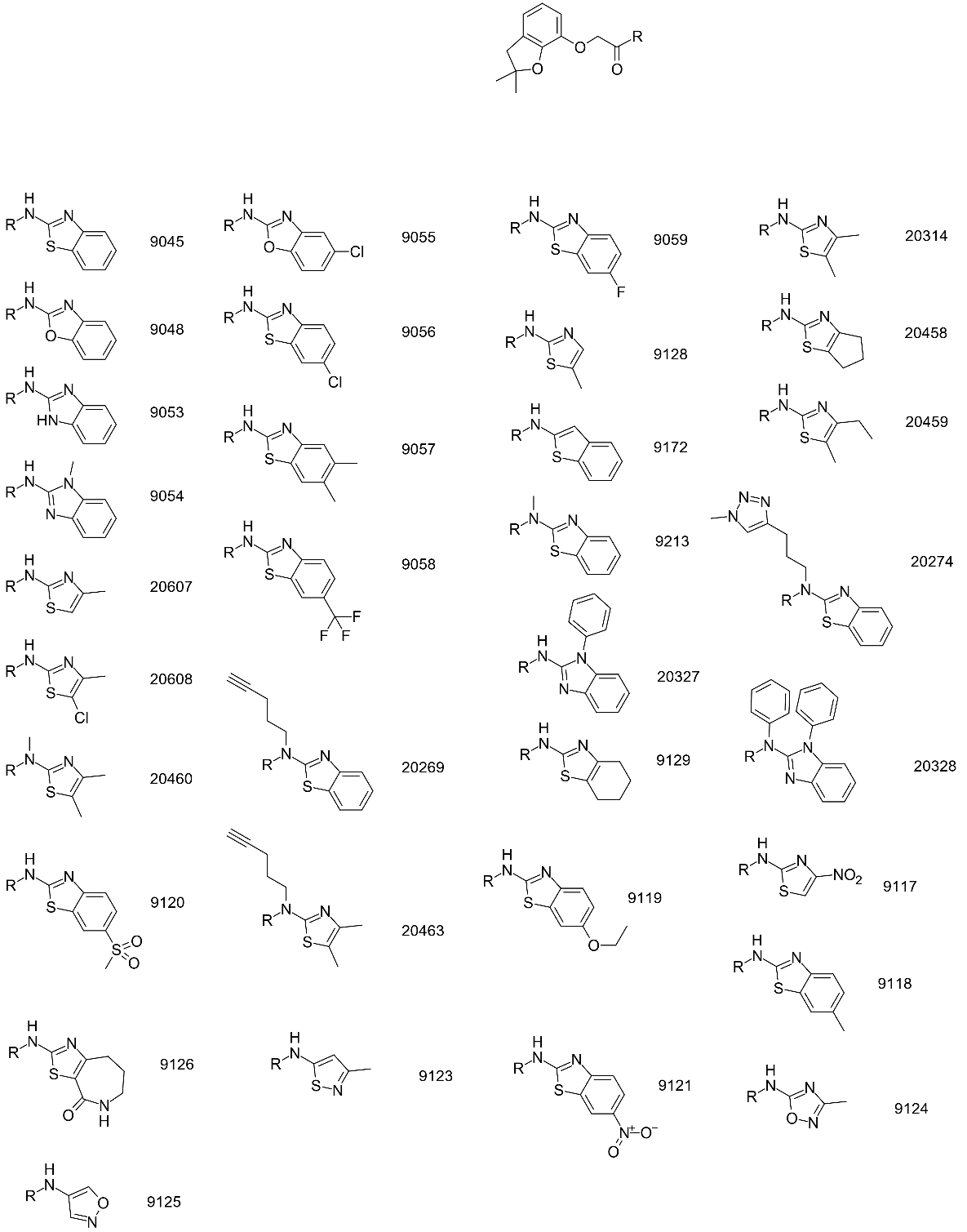 | 9118 | >100 µM |
|  |  | |  |  |  |  |
| **Thiazole derivatives** | | | | | | |
| **Residue** | **ID** | | **IC_50_** | **Residue** | **ID** | **IC_50_** |
| 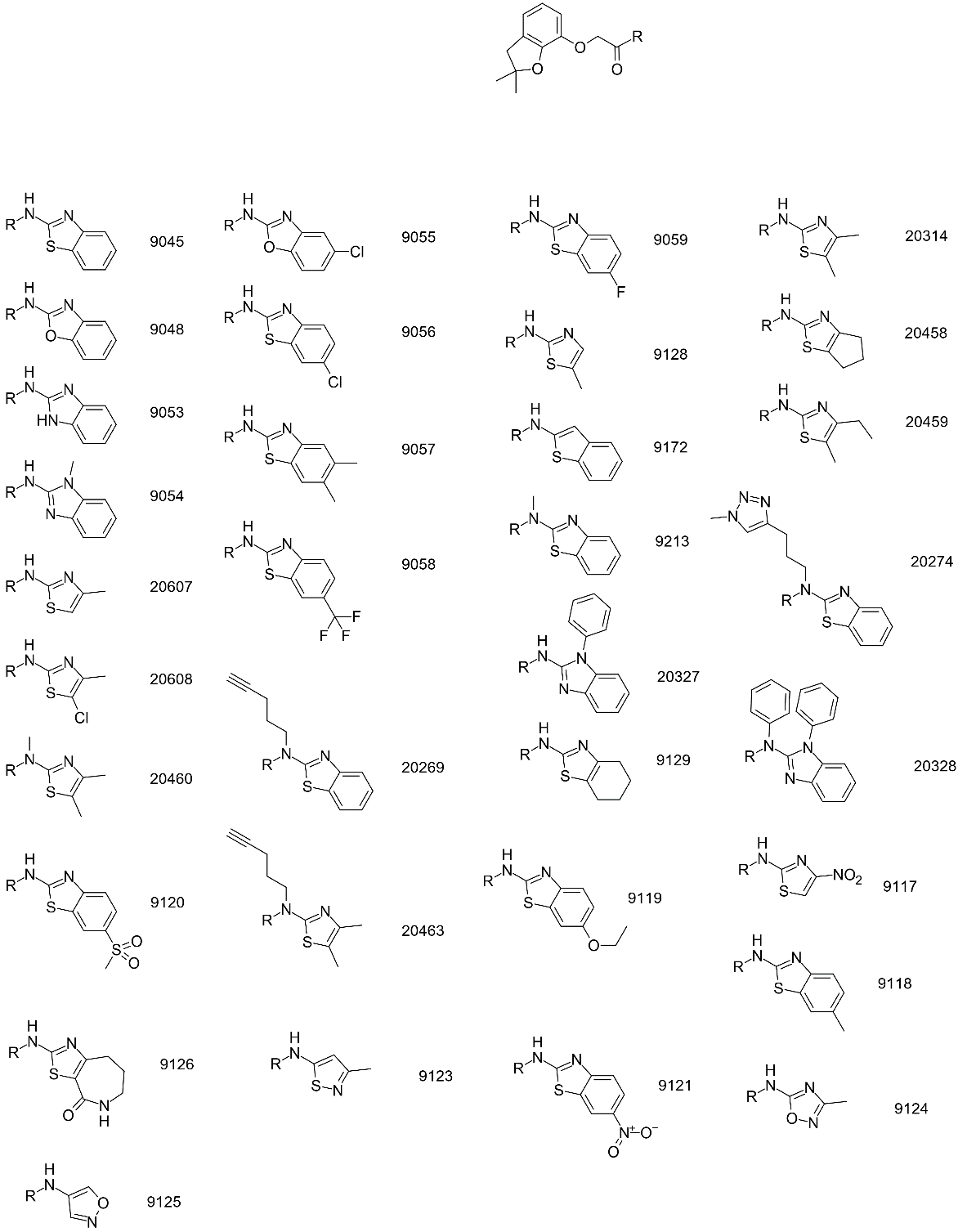 | 20458 | | 0.056 [0.033-0.10] µM | 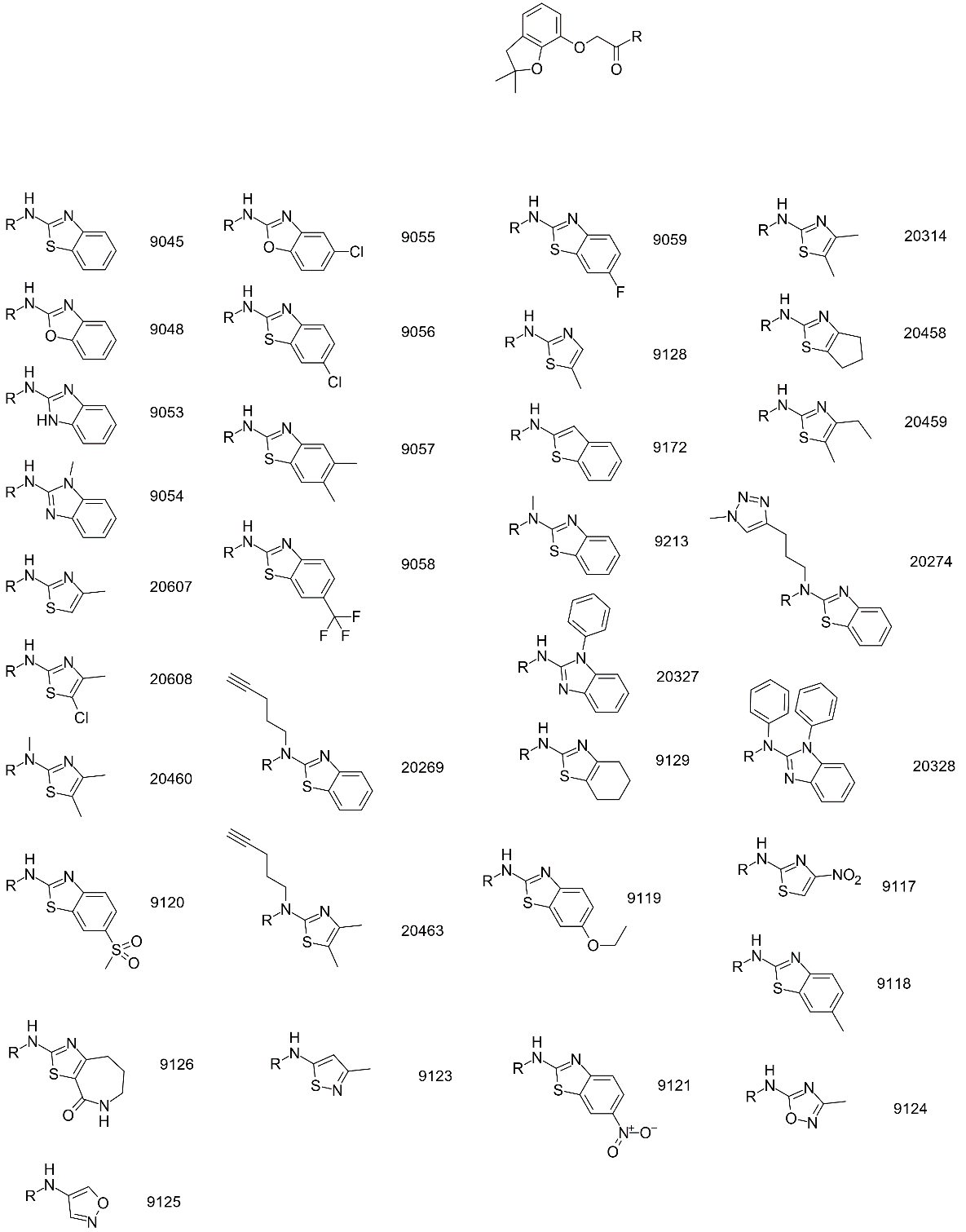 | 20608 | 1.1 [0.59-2.1] µM |
| 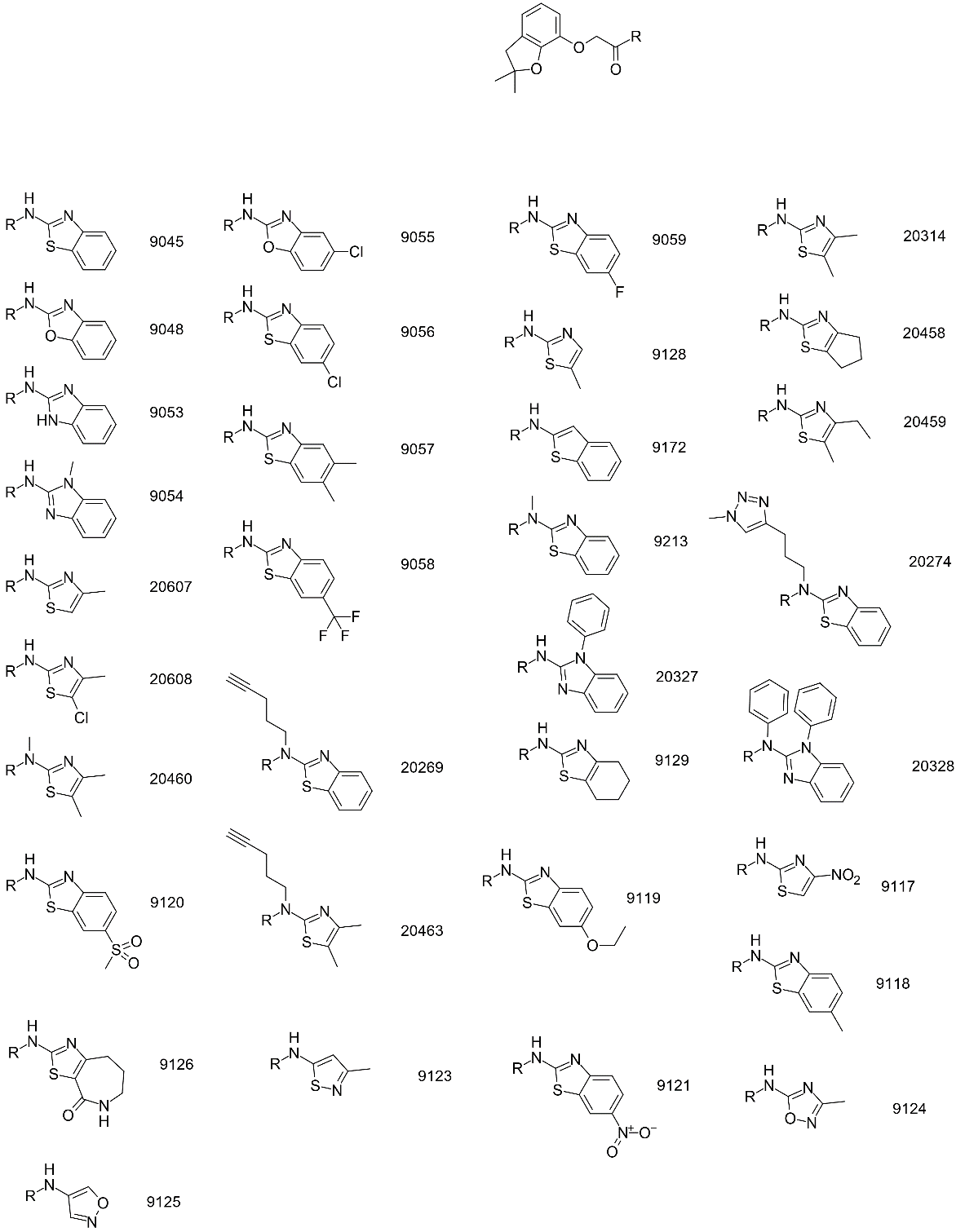 | 9129 | | 0.084 [0.035-0.20] µM | 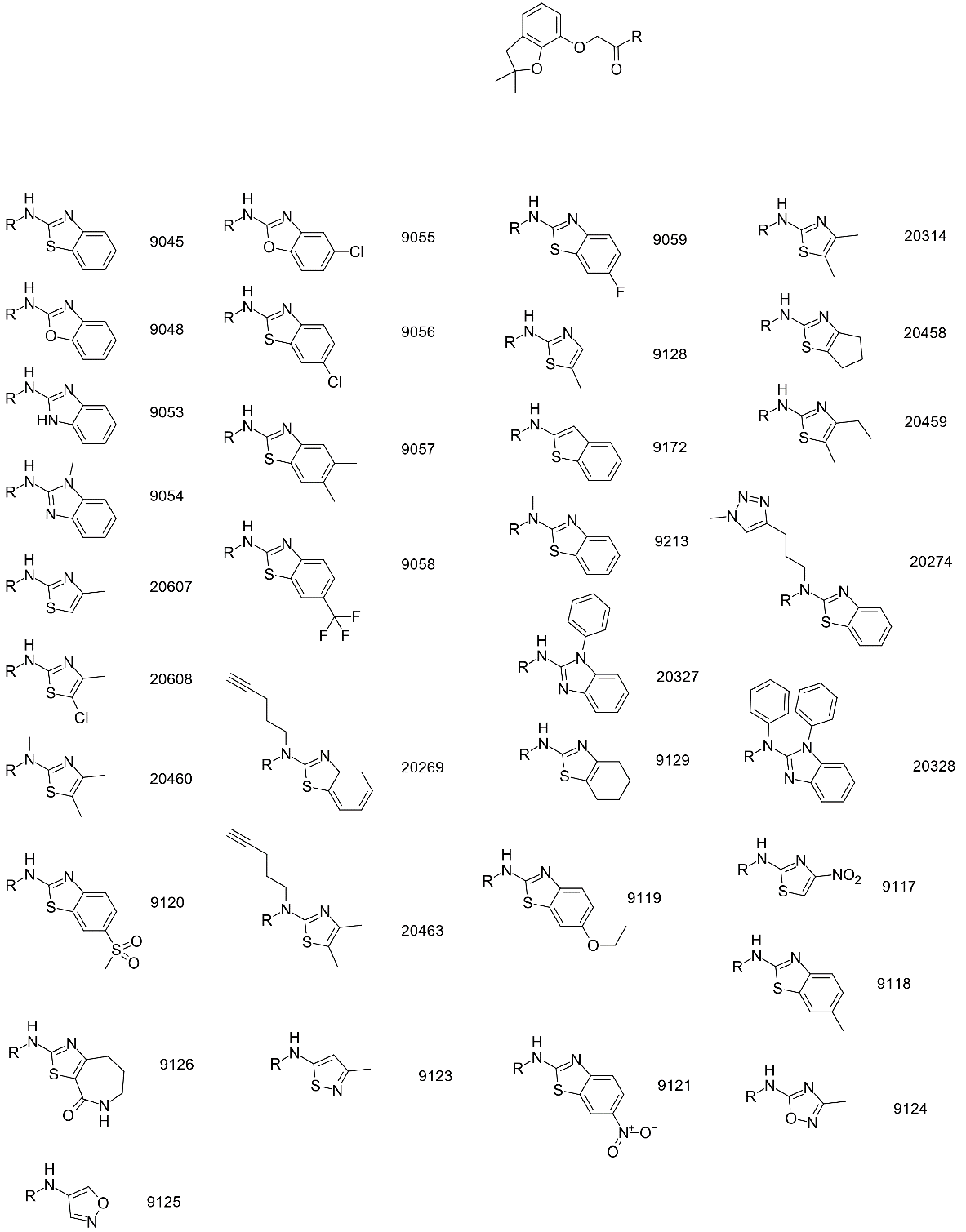 | 20607 | 2.2 [1.0-4.8] µM |
| 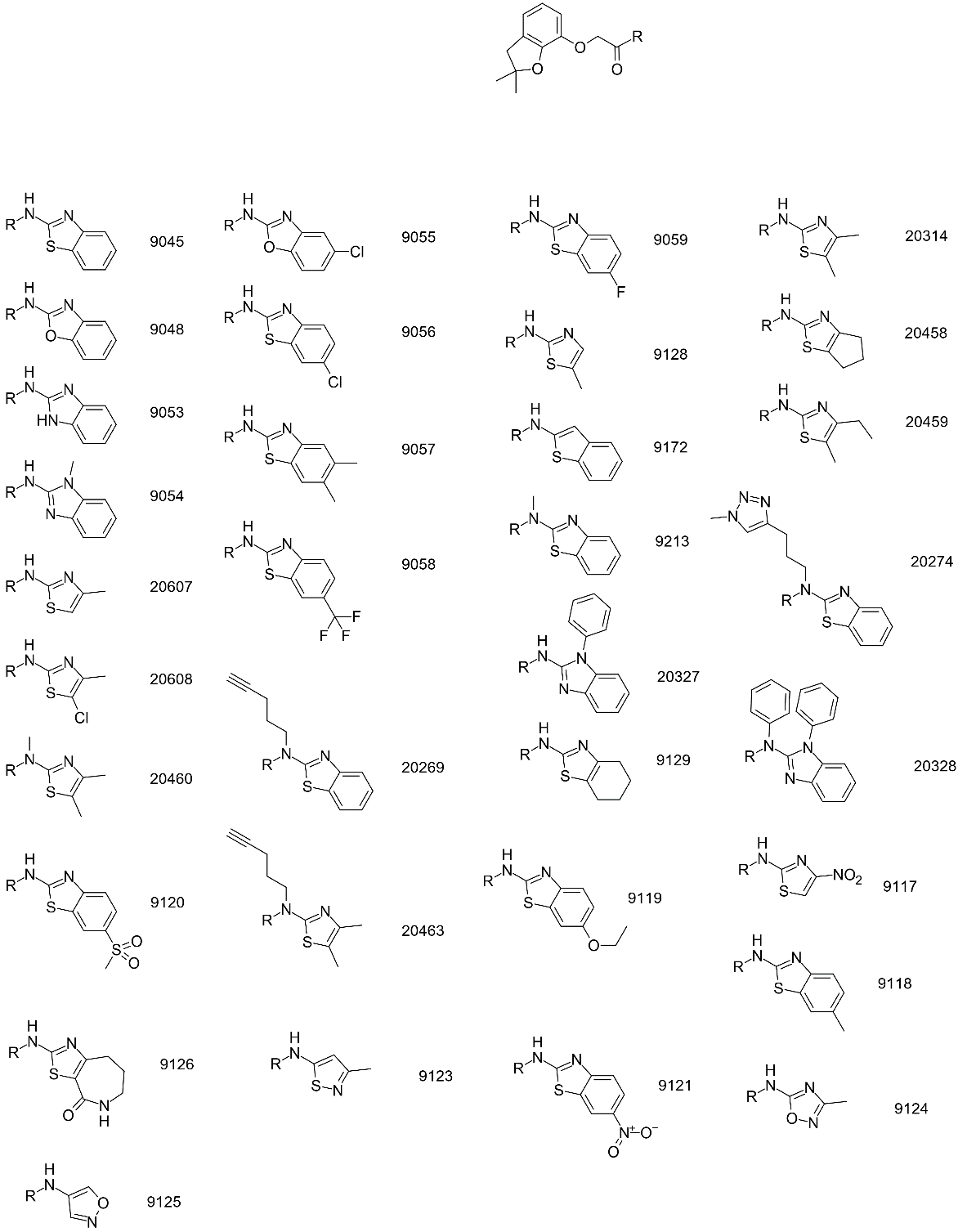 | 20314 | | 0.13 [0.11-0.16] µM | 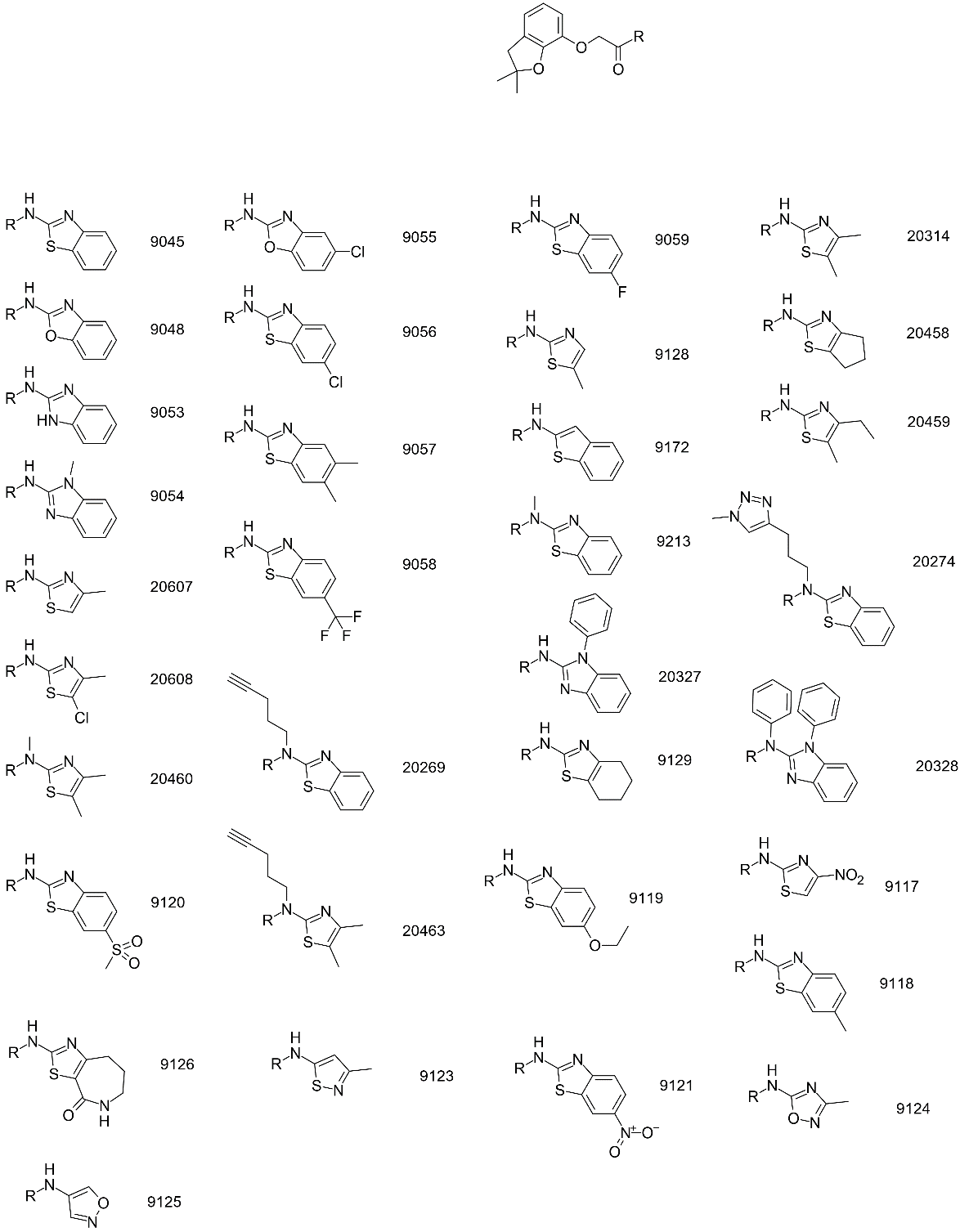 | 9126 | 26 [12-51] µM |
| 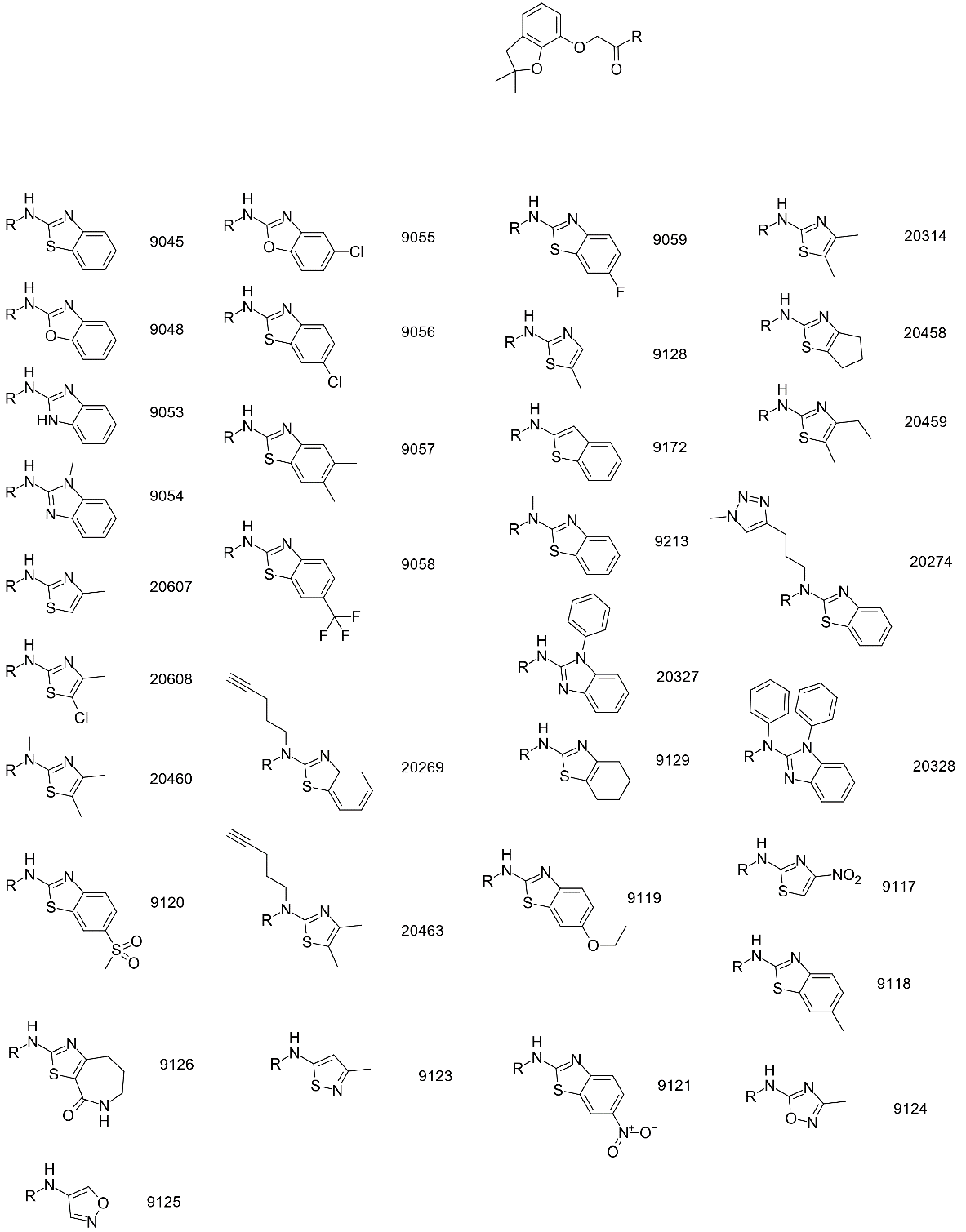 | 20459 | | 0.16 [0.12-0.21] µM | 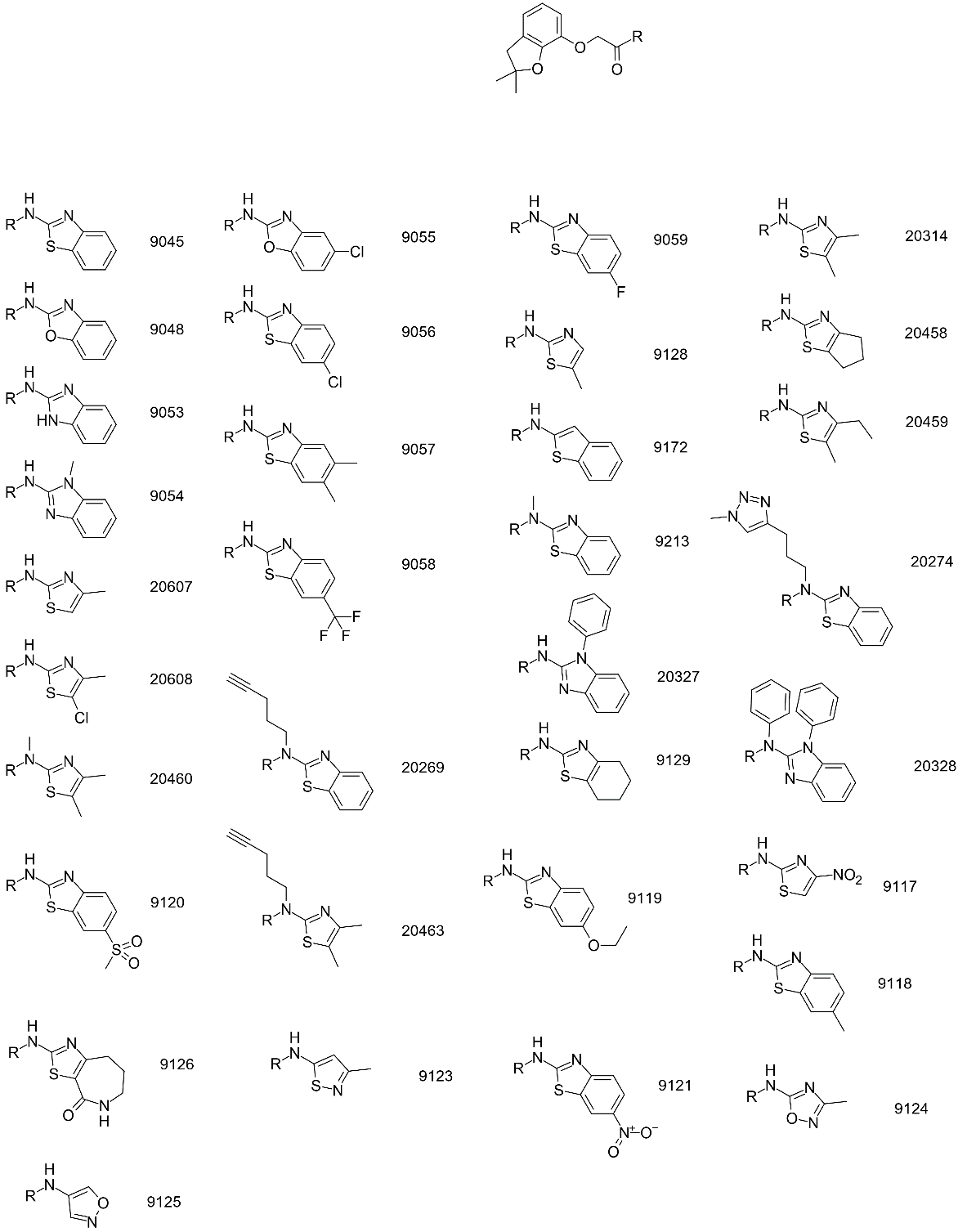 | 20819 | 39 [21-72] µM |
| 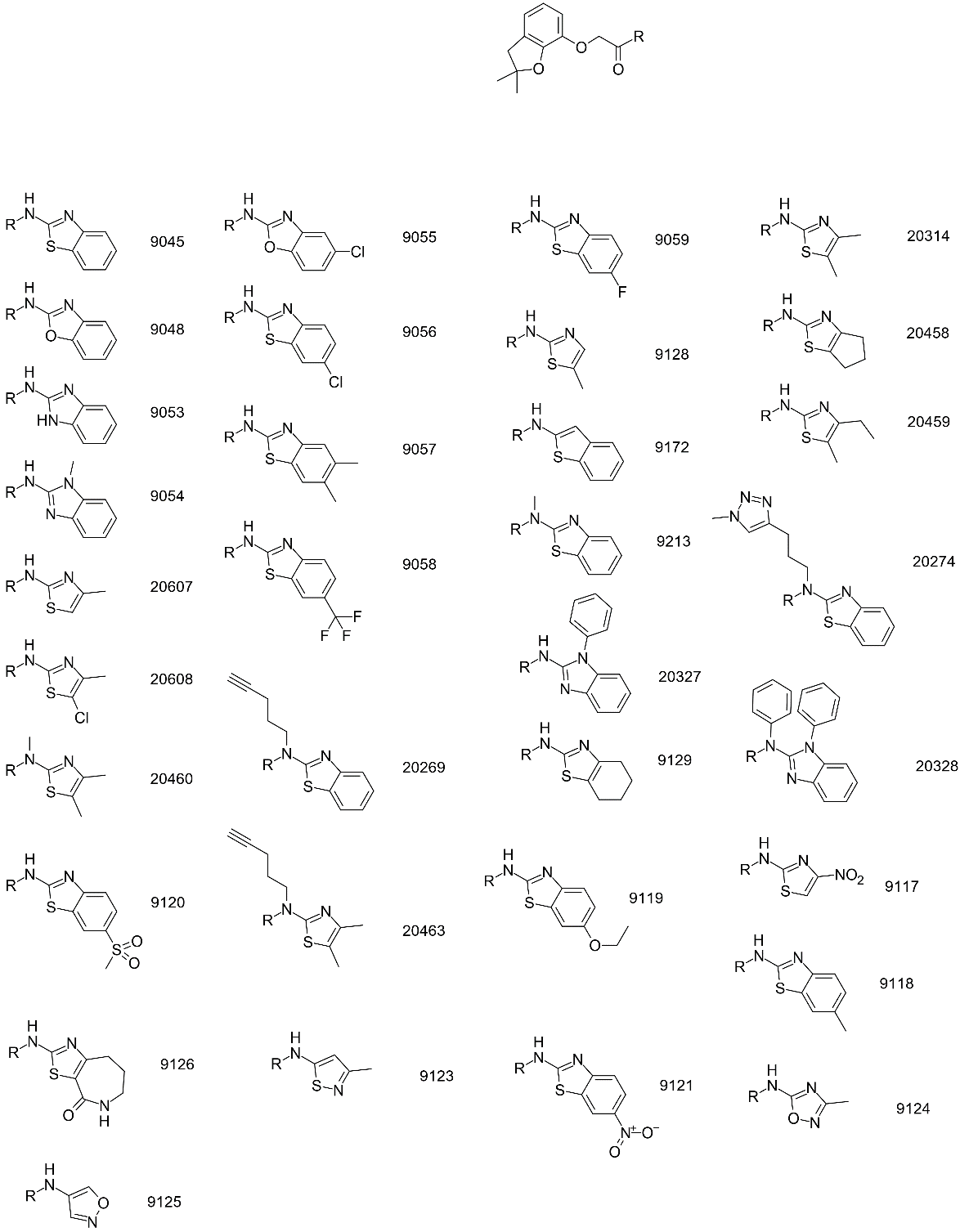 | 9128 | | 0.86 [0.53-1.40] µM | 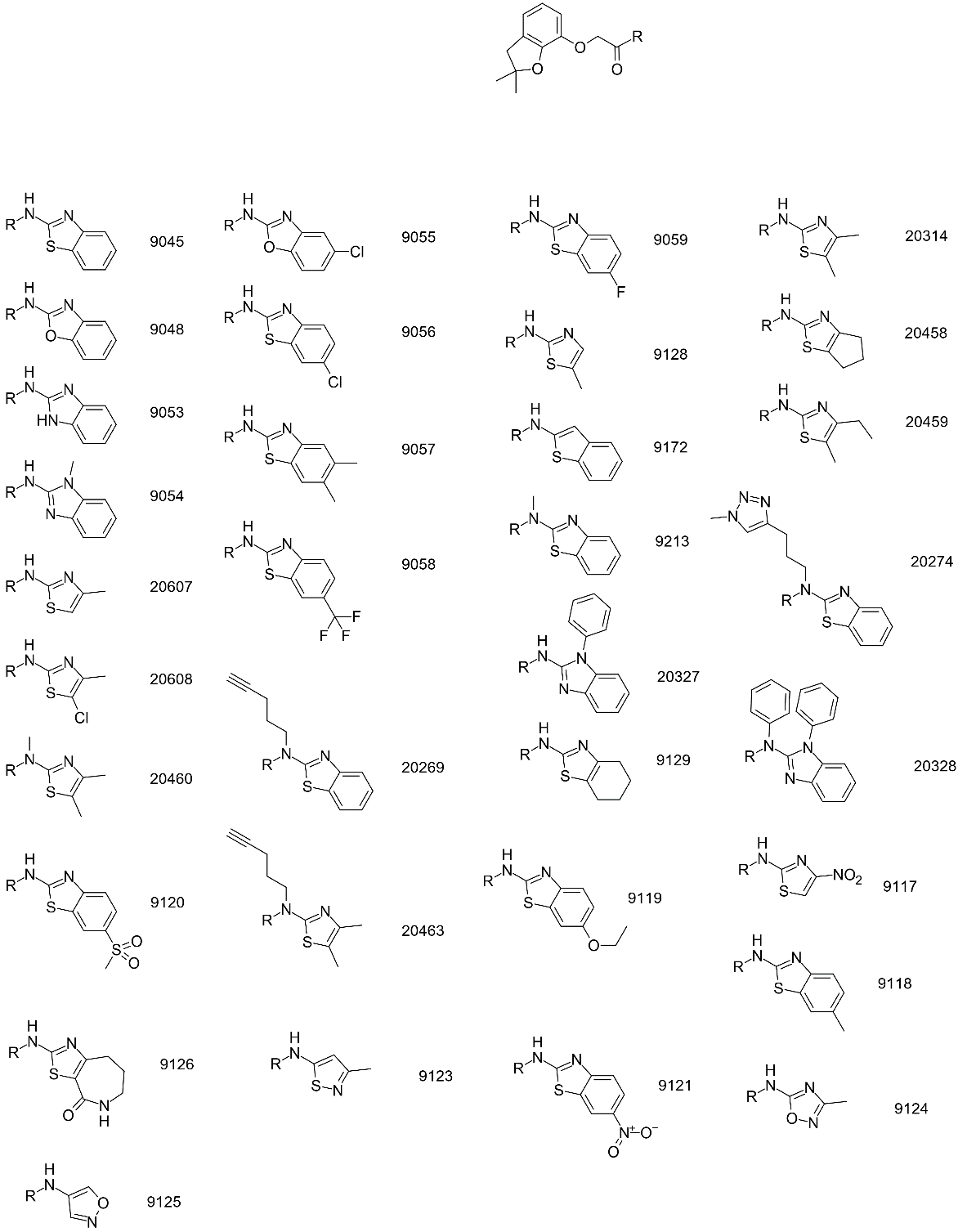 | 9117 | >100 µM |
|  |  | |  |  |  |  |
| **R =** | | **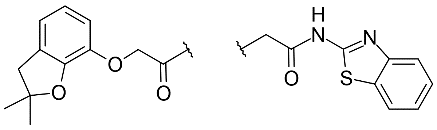** | | | | |
