# Supplementary material for: Highly Potent Host-Specific Small-Molecule Inhibitor of Paramyxovirus and Pneumovirus Replication with High Resistance Barrier
Source: mBio. 2021 Nov 2;12(6):e02621-21. doi: 10.1128/mBio.02621-21 (PMC8561388; doi:10.1128/mBio.02621-21)
Supplement: TABLE S3 [file mbio.02621-21-st003.docx]

| **Different aromatic hetero cycles** | | | | | | |
| --- | --- | --- | --- | --- | --- | --- |
| **Residue** | **ID** | | **IC_50_** | **Residue** | **ID** | **IC_50_** |
| 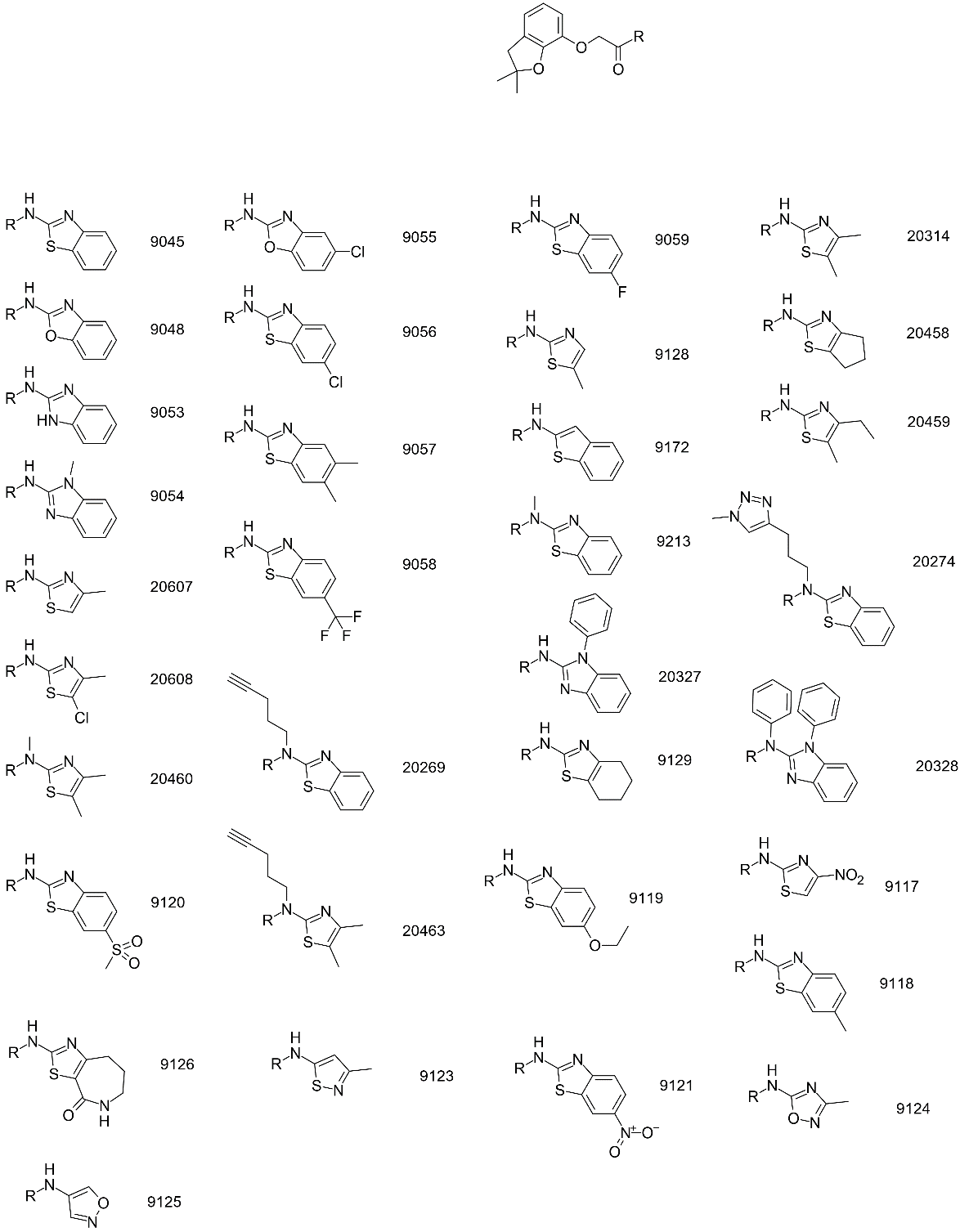 | 20327 | | 0.34 [0.28-0.41] µM | 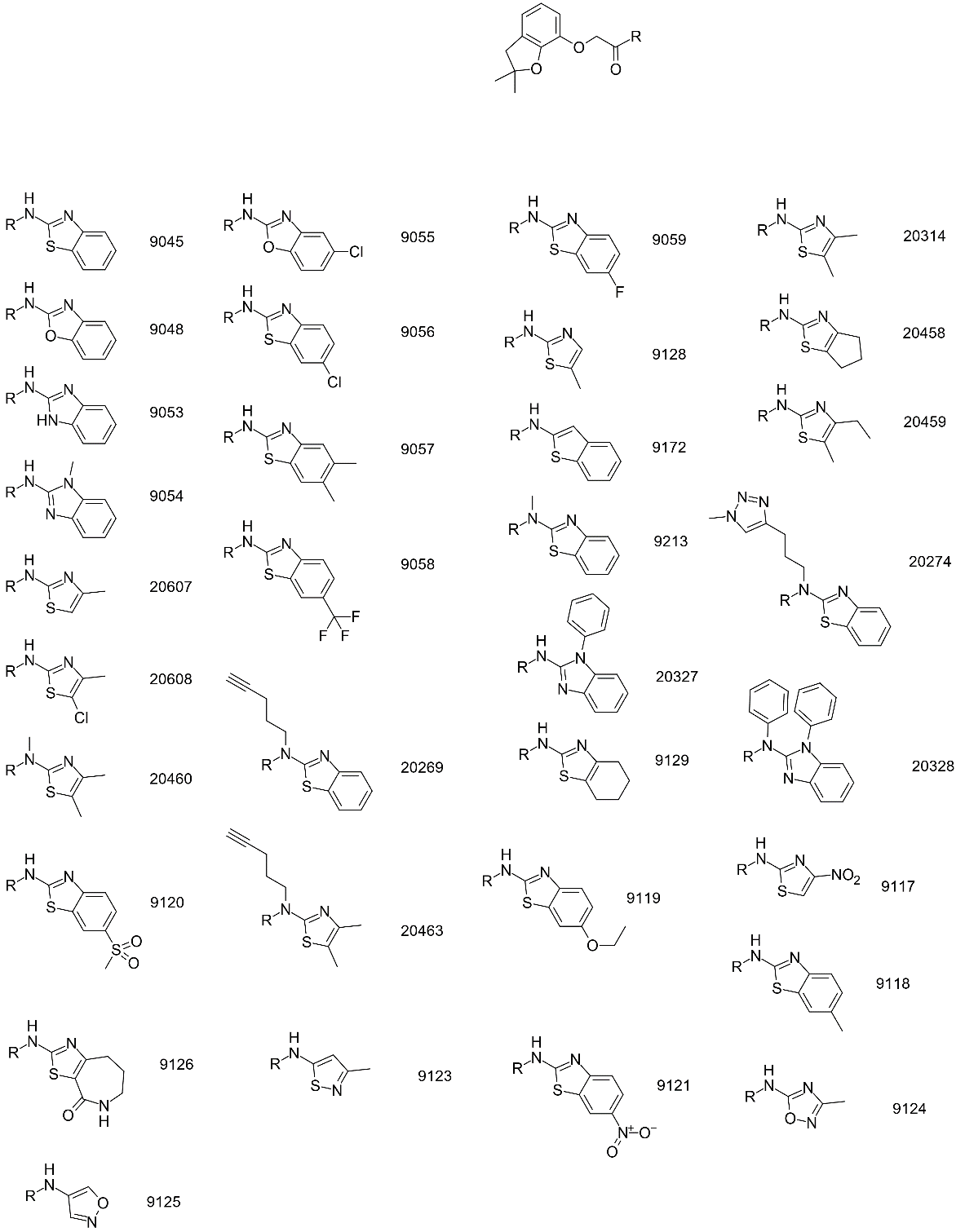 | 9123 | 85 [34-210] µM |
| 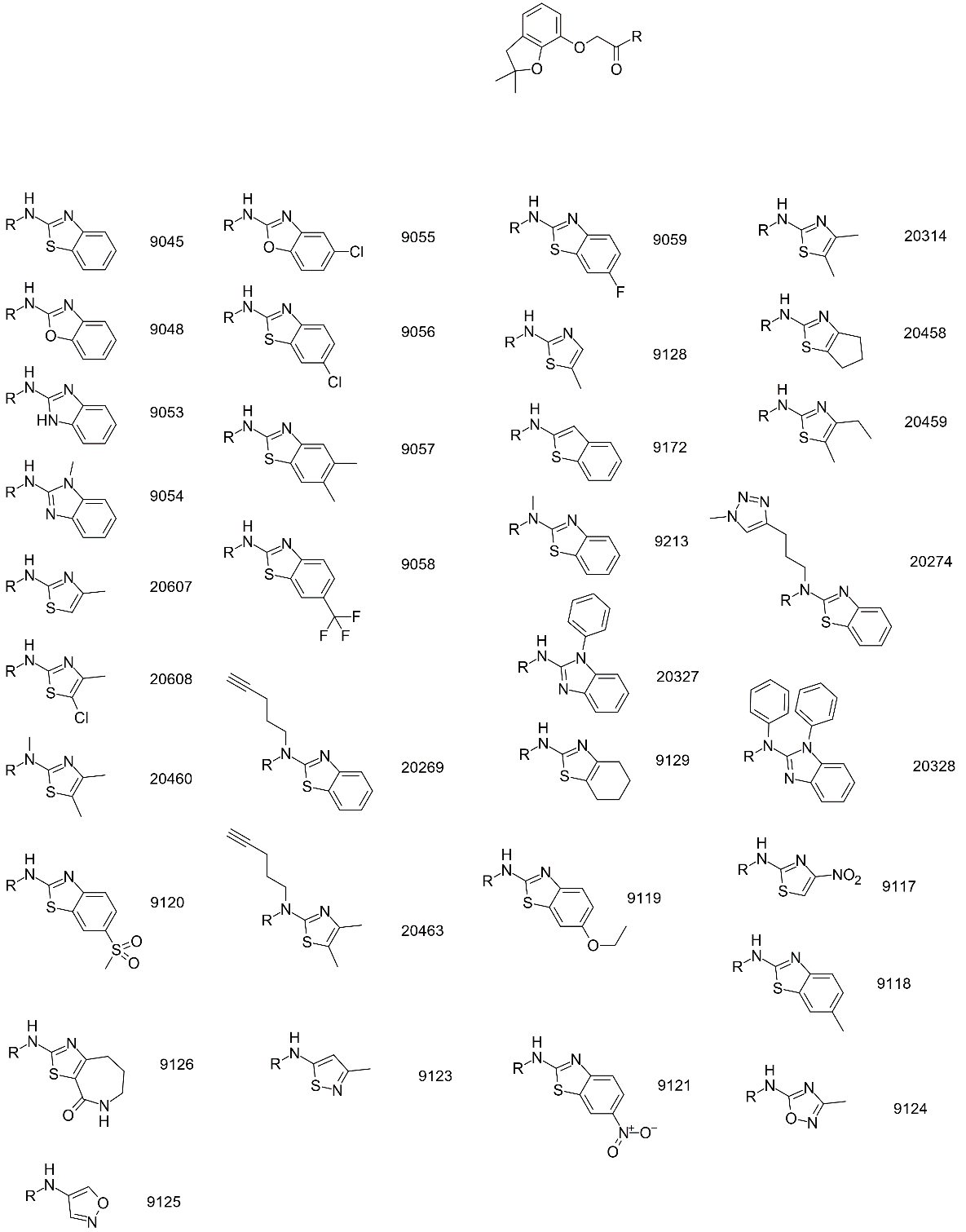 | 9054 | | 37 [30-52] µM | 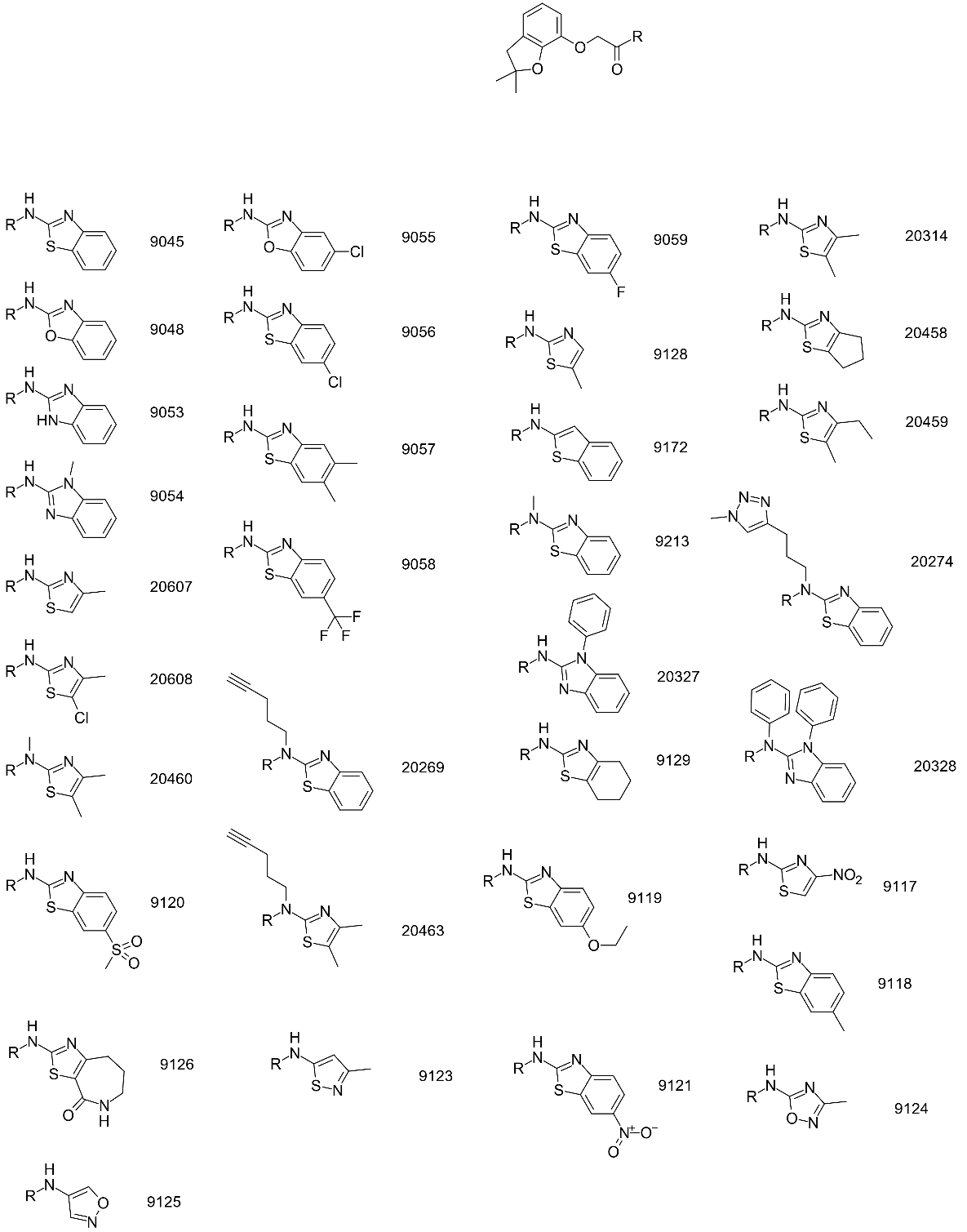 | 9172 | >100 µM |
| 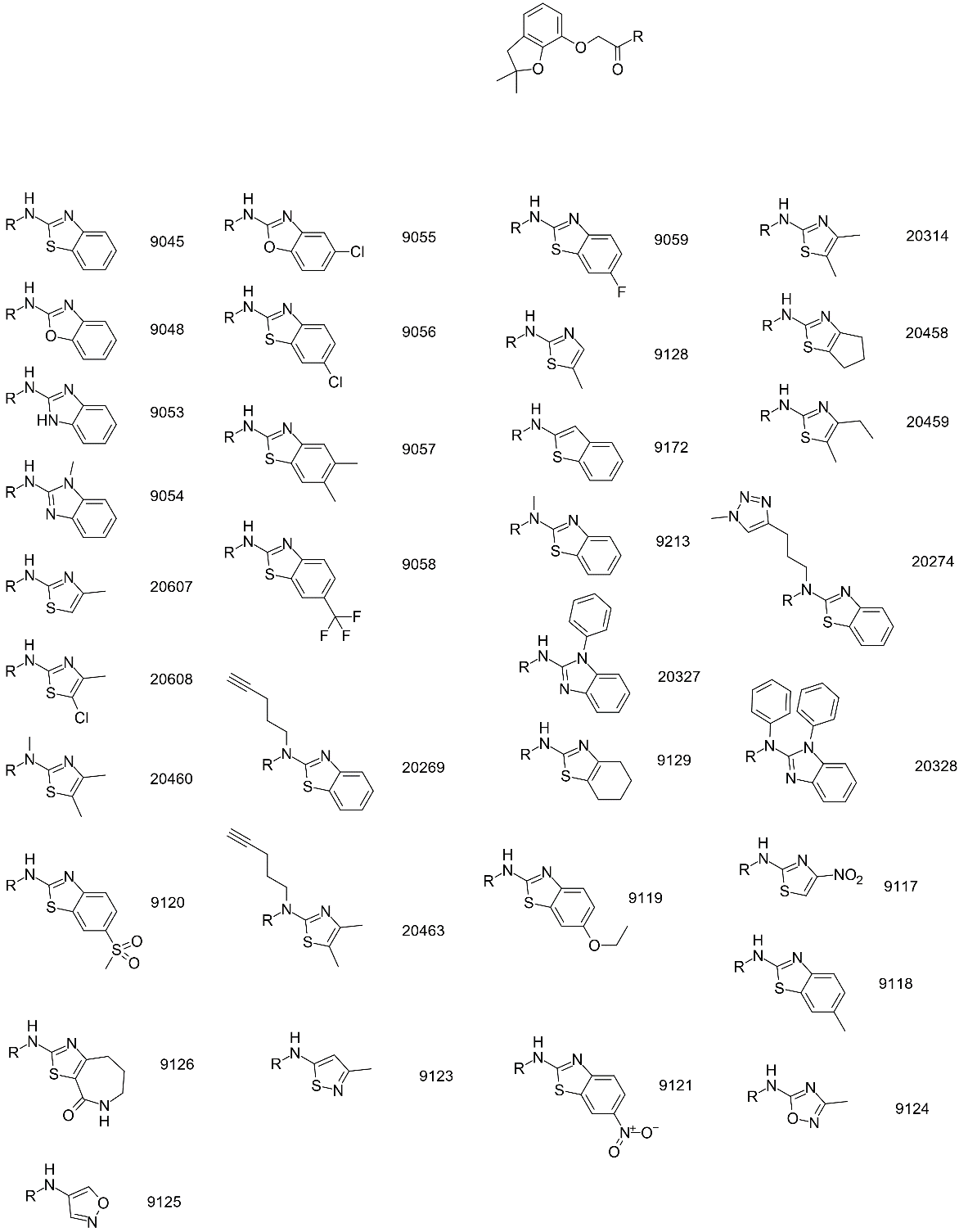 | 9048 | | 44 [30-67] µM | 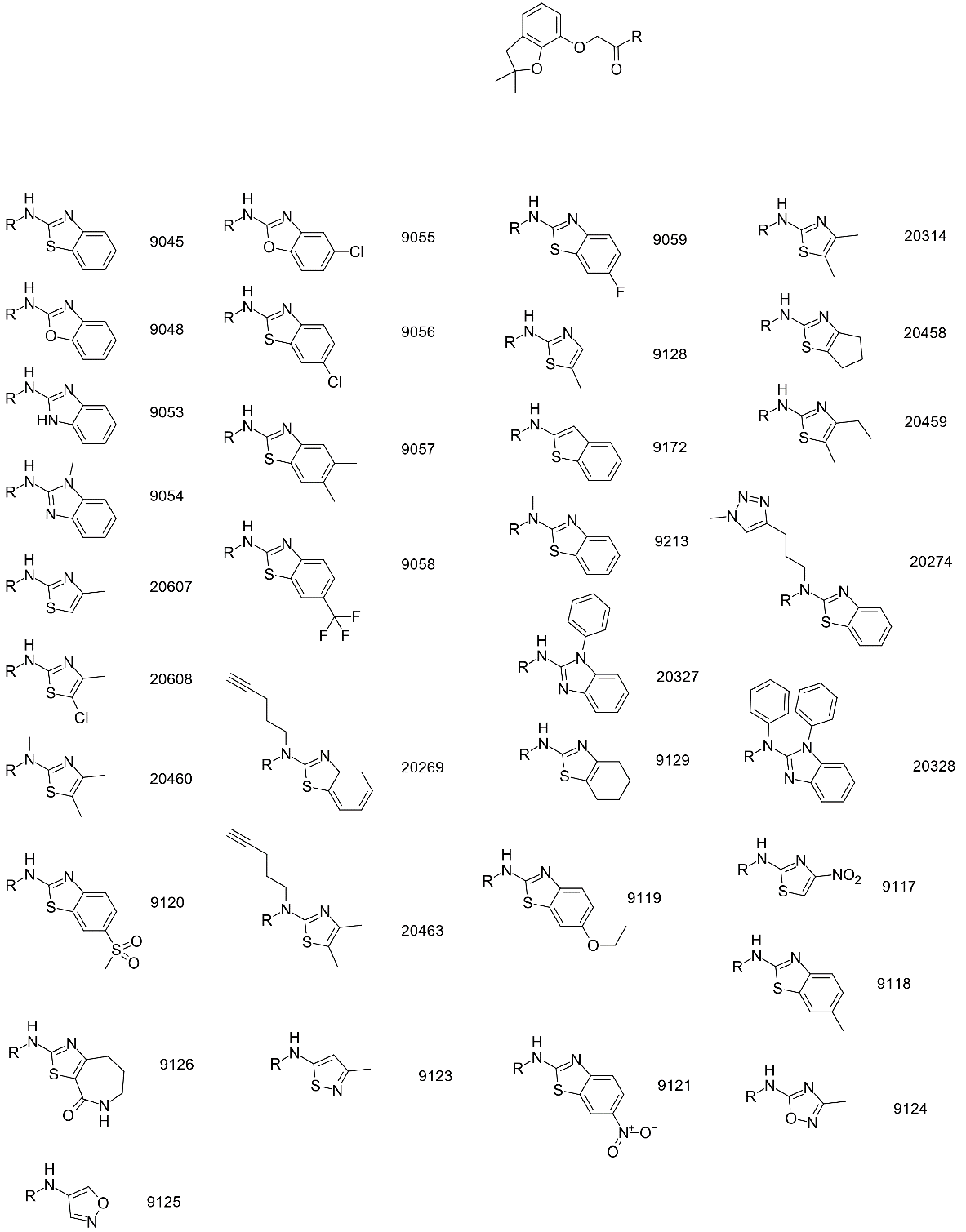 | 9124 | >100 µM |
| 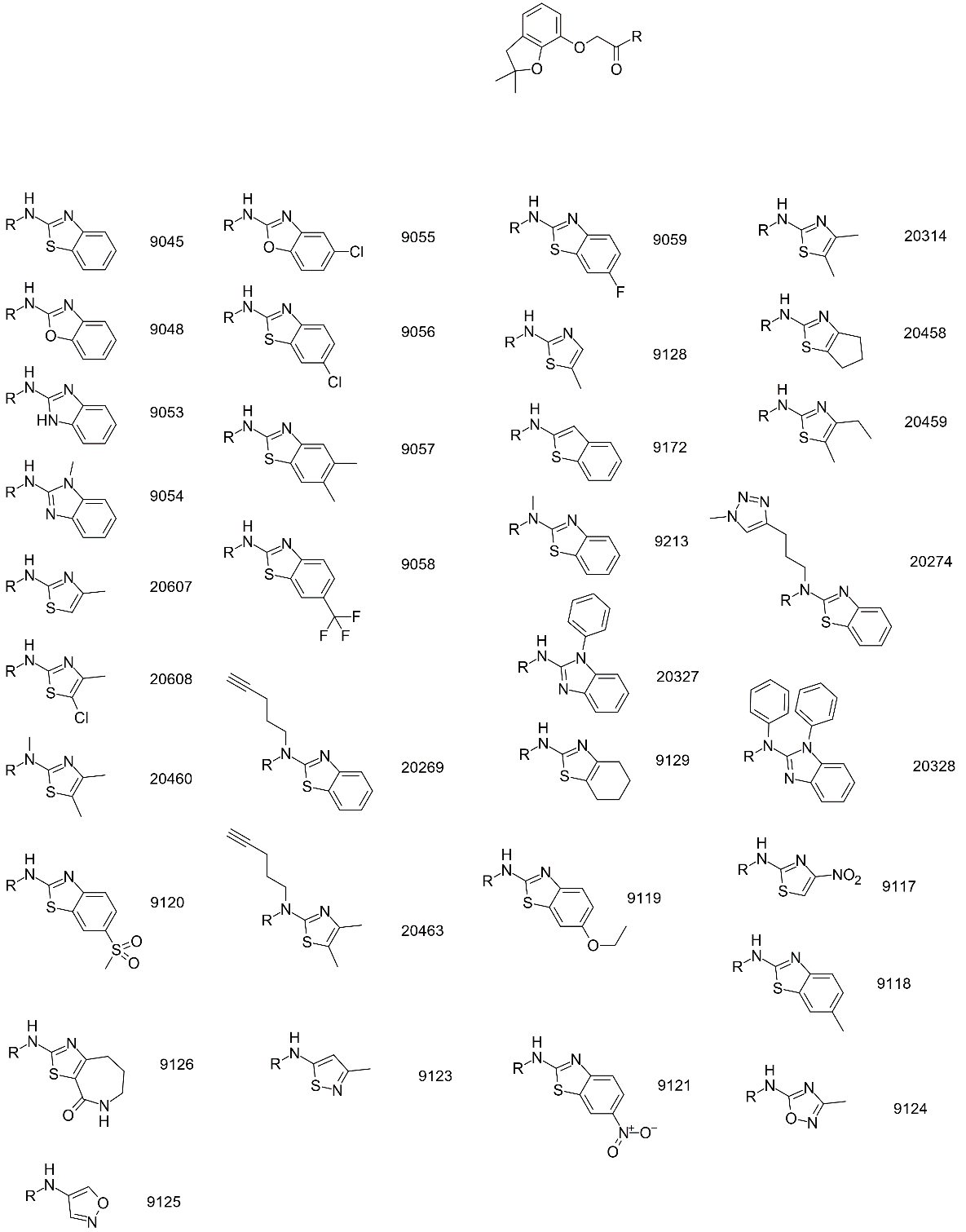 | 9055 | | 48 [38-71] µM | 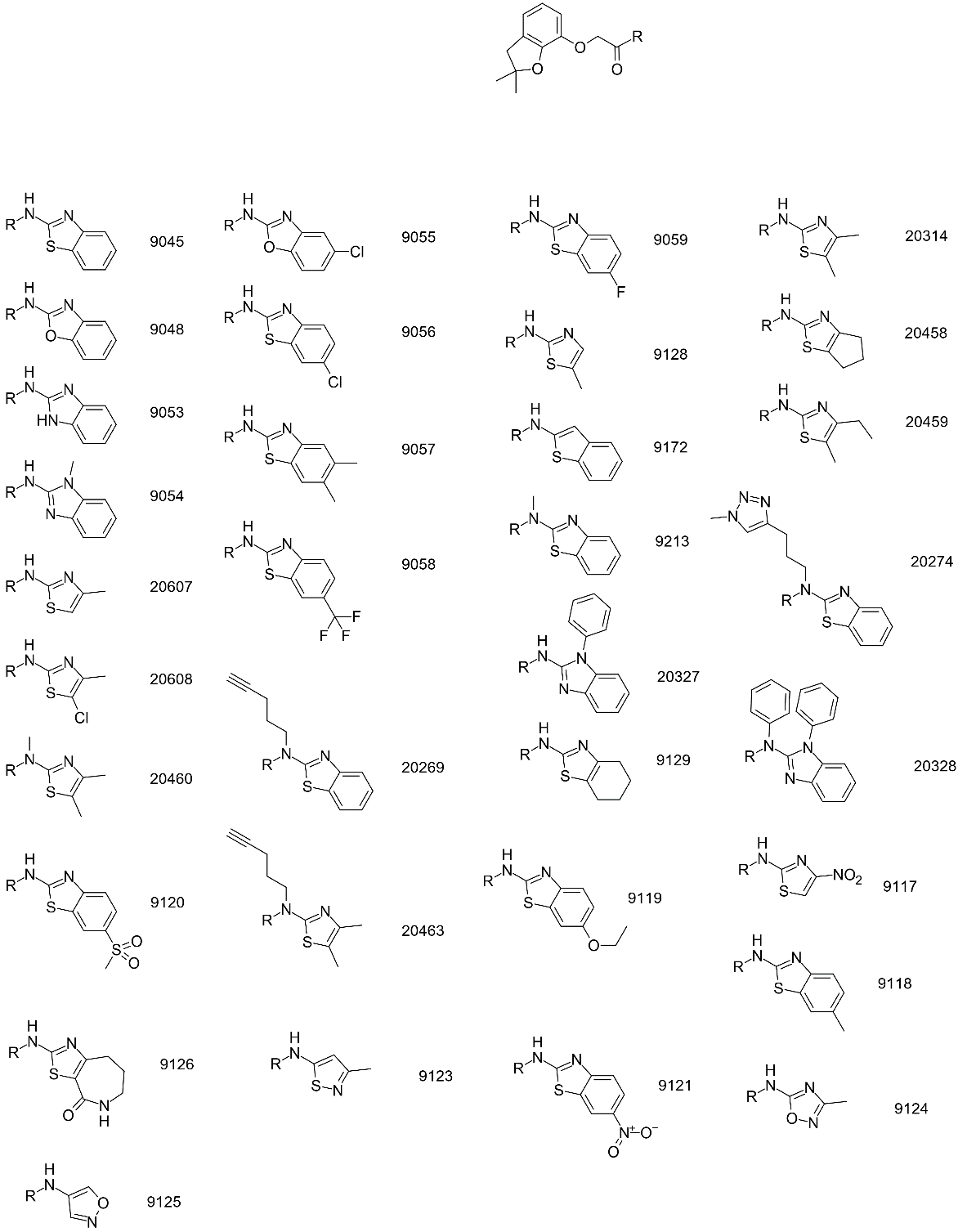 | 9125 | >100 µM |
| 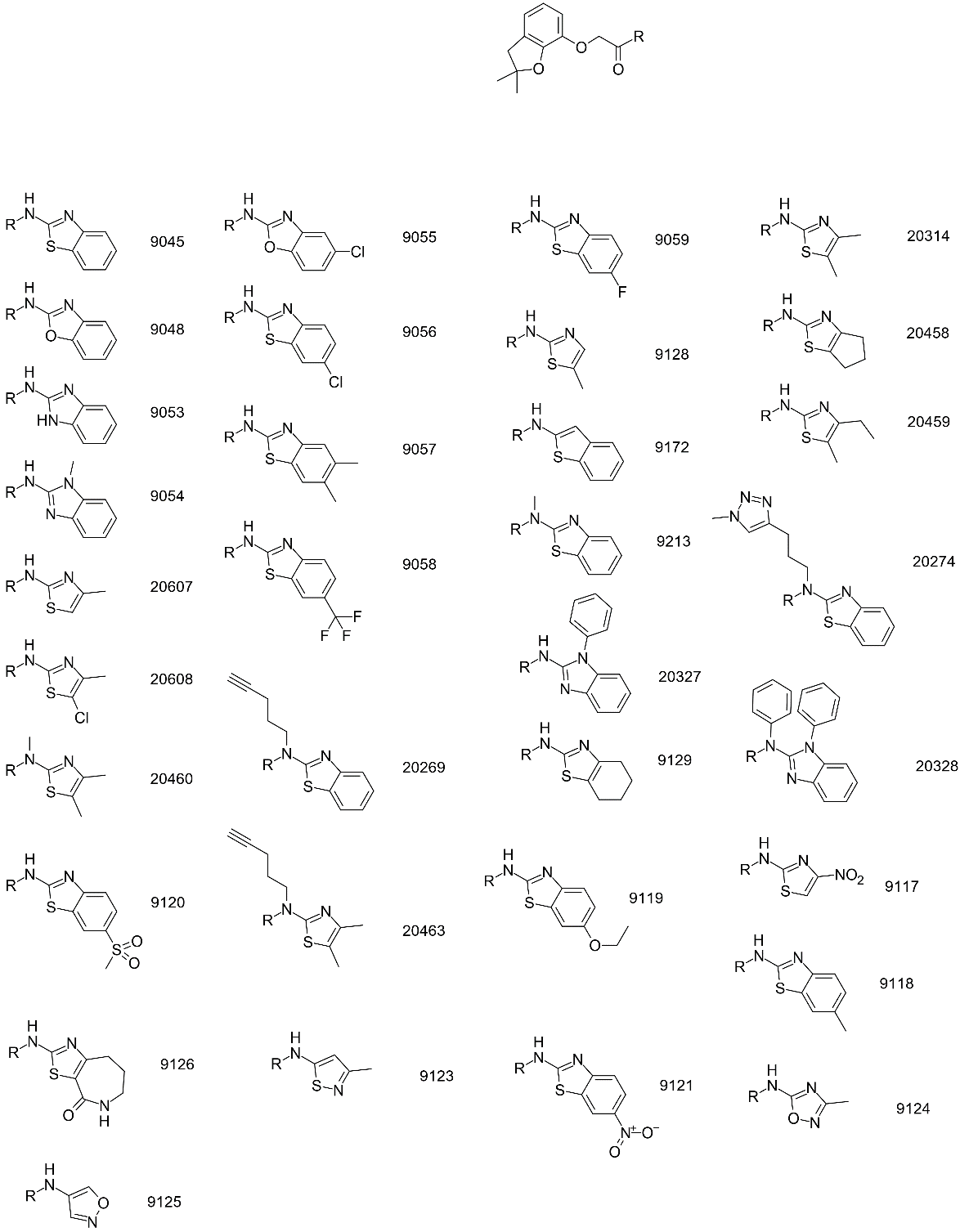 | 9053 | | 67 [44-94] µM |  |  |  |
| ***N*-alkylated aminthiazole** | | | | | | |
| **Residue** | **ID** | | **IC_50_** | **Residue** | **ID** | **IC_50_** |
| 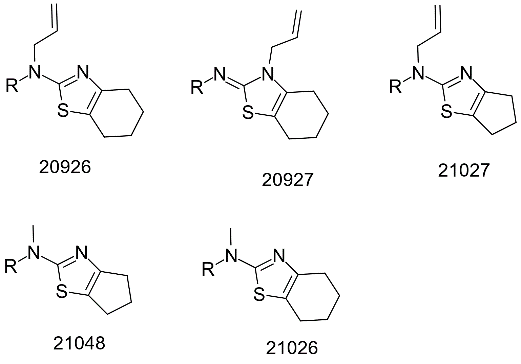 | 21027 | | 0.0041 [0.0026-0.0066] µM | 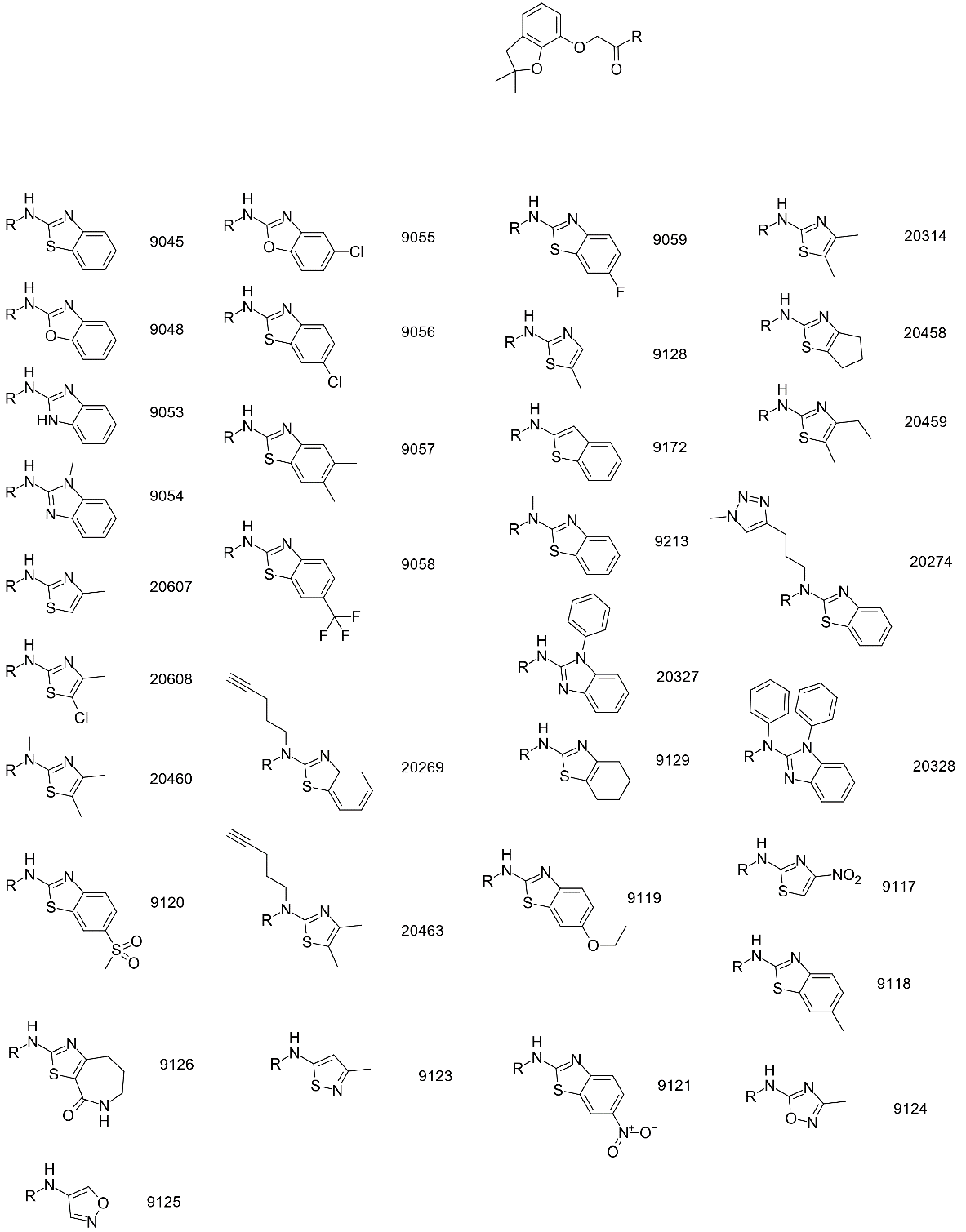 | 20269 | 0.27 [0.22-0.31] µM |
| 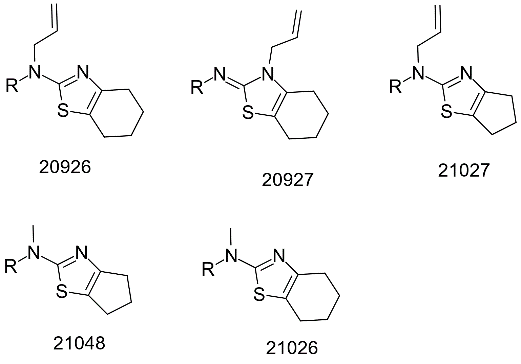 | 20926 | | 0.0082 [0.0062-0.011] µM | 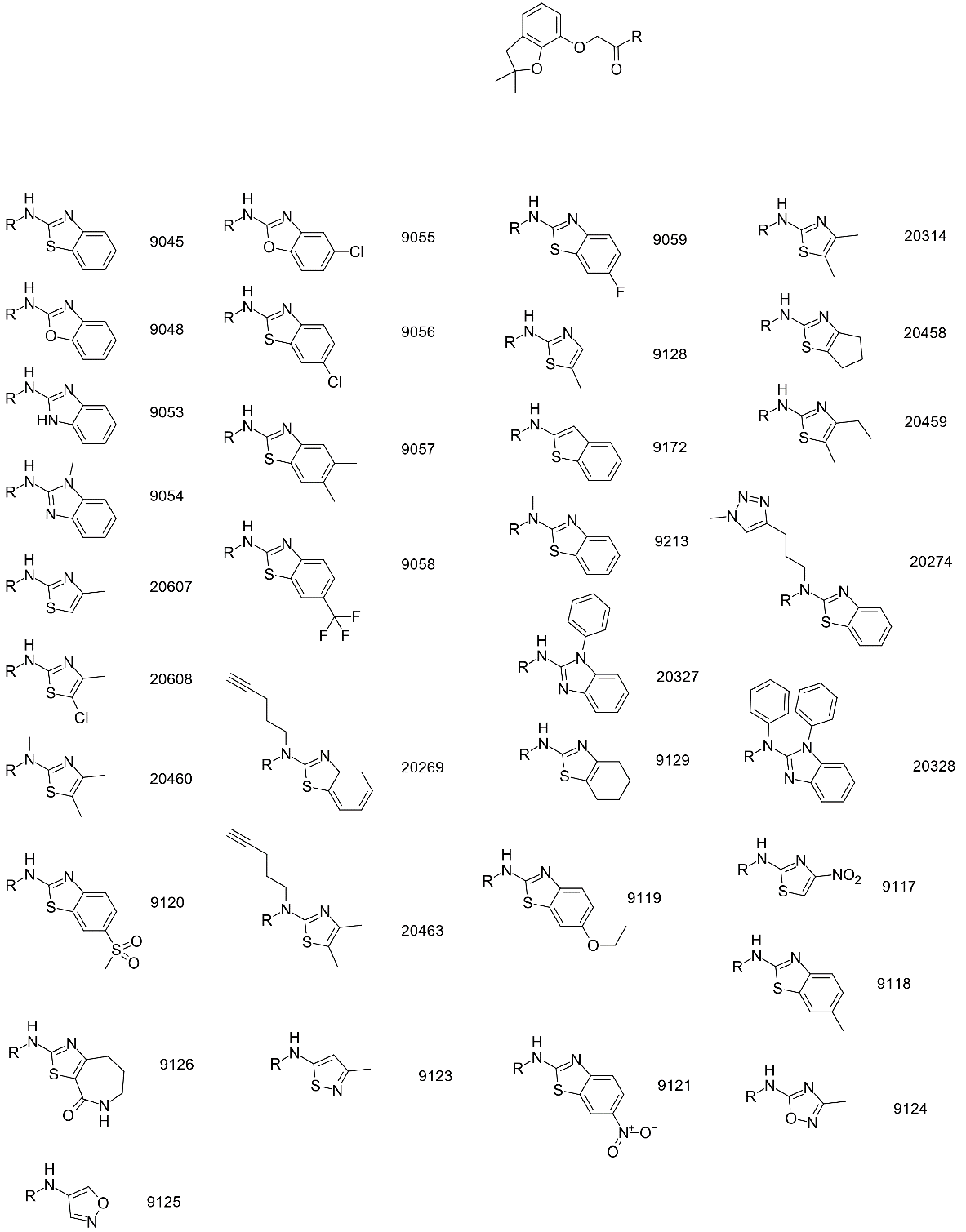 | 20460 | 1.7 [0.85-3.4] µM |
| 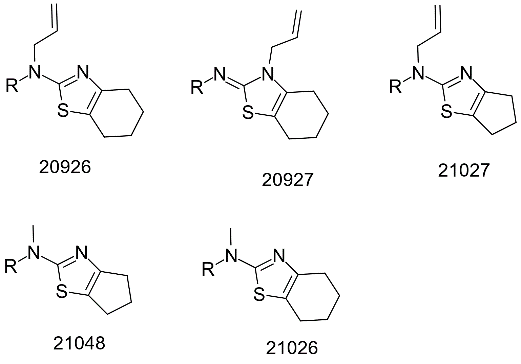 | 21026 | | 0.0084 [0.0058-0.012] µM | 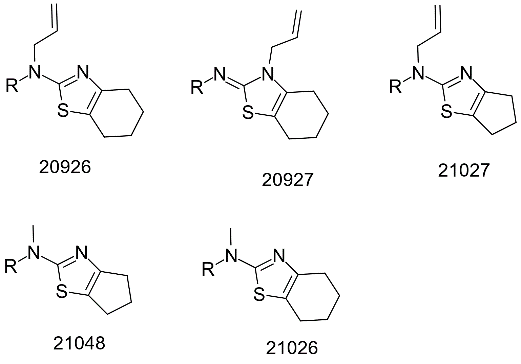 | 20927 | 29 [21-47] µM |
| 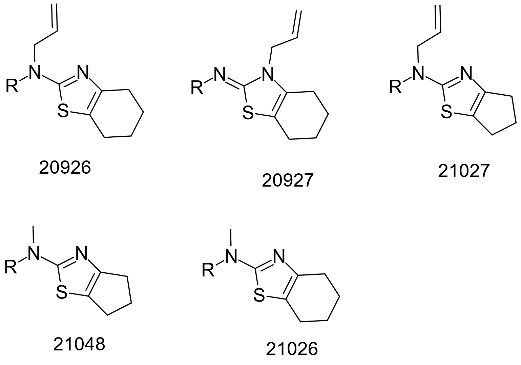 | 21048 | | 0.080 [0.069-0.091] µM | 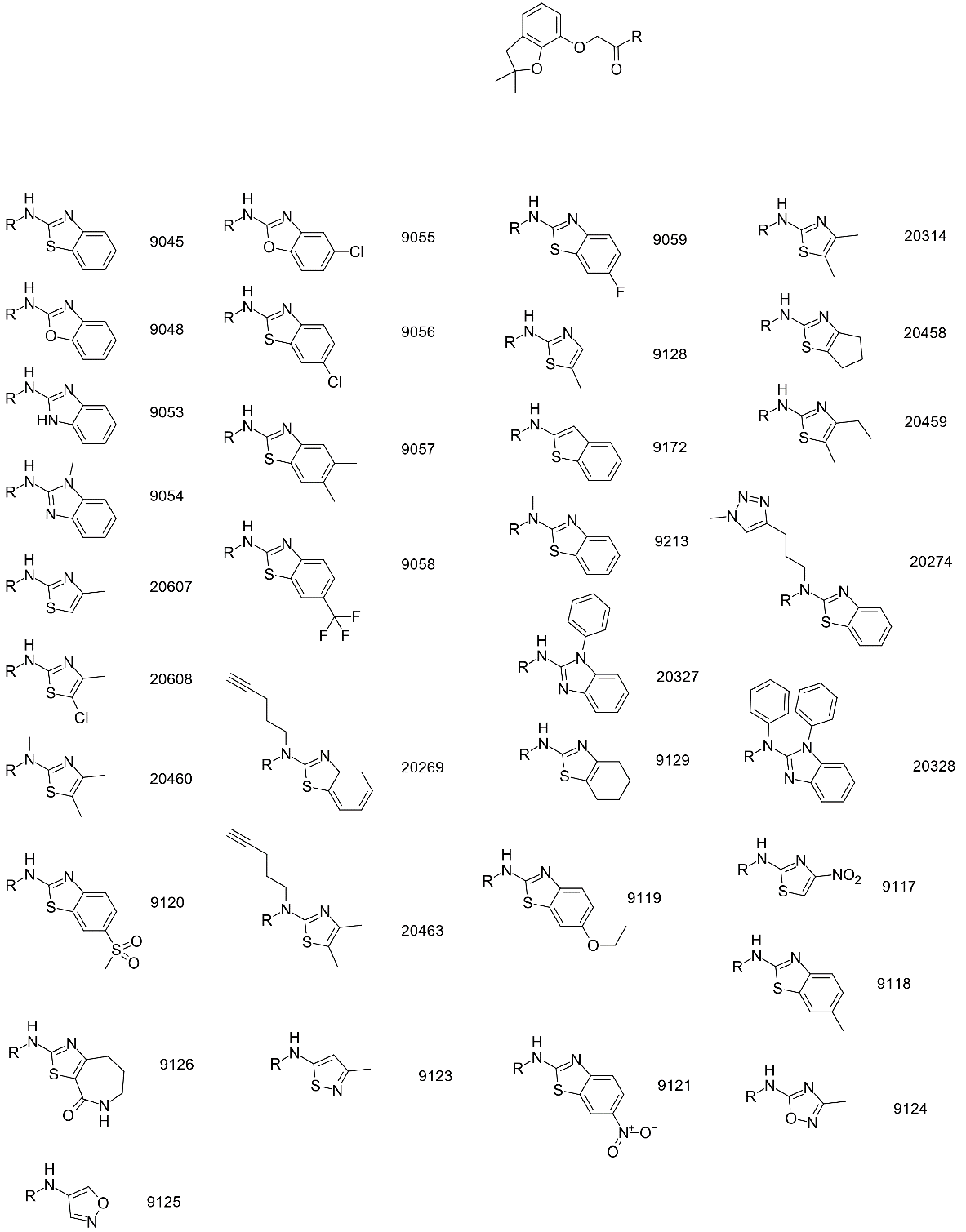 | 20328 | 30 [6.9-79] µM |
| 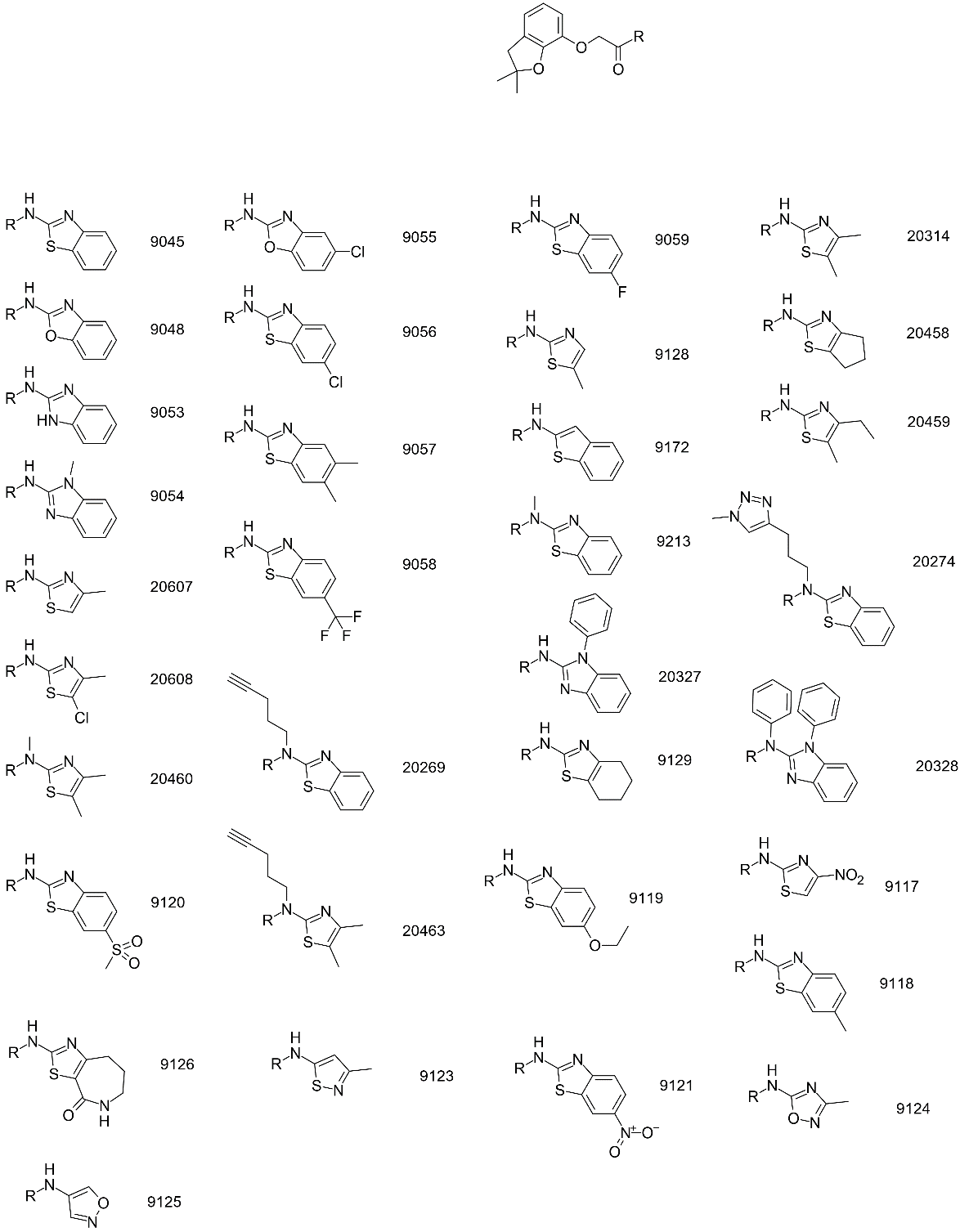 | 9213 | | 0.16 [0.13-0.19] µM | 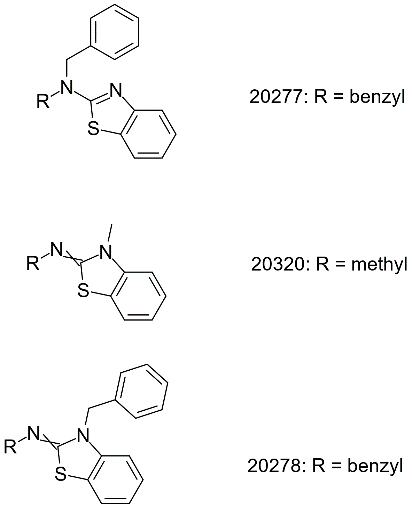 | 20278 | 34 [1.4-163] µM |
| 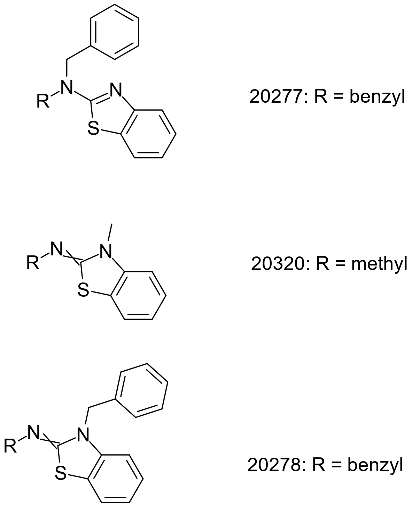 | 20277 | | 0.25 [0.20-0.32] µM | 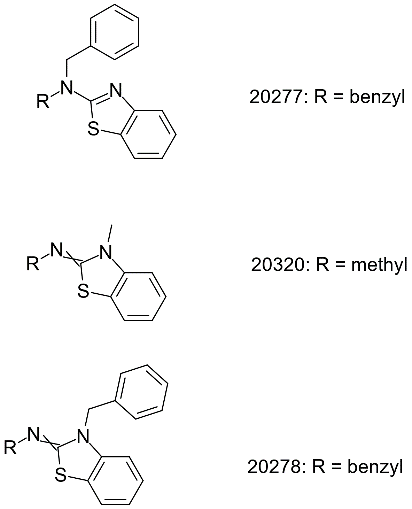 | 20320 | 56 [20-165] µM |
| 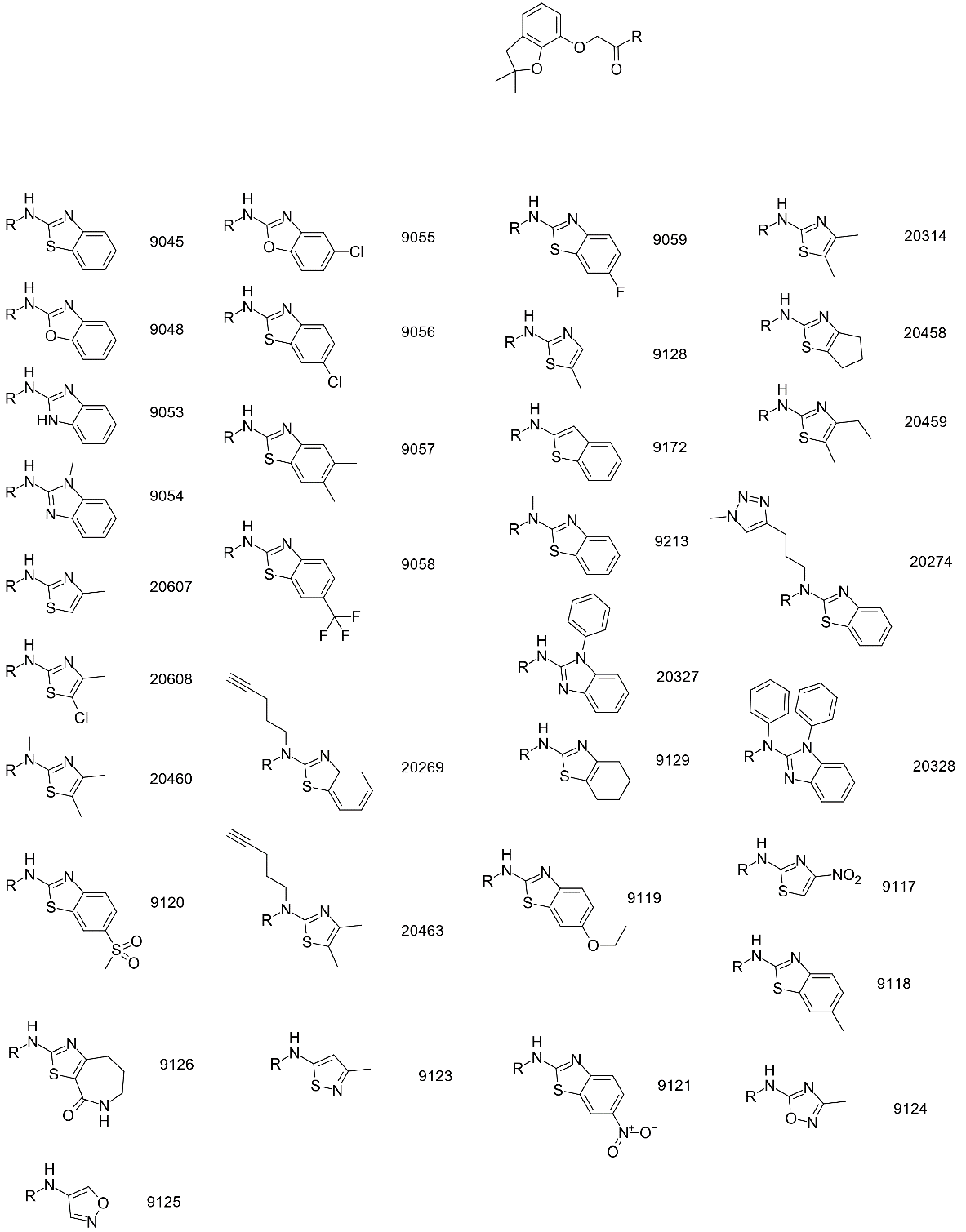 | 20463 | | 0.26 [0.18-0.38] µM |  |  |  |
| **R =** | | **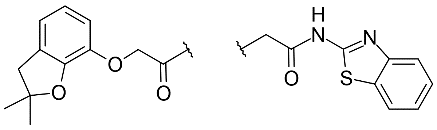** | | | | |
